# Red Blood Cell Transcriptome Reflects Physiological Responses to Alternative Nutrient Sources in Gilthead Seabream (*Sparus aurata*)

**DOI:** 10.3390/ani15091279

**Published:** 2025-04-30

**Authors:** Rafael Angelakopoulos, Andreas Tsipourlianos, Alexia E. Fytsili, Leonidas Papaharisis, Arkadios Dimitroglou, Dimitrios Barkas, Zissis Mamuris, Themistoklis Giannoulis, Katerina A. Moutou

**Affiliations:** 1 Laboratory of Genetics, Comparative and Evolutionary Biology, Department of Biochemistry and Biotechnology, University of Thessaly, Biopolis, 41500 Larissa, Greece; rangelak@uth.gr (R.A.); antsipou@uth.gr (A.T.); afytsili@uth.gr (A.E.F.); zmamur@uth.gr (Z.M.); 2 Avramar Aquaculture SA, 19002 Athens, Greece; lpapaharisis@gmail.com (L.P.); a.dimitroglou@aua.gr (A.D.); d.barkas@avramar.eu (D.B.); 3 Laboratory of Applied Hydrobiology, Department of Animal Science, Agricultural University of Athens, Iera Odos 75, 11855 Athens, Greece; 4 Laboratory of Biology, Genetics and Bioinformatics, Department of Animal Science, University of Thessaly, Greece Gaiopolis, 41334 Larissa, Greece; thgianno@uth.gr

**Keywords:** sustainable aquaculture, fishmeal alternatives, transcriptome analysis, gilthead seabream, physiological responses, minimally invasive sample collection

## Abstract

The sustainable growth of finfish farming relies heavily on reducing the high ecological footprint of sourcing and producing fish feeds that accounts for almost 50% of the total ecological footprint of finfish farming. Sustainable alternatives to fishmeal often pose challenges due to the presence of antinutritional factors and nutrient imbalances that impair fish health and growth. Screening for alternative nutrient sources and adapting to global commodity fluctuations requires modern tools that can predict the physiological responses of fish early and reliably. The present study explores for the first time the potential of fish red blood cell (RBC) transcriptome as a minimally invasive biomarker of physiological responses in gilthead seabream (*Sparus aurata*) fed either a fishmeal-based (FM) or a plant-protein-based (PP) diet. Blood samples were collected at multiple time points (15, 20, and 30 days post-diet initiation) from genetically diverse full-sib families reared under commercial conditions, integrating transcriptomic analysis with long-term growth assessments. Differential gene expression analysis revealed significant dietary effects on oxidative phosphorylation, ribosomal capacity, and lipid metabolism pathways, highlighting metabolic plasticity and cellular adaptations to plant-based feeds. The downregulation of oxidative phosphorylation genes suggests a metabolic shift in response to altered nutrient composition, while ribosomal pathway modulation indicates potential constraints on protein synthesis. These transcriptomic shifts, conserved across two independent experiments, reinforce the utility of RBCs as a real-time indicator of fish physiological status, offering a tool for monitoring dietary impacts and optimizing feed formulations. Such insights are essential for advancing sustainable, nutritionally balanced aquaculture feeds that support fish welfare and productivity. The minimally invasive sample collection respects the 3Rs (Reduce, Refine, Replace) principle in animal experimentation and allows for frequent screening and generation of refined data.

## Simple Summary

This study explores how red blood cell (RBC) transcriptomics can be used as a minimally invasive biomarker to assess the effects of plant-based diets in gilthead seabream (*Sparus aurata*). Fish were fed either a fishmeal-based (FM) diet or a plant-protein-based (PP) diet, and blood samples were collected at different time points to analyze gene expression changes. The results showed that the PP diet affected energy metabolism, protein synthesis, and lipid processing, with oxidative phosphorylation genes being downregulated, suggesting a shift in energy use. These findings highlight the potential of RBC transcriptomics for monitoring fish health and optimizing feed formulations, supporting more sustainable and nutritionally balanced aquaculture. The approach also aligns with the 3Rs principle by reducing stress on fish while enabling frequent health assessments.

## 1. Introduction

The sustainable development of aquaculture hinges on advancing novel feeding strategies that reduce reliance on traditional marine ingredients, such as fishmeal. These ingredients are declining rapidly due to overexploitation and climate change, leading to unsustainable availability and costs for the sector [1]. Therefore, there is an urgent need for highly nutritive and cost-effective feeds that also support fish welfare [2,3,4]. Among the most promising alternatives are plant-derived proteins, which have expanded significantly over the last decade due to their availability, cost-effectiveness, and stable supply from a wide range of sources like soybean, wheat, and barley. Their use is also driven by the need to reduce reliance on marine resources due to climate change and for a sustainable management of fish stocks [5,6,7]. However, their application raises several concerns. Plant ingredients often contain antinutritional factors (ANFs)—such as phytates, tannins, saponins, and protease inhibitors—as well as non-starch polysaccharides and other non-digestible carbohydrates, which can adversely affect nutrient absorption, gut health, and overall metabolism in fish [8,9,10,11]. Peripheral blood, particularly red blood cell (RBC) transcriptomics, provides a minimally invasive and practical tool for studying physiological responses to dietary shifts [12]. Plant-based diets have been shown to alter the expression of genes involved in essential cellular pathways in red blood cells (RBCs) of gilthead seabream (*Sparus aurata*), an emblematic species of Mediterranean aquaculture, with a production volume of 103,130 tonnes in the EU-27 [13,14,15]. These diets may disturb pathways such as oxidative phosphorylation (OxPhos), ribosome biogenesis, metabolic processes, and the proteasome/proteolysis system. Given that these pathways are closely linked to energy production, protein synthesis, and degradation—processes fundamentally driven by nutrients supplied from feeds—dietary shifts from fishmeal to plant proteins can compromise energy metabolism and cellular homeostasis. Such disruptions may lead to reduced growth performance and impaired physiological adaptation, highlighting the need to understand transcriptomic responses to alternative diets. This approach also aligns with the 3Rs (Reduce, Refine, Replace) principle in animal experimentation, reducing stress on fish while allowing for dynamic monitoring of physiological changes over time. Several studies highlight RBC transcriptomics as a powerful method for assessing physiological changes in animals [16,17]. In modern aquaculture, key traits such as growth potential and dietary response are typically assessed only at the end of the production cycle, requiring extended rearing periods before meaningful evaluations can be made. This delay hinders early selection of robust phenotypes and timely optimization of feeding strategies [5]. Consequently, there is growing interest in identifying early, minimally invasive biomarkers that can reflect physiological adaptations to diet. Peripheral blood, particularly RBCs, has emerged as a promising tissue for transcriptomic analysis, offering real-time insights into the metabolic and physiological status of fish [18]. Despite this potential, the application of RBC transcriptomics to monitor long-term dietary adaptation in aquaculture species remains limited [13].

RNA sequencing is the preferred method due to its high reproducibility and accuracy in quantifying gene expression across tissues. Our earlier work demonstrated the potential of nonlethal transcriptomic approaches to predict long-term growth outcomes in gilthead seabream (*S. aurata*). Using twenty full-sib families, we compared fishmeal-based (FM) and plant protein-based (PP) diets. By integrating zootechnical, biochemical, and erythrocyte transcriptomic data from early sampling points (15 and 30 days post-diet initiation), we identified molecular and biochemical markers that accurately distinguished faster- and slower-growing fish. These findings emphasized the value of early gene expression responses as biomarkers in selective breeding; as different genetic backgrounds shape responses to environmental challenges including dietary changes, they allow for the identification of biomarkers linked to traits such as growth rate, feed efficiency, and overall health. This early molecular insight enables breeders to select individuals with favorable signatures, reducing time and cost in phenotypic evaluations while preserving genetic diversity and driving genetic gains in sustainable aquaculture [13]. Building on this foundation, the present study takes a step further in exploring the responses of RBCs to dietary challenges.

Two feeding experiments were conducted in consecutive years in a commercial setting and for a full farming cycle to assess (a) how the type of diet (FM vs. PP) drives significant changes in RBC transcriptomics, (b) how the genetic background influences transcriptomic responses, (c) the duration of the responses, and (d) how consistent the responses are. In the first experiment, the transcriptomic changes in RBCs of six full-sib families from a commercial breeding program at two early time points were compared. To evaluate the consistency of the findings, the second experiment was a replication study with three different full-sib families from the same breeding program and a single time point. By integrating data from both experiments, this study identifies common gene networks and KEGG pathways underpinning early response of RBCs to dietary changes across genetic backgrounds.

## 2. Materials and Methods

### 2.1. Ethics Statement

All examined biological materials were derived from fish reared and harvested at commercial farms, registered for aquaculture production in EU countries. Animal sampling followed routine procedures, and samples were collected by a qualified staff member from standard production cycles. The legislation and measures implemented by the commercial producers complied with existing national and EU (Directive 1998/58/EC) legislation (protection of animals kept for farming).

### 2.2. Experimental Fish

Juvenile gilthead seabream (*S. aurata*) used in this study originated from AVRAMAR SA’s commercial family-based breeding program. The program follows a structured genetic selection strategy, producing 100 new families every year through artificial crosses of preselected breeding candidates. Selection is based on key traits such as growth performance, disease resistance, and body shape. To maintain genetic diversity and avoid inbreeding, crosses are performed between individuals from different year classes, with a generation interval of approximately four years due to the different timing of achieving reproductive maturity between males and females. Fish were individually tagged with intraperitoneal RFID glass tags for pedigree tracing. After tagging, they were transferred to a commercial sea cage farm in Paleros, Aitoloakarnania, Greece, for the feeding trial.

#### 2.2.1. Experiment 1

A total of 1000 fish (n = 1000) were selected for the first experiment. These fish originated from 20 full-sib families that were specifically chosen from the 100 families produced that year by the breeding program. These families were selectively bred from fish exhibiting extreme phenotypes. Ten families (F01–F10) were generated from progeny with high weight gain and low intrafamilial variability (CV: 10–12%). Another ten families (F11–F20) were generated from progeny with low weight gain and high intrafamilial variability (CV: 25–26%). These criteria were used to capture families with contrasting phenotypes, facilitating investigation of the transcriptomic basis of growth performance and uniformity.

Fish from the 20 families were randomly selected and evenly distributed into two replicate sea cages (4 × 4 × 4 m depth). Fifty fish from each family were assigned equally between the two cages, with 25 fish per family in each cage. Both cages were fed the same commercially available feed (Feedus Blue Line 3.5 mm) during the acclimatization period. The feeding trial began a week after the fish were moved to the sea cages and after a one-day fasting period. One sea cage was fed the FM diet containing 30% marine animal protein and used as control, whereas the other group was fed the PP diet containing 85% plant protein. The formulation of the two feeds is shown in Table 1. The detailed formulation and composition of the two feeds can be found in [13].

Two samplings were conducted, one at fifteen days (D15) and another at thirty days (D30) after the transition to the experimental feeding regimes. Fish were fed to apparent satiation daily throughout the experimental period. To minimize the effect of subjectivity on feeding saturation, two individuals carried out the feeding, alternating each day.

**Figure 1:**
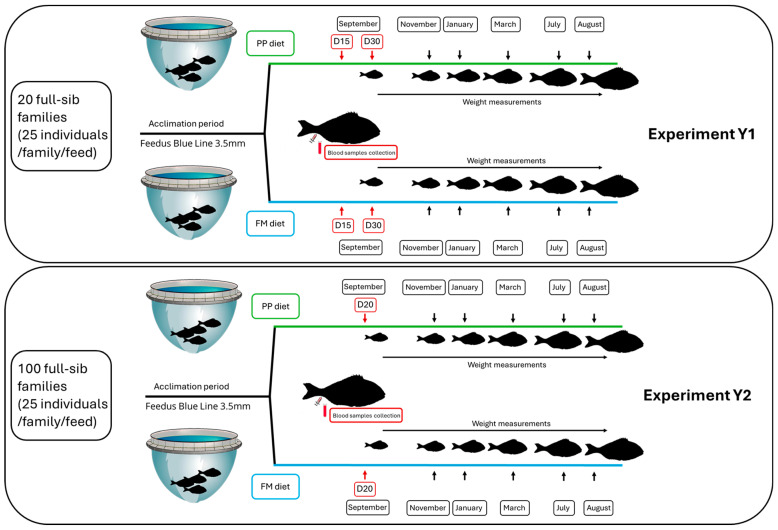
Experimental design of the two experiments. Red arrows denote the timing of blood samplings, and black arrows denote the timing of weight measurements.

The two groups were monitored until the fish reached an average harvest weight of approximately 400 g, at which point both mortality and body weight were recorded. Weight measurements were conducted in September, November, January, March, July, and August for all available individuals. Since all fish were PIT-tagged, individual weights were tracked throughout the trial, allowing for precise growth monitoring, and the specific growth rate (SGR) of each fish was calculated. The experimental design is illustrated in Figure 1.

#### 2.2.2. Experiment 2

In the second experiment, a total of 5000 fish derived from a total of 100 full-sib families were randomly selected and evenly allocated between two replicate sea cages (4 × 4 × 4 m depth). For each family, 50 individuals were distributed equally across the two cages, with 25 fish per family placed in each cage. The same feeds and feeding scheme as in experiment 1 were applied.

A single sampling was performed twenty days (D20) after the transition to the experimental feeding regimes. Fish were fed to apparent satiation daily, following the same protocol as in experiment 1, with two individuals alternating feeding duties each day to reduce subjectivity. The two groups were monitored until the fish reached a harvest weight of approximately 400 g, at which point both mortality and weight were recorded. Weight was recorded as in experiment 1, and SGR was calculated (Figure 1).

### 2.3. Blood Sampling

In both experiments, all available fish were carefully removed from the sea cages using a net and immediately transferred into a mild anesthetic solution (500 µL*L^−1^ ethylene glycol monophenyl ether) to facilitate blood sampling from the caudal vessel. Blood samples were centrifuged (6000× *g* for 5 min) to separate plasma from erythrocytes, with both fractions kept on ice. Erythrocytes were stored in RNAlater (Sigma-Aldrich, St. Louis, MO, USA cat. no: R0901) at a 1:10 ratio (erythrocytes:RNAlater) and kept at −20 °C for subsequent analysis. In both experiments, the sample size was kept the same:

- Experiment 1: Approximately 200 µL blood was collected from 384 individuals (16 fish per family × 6 families × 2 sampling points (D15 and D30) × 2 diets (FM and PP diet)).
- Experiment 2: Approximately 200 µL blood was collected from 96 individuals (16 fish per family × 3 families × 1 sampling point (D20) × 2 diets (FM and PP diet)).

Considering mortalities during the experimental period, only surviving fish at the end of each trial were used for further analysis.

### 2.4. RNA Extraction

Total RNA was extracted from erythrocytes using the E.Z.N.A.^®^ Total RNA Kit I (OMEGA bio-tek, Norcross, GA, USA, Cat. No: R6834-02), following the manufacturer’s instructions. Briefly, 100 µL of erythrocytes preserved in RNAlater were centrifuged to remove the storage solution and subsequently lysed using a bead beater (Precellys 24 tissue homogenizer, Bertin Technologies, Montigny-le-Bretonneux, France). To eliminate residual genomic DNA, an additional DNase treatment was performed using the DNA-free™ DNA Removal Kit (Invitrogen, Waltham, MA, USA, Cat. No: AM1906), with 1 µL of DNase enzyme added per sample. RNA integrity was assessed by gel electrophoresis, and concentrations were measured using a microvolume spectrophotometer (Quawell Q3000, Quawell Technology, Inc., San Jose, CA, USA). Extracted RNA was stored at −80 °C until further use. The RNA samples were divided into duplicates; one was sent to a genomic center for RNA sequencing, while the other was used for cDNA synthesis. For the cDNA synthesis, 1 μg of total RNA was reverse transcribed using the High-Capacity cDNA Reverse Transcription Kit with RNase Inhibitor (Applied Biosystems™, Waltham, MA, USA, cat no: 4374966) according to the manufacturer’s protocol [13].

### 2.5. RNA Sequencing and Bioinformatics Analysis

Total RNA was pooled equimolarly and sent to Novogene Europe for sequencing.

- Experiment 1: A total of 24 pooled samples (6 families × 2 sampling points (D15 and D30) × 2 diets (FM and PP diet)) were sequenced.
- Experiment 2: A total of 6 pooled samples (3 families × 1 sampling point (D20) × 2 diets (FM and PP diet)) were sequenced.

The indexed libraries were sequenced on the HiSeq 2000 platform (Illumina Inc., San Diego, CA, USA) using 150 bp paired-end reads. Quality control, assembly, and annotation were performed by Novogene Europe (Cambridge, UK). For the detection of Differentially Expressed Genes (DEGs), raw reads produced from Novogene pipeline were used with edgeR’s exact test since there were no biological replicates [14,15,16]. DEGs were identified using a cutoff threshold of a log2FoldChange greater than 0.6 and a *p*-value less than 0.05. Raw transcriptome sequencing data are available in the SRA database under the project ID PRJNA1064006.

Gene enrichment analysis was also performed using ShinyGO (version 0.81, accessed on 13 December 2024), a web-based tool for functional enrichment analysis of gene lists. The differentially expressed genes (DEGs) identified from RNA sequencing were inputted into ShinyGO for pathway enrichment analysis. The analysis was conducted using the default settings for the species *S. aurata* (gilthead seabream), with Gene Ontology (GO) terms, KEGG pathways, and other functional annotations selected for evaluation. A significance threshold of *p* < 0.05 was applied to identify enriched pathways or GO terms. The results were visualized using bar plots and network diagrams to illustrate the most significantly enriched functions and pathways related to the experimental conditions.

### 2.6. Quantitive Expression Analysis (qRT-PCR)

Several differentially expressed genes identified from the RNA-seq analysis were selected for validation. PCR was performed on these genes in the selected families to assess their relative expression levels. Primers were designed using the PrimerBlast tool (NCBI, https://www.ncbi.nlm.nih.gov/tools/primer-blast/, accessed on 3 May 2021) and were evaluated for potential primer–dimer formations, hairpin structures, and other characteristics using Beacon Designer™ Free Edition software (Premier Biosoft, http://www.premierbiosoft.com/qOligo/Oligo.jsp?PID=1, accessed on 5 May 2021).

To assess the efficiency of the reactions, serial dilutions of pooled samples were used. A 10 μL PCR reaction was carried out using the KAPA SYBR^®^ FAST qPCR Master Mix (2×) Kit (Kapa Biosystems, Wilmington, MA, USA, cat no: KK4618), with each gene-specific primer set optimized to the appropriate concentration. The amplification conditions were as follows: an initial step at 95 °C for 5 min, followed by 40 cycles of 95 °C for 20 s and 60 °C for 20 s. A dissociation/melt curve analysis was included to verify the specificity of the primers. Reactions were performed in duplicates, with a maximum ±0.5 difference in Ct values between duplicates applied as the cut-off for acceptability.

A series of genes were evaluated as reference genes for gene expression’ normalization using the RefFinder algorithm (Xie et al., 2012 [19], https://www.ciidirsinaloa.com.mx/RefFinder-master/?type=reference, accessed on 17 October 2025). To calculate the initial fluorescence, the equation R_0_ = T/(E + 1)^Ct^ was used, where T represents a constant threshold for all genes, E denotes the PCR’s efficiency for each gene, and Ct refers to the cycle threshold [20]. Finally, to normalize the relative gene expression, the geometric mean of the most stable genes was used as a normalization factor [21]. The selected genes and their corresponding primers are listed in Table 2.

### 2.7. Statistical Analysis

R packages in RStudio (R version 4.2.0, RStudio version: 2022.12.0) were used for statistical analysis [22,23]. The Shapiro–Wilk test was used to determine the normality of the data [24]. Non-parametric tests were performed due to the deviation of our data from the normal distribution. The Kruskal–Wallis test was applied using the R function Kruskal.test to assess the impact of the two diets on the observed variables, with a significance threshold set at *p* ≤ 0.05 [25]. Graphical representations of the results were generated using the R packages ggplot2 and ggpubr [26,27].

## 3. Results

For the transcriptome analysis in experiment 1, six families out of twenty were selected based on specific SGR patterns observed over the entire duration of the feed experiment in sea cages (10 months). The SGR of the six families selected differentiated between diets as follows: F05 and F17 exhibited differences in SGR from September 2018 to January 2019; F08 and F20 displayed SGR differences only from September to November 2018; F06 and F15 exhibited no differences in SGR between the two dietary treatments. Families from both extreme phenotypes were included in the transcriptome analysis (Table 3). More details on the zootechnical parameters of all twenty families can be found in [13]. In experiment 2, three families were chosen for RNA sequencing using a selection strategy based on SGR. One family was selected because it had the highest final weight on both diets (FM: 484.48 g, PP: 460.58 g) combined with a small coefficient of variation (CV_FM_: 11%, CV_PP_: 15%). The second family was chosen due to the largest weight difference observed between diets (FM: 465.24 g, PP: 346.45 g), and the third family was selected because it had very low final weight on both diets (FM: 329.45 g, PP: 294.95 g) combined with a high CV (CV_FM_: 32%, CV_PP_: 20%).

### 3.1. Experiment 1

#### 3.1.1. Differential Expression Analysis

**Figure 2:**
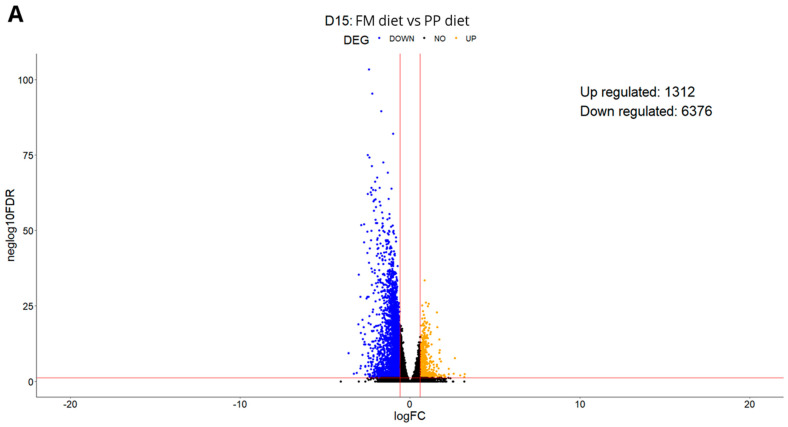

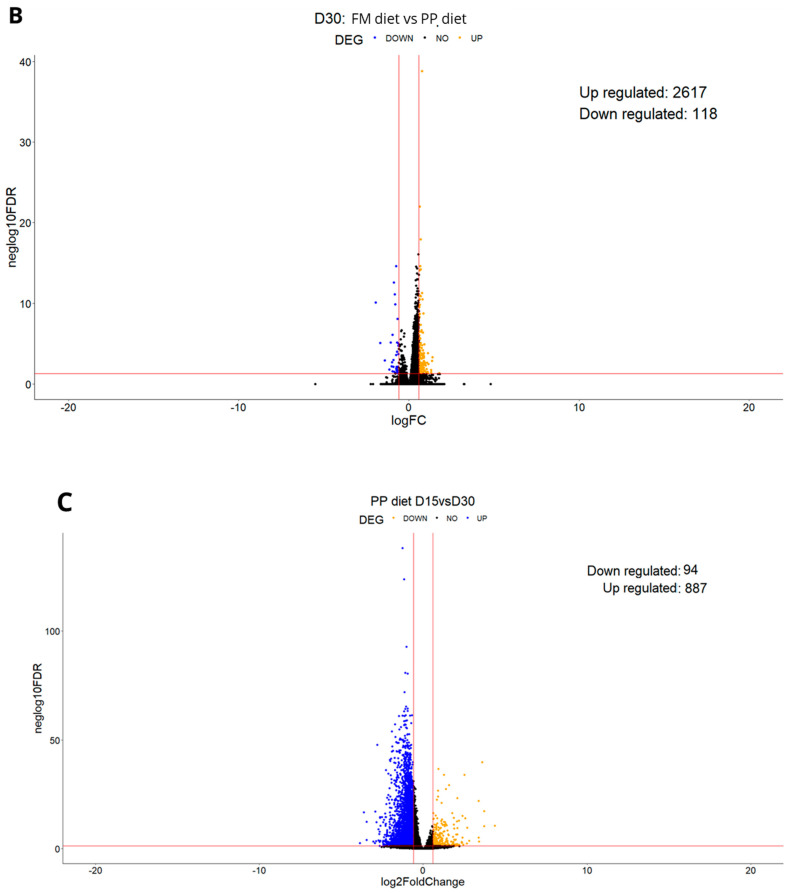

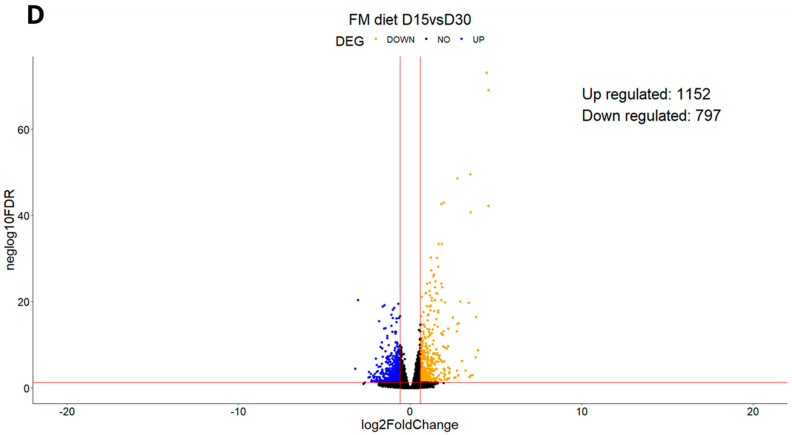
Volcano plots showing differential gene expression across dietary groups and sampling days. (**A**): D15 (FM vs. PP diet), (**B**): D30 (FM vs. PP diet), (**C**): PP diet (D15 vs. D30), (**D**): FM diet (D15 vs. D30). In (**A**,**B**), genes significantly differentially expressed between the PP and FM diets (*p* < 0.05) are shown in blue (downregulated in PP diet) and orange (upregulated in PP diet); black indicates non-significant genes. In (**C**,**D**), significant differences between time points (D15 vs. D30) within each diet are shown in blue (downregulated at D30) and orange (upregulated at D30); black indicates non-significant genes.

A large number of genes were significantly upregulated or downregulated according to the differential expression analysis. On D15, 6376 genes were downregulated in all families in the PP diet compared to the FM diet group. On D30, 2617 genes were downregulated between the two diets (Figure 2A,B). Differential expression analysis between sampling days revealed marked transcriptional changes for both diets. In fish fed the PP diet, 6094 genes were significantly downregulated at D30 compared to D15 across all families. In contrast, fish on the FM diet exhibited 797 genes downregulated and 1152 genes upregulated between the two sampling points (Figure 2C,D). Notably, most families exhibited similar transcriptional patterns across both dietary treatments and sampling days, underscoring the conserved molecular response to the dietary shift as opposed to the small contribution of genetic background.

Differential expression analysis was also performed within each family on each sampling day. Many genes were up- and downregulated, and a volcano plot was created for each comparison (Supplementary Figures S1–S24). An example of the comparisons tested is given in the volcano plots in Figure 3. On both sampling days, upregulated transcription of genes participating in biological processes such as oxidative phosphorylation, metabolic processes, ribosomal proteins, and proteolytic pathways was recorded. On the contrary, downregulation was observed in genes implicated in phospholipid synthesis and the membrane cytoskeleton.

#### 3.1.2. KEGG Enrichment Analysis

**Figure 4:**
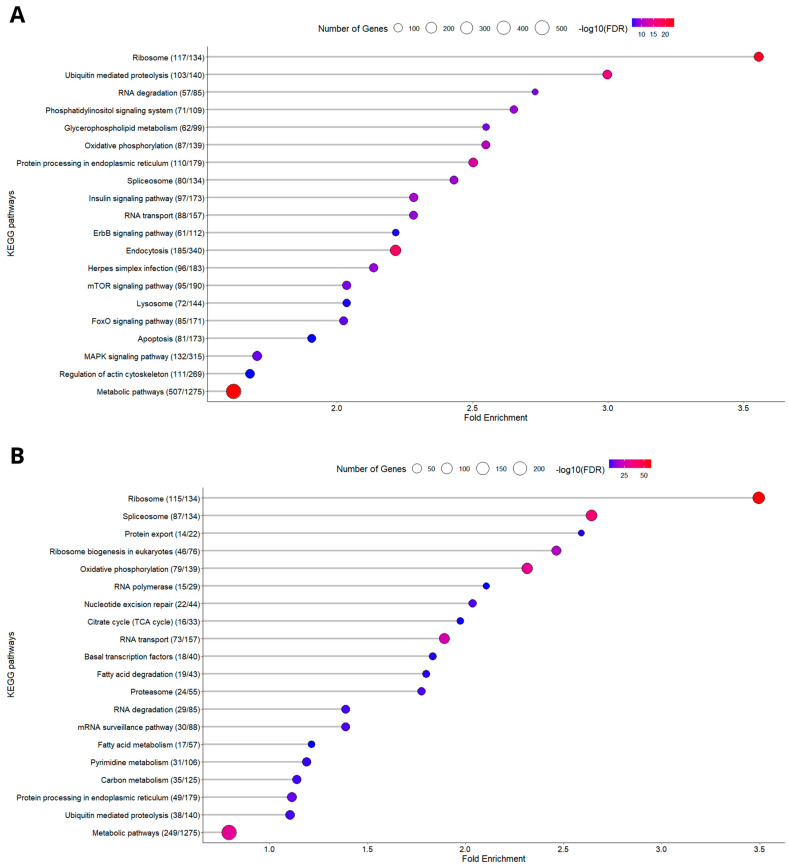
KEGG pathway analysis on different sampling days between the two different nutritional conditions. (**A**) D15, (**B**) D30. The *x*-axis represents the fold enrichment, showing the degree to which each pathway is overrepresented in the dataset compared to the background. The *y*-axis lists the enriched KEGG pathways, ordered by fold enrichment. The size of the circles reflects the number of differentially expressed proteins mapped to each pathway, with larger circles representing pathways involving more proteins. The color gradient of the circles, ranging from blue to red, represents the significance of enrichment, measured by the −log10(FDR) value, where red indicates higher significance.

**Figure 5:**
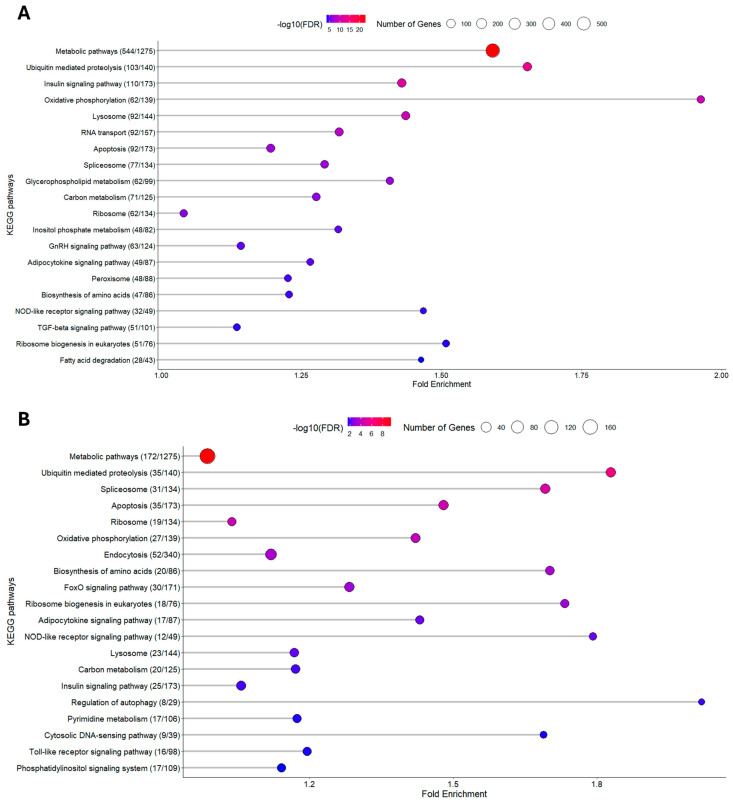
KEGG pathway analysis on different feeds between the two different sampling days. (**A**) PP diet, (**B**) FM diet. The *x*-axis represents the fold enrichment, showing the degree to which each pathway is overrepresented in the dataset compared to the background. The *y*-axis lists the enriched KEGG pathways, ordered by fold enrichment. The size of the circles reflects the number of differentially expressed proteins mapped to each pathway, with larger circles representing pathways involving more proteins. The color gradient of the circles, ranging from blue to red, represents the significance of enrichment, measured by the −log10(FDR) value, where red indicates higher significance.

An enrichment analysis was performed using the DEGs identified between the two dietary treatments, across the two sampling points, and among the different families. This analysis aimed to provide a comprehensive understanding of the physiological differences associated with dietary and temporal variations, as well as family-specific responses. KEGG pathways were selected to illustrate the broader biological processes affected, offering insights into how key metabolic and cellular functions of red blood cells (RBCs) are influenced by plant-based diets in gilthead seabream. The highest number of DEGs identified clustered within the KEGG pathways “Metabolic pathways”, “Ribosome”, and “Oxidative phosphorylation” (Figure 4 and Figure 5). This uniform enrichment across families was another indication of a conserved molecular response to the dietary shift, underscoring the key role of cellular energy production, protein synthesis, and overall metabolic regulation in response to plant-based diets.

### 3.2. Experiment 2

#### 3.2.1. Differential Expression Analysis

To verify whether the observed transcriptional responses were consistent and not incidental, the nutritional experiment was repeated using new full-sib families generated a year later in 2019 by AVRAMAR SA. In an attempt to establish a single time-point to capture responses following a dietary shift, D20 was chosen in this experiment, falling between the sampling days used in experiment 1.

**Figure 6:**
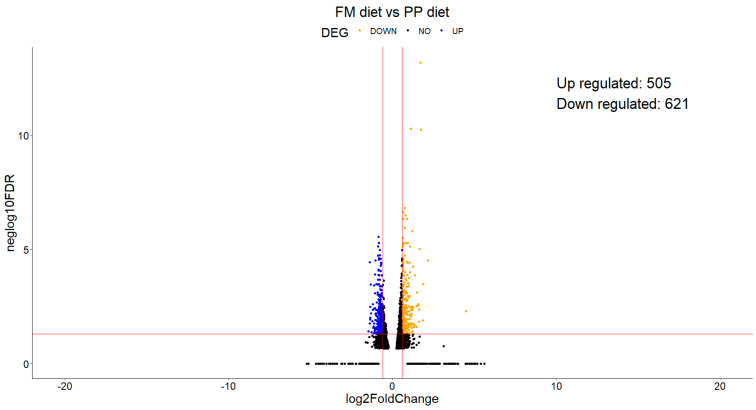
Volcano plots depicting genes upregulated in both dietary groups on D20. Statistically significant (*p* value < 0.05) over/under expression is depicted in blue (downregulated in PP diet), orange (upregulated in PP diet), whereas black shows non-statistically significant regulated genes.

Differential expression analysis revealed a significant number of genes that were either upregulated or downregulated. On the sampling day (D20), 621 genes were downregulated, while 504 genes were upregulated in fish fed the PP diet compared to those fed the FM diet (Figure 6).

#### 3.2.2. KEGG Enrichment Analysis

**Figure 7:**
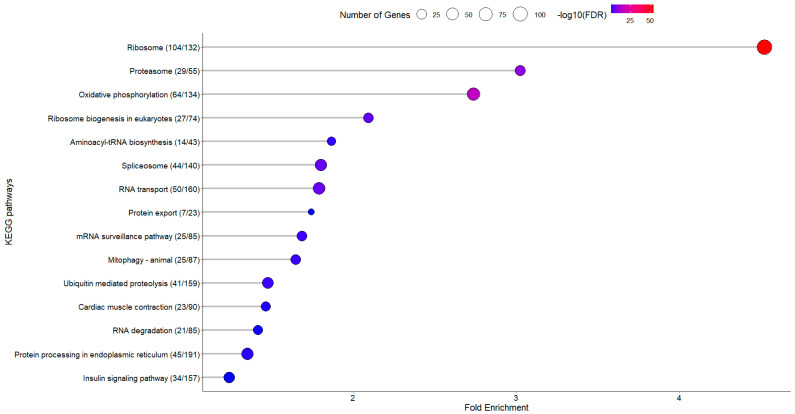
KEGG pathway analysis on sampling day D20 between the FM and PP diet. The *x*-axis represents the fold enrichment, showing the degree to which each pathway is overrepresented in the dataset compared to the background. The *y*-axis lists the enriched KEGG pathways, ordered by fold enrichment. The size of the circles reflects the number of differentially expressed proteins mapped to each pathway, with larger circles representing pathways involving more proteins. The color gradient of the circles, ranging from blue to red, represents the significance of enrichment, measured by the −log10(FDR) value, where red indicates higher significance.

Enrichment analysis revealed a consistent downregulation of key pathways, including Oxidative Phosphorylation, Ribosome, and Metabolic Processes, in fish fed the PP diet, aligning with the findings from experiment 1. The reproducible downregulation of these pathways suggests a systematic physiological adjustment to the PP diet, potentially reflecting reduced energy expenditure on protein synthesis and turnover. This adaptation may serve to maintain cellular homeostasis by modulating metabolic demands in response to the altered nutrient composition of the plant-based diet (Figure 7).

### 3.3. Oxidative Phosphorylation (OxPhos) Pathway

**Figure 8:**
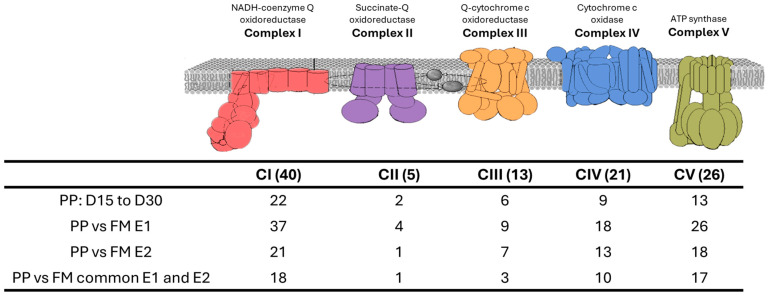
The figure depicts the common DEGs participating in each OxPhos complex. In parenthesis, the total number of genes in each complex is given. OxPhos illustration was adapted by https://www.kegg.jp/entry/map00190 (accessed on 22 February 2025).

The analysis of DEGs associated with the oxidative phosphorylation (OxPhos) pathway revealed significant differences across the investigated conditions. Several genes encoding key components of the electron transport chain were differentially expressed, highlighting potential metabolic adaptations to the plant-protein (PP) diet. A comparison between the two experiments demonstrated a substantial overlap in DEGs across different time points (D15, D30, and D20). Notably, experiment 2 shared a subset of DEGs with experiment 1, indicating a conserved transcriptional response across families of different genetic background (Figure 8, Supplementary Table S1).

### 3.4. Ribosome Pathway

**Figure 9:**
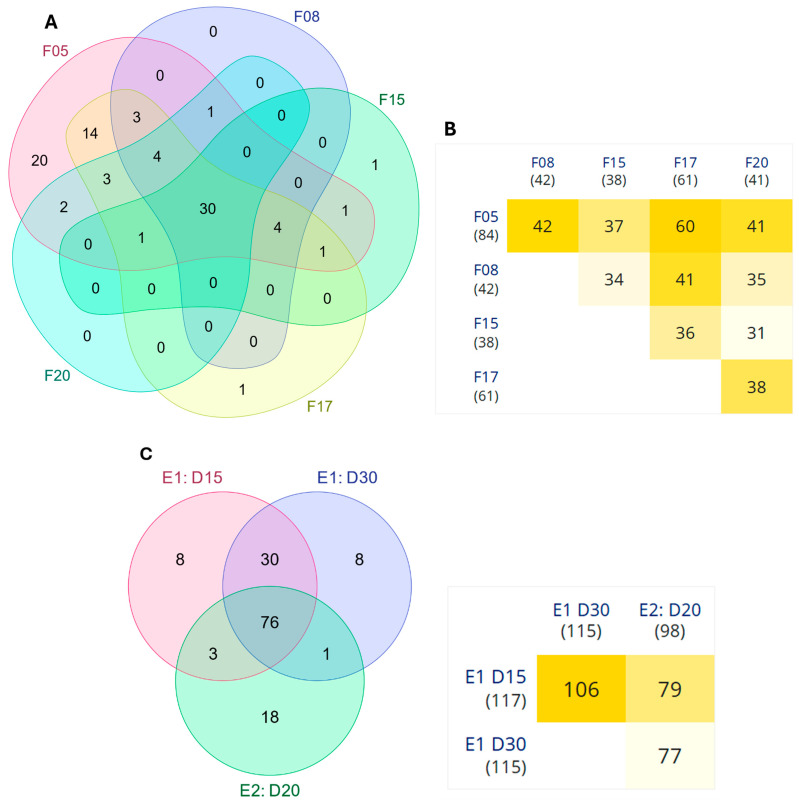
Comparison of DEGs related to the “Ribosome” pathway across five full-sib families (F05, F08, F15, F17, and F20) of gilthead seabream (*S. aurata*) fed a plant-protein (PP) diet. (**A**) Venn diagram illustrating the overlap of ribosome-related DEGs among five families at two sampling points (D15 vs. D30 days) in experiment 1. The central intersection shows 30 DEGs shared across all families. Unique and shared gene counts for each family comparison are also presented. (**B**) Heatmap showing pairwise overlaps in the number of ribosome-related DEGs between families in experiment 1. The intensity of the yellow color corresponds to the number of shared genes, with exact values indicated within each cell. The total number of DEGs per family comparison is shown in parentheses next to each family label. (**C**) Venn diagram and heatmap illustrating the overlap of DEGs between the two experiments (Y1 and Y2) at different sampling points (D15, D30, and D20) for PP vs. FM diet comparisons. The central intersection shows 76 DEGs shared across all comparisons. The heatmap displays pairwise overlaps, with the intensity of yellow shading reflecting the number of shared genes.

The ribosome pathway analysis identified numerous DEGs across five (F05, F08, F15, F17, and F20) of the six full-sib families of gilthead seabream (*S. aurata*) fed with the PP diet. A Venn diagram comparing the DEGs at two time points (D15 and D30) revealed that 30 DEGs were shared across all five families (Figure 9A, Supplementary Table S2). This core set of genes encodes for ribosomal proteins, indicating a possible impact of the PP diet on ribosome number (Figure 9A). In addition, a heatmap of pairwise overlaps in ribosome-related DEGs between families revealed varying degrees of shared gene expression patterns, with some families displaying higher overlap than others (Figure 9B). When focusing on comparisons between experiment 1 and experiment 2 at different sampling points, a total of 76 genes were consistently differentially expressed, reinforcing the importance of ribosomal regulation in dietary adaptation (Figure 9C, Supplementary Table S3).

### 3.5. Metabolic Pathways

**Figure 10:**
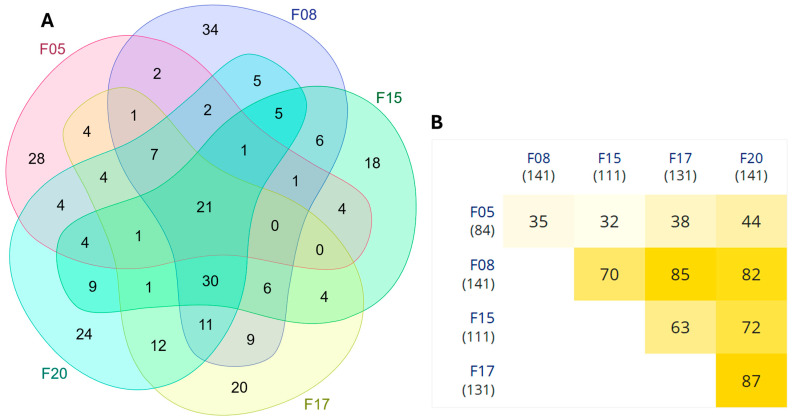
Comparison of differentially expressed genes (DEGs) related to the “Metabolic pathways” pathway across five full-sib families (F05, F08, F15, F17, and F20) of gilthead seabream (*S. aurata*) fed a plant-protein (PP) diet in experiment 1. (**A**) Venn diagram illustrating the overlap of metabolic pathways-related DEGs at two sampling points (D15 vs. D30 days). The central intersection shows 21 DEGs shared across the five families. Unique and shared gene counts for each family comparison are also presented. (**B**) Heatmap showing pairwise overlaps in the number of metabolic pathways-related DEGs between families. The intensity of the yellow color corresponds to the number of shared DEGs, with exact values indicated within each cell. The total number of DEGs per family comparison is shown in parentheses next to each family label.

A substantial number of DEGs across five of the six full-sib families were clustered within “Metabolic pathways”, suggesting widespread metabolic reprogramming due to the PP diet. The Venn diagram analysis showed that 21 DEGs were shared between the five families, indicating common metabolic adjustments across genetic backgrounds (Supplementary Table S4). The heatmap analysis further highlighted the extent of overlap between families, with some exhibiting stronger transcriptional similarities. These results suggest that, while individual families may respond differently to dietary changes, a core set of metabolic genes consistently responds to the PP diet, contributing to energy metabolism, amino acid biosynthesis, and lipid metabolism (Figure 10).

### 3.6. Differential Gene Expression Validation

**Figure 11:**
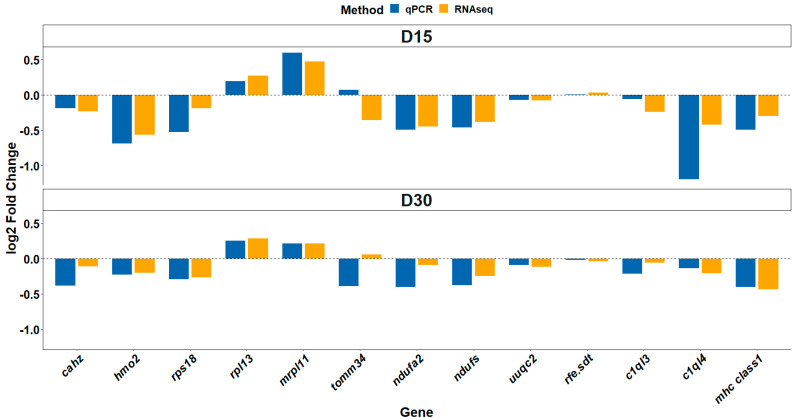
The figure presents the log2foldchange in expression of selected validation genes used to confirm RNAseq results through qPCR. Expression levels were compared between fish fed the PP diet and those fed the FM diet at two time-points (D15 and D30). Orange bars represent RNAseq data, while blue bars indicate qPCR results. The dashed horizontal line denotes no expression change. Positive values indicate upregulation and negative values downregulation in the PP diet group. The figure illustrates the consistency between qPCR and RNAseq findings across genes and sampling points.

To ensure accurate normalization, we identified the most stably expressed genes across all samples as candidate reference genes before selecting *ef1a* and *hbba2* as the actual reference genes. The final normalization strategy was adjusted accordingly to minimize bias in gene expression quantification (Table 1). The qPCR analysis of 13 selected genes (*cahz*, *hmo2*, *rps18*, *rpl13*, *mrpl11*, *tomm34*, *ndufa2*, *ndufs*, *uuqc2*, *rfe.sdt*, *c1ql3*, *c1ql4*, and *mhc class1*) revealed a significant positive correlation with the normalized RNA-seq read counts (Figure 11). Consistent expression patterns, including both upregulation and downregulation, were observed across all comparisons, which aligned with the qPCR amplification results for these candidate genes.

## 4. Discussion

The existing scientific literature demonstrates significant efforts to improve growth rates and adaptation to dietary changes in aquaculture while minimizing adverse effects on overall growth and health [28]. However, a key limitation of current practices is that traits such as growth performance and dietary adaptability are typically assessed only after harvest, necessitating lengthy rearing periods before evaluation [5].

To address this challenge, there is a growing interest in utilizing peripheral blood as a non-invasive and easily accessible resource for monitoring physiological changes. This approach aligns with recent trends in animal farming, where blood analysis serves as a valuable tool for assessing various physiological states, enabling real-time evaluation and potentially reducing reliance on post-harvest assessments [18]. By leveraging this methodology, aquaculture systems can introduce more efficient and timely decision-making processes, ultimately supporting sustainable and optimized fish farming practices.

In our study, the two feeds were composed of similar percentages of protein and lipids, although the trial feed contained a much higher percentage of plant proteins and plant oils. Plant protein sources, while less expensive and more readily available, may include anti-nutritional factors and can induce various physiological responses [9,29]. Our study demonstrates for the first time that shifting to a plant-rich diet affects the transcriptome of the RBCs, and this effect takes place within the first weeks of the dietary shift. In two sequential experiments performed one year apart using a commercial setting and practices with varied genetic material, the dietary shift evoked consistent changes in red blood transcriptome. Transcriptome analysis revealed that the most affected KEGG pathways (i.e., enriched with DEGs) were associated with the ribosome, oxidative phosphorylation, and metabolic pathways. The transcriptomic responses observed were largely consistent across families, suggesting that the effects of the plant-based diet on key biological processes—such as oxidative phosphorylation, ribosomal activity, and metabolic regulation—are independent of genetic background. This uniform response implies a common physiological adaptation to dietary intervention, highlighting the dominant influence of diet over inherent family-specific variation in shaping red blood cell gene expression profiles.

Erythrocytes play a crucial role in oxygen transport, acid–base balance, and metabolic homeostasis in fish, adapting to environmental and physiological challenges. Their primary function is oxygen delivery, facilitated by hemoglobin (Hb) genes, which encode proteins essential for oxygen binding and release. Changes in hemoglobin gene expression can influence oxygen-carrying capacity, affecting metabolic efficiency under different dietary conditions [30]. Carbonic anhydrases (CAs) contribute to acid–base regulation by catalyzing the reversible conversion of CO_2_ and water into bicarbonate and protons, a key process for maintaining pH homeostasis and facilitating CO_2_ excretion [31]. Additionally, membrane transporters, including ion pumps and channels, regulate intracellular ion balance and gas exchange, ensuring erythrocytes maintain their structural integrity and function under varying physiological demands [32].

The impact of dietary composition on erythrocyte physiology remains an understudied area, yet our findings indicate that erythrocyte transcriptomic responses reflect metabolic and structural adjustments to plant-based diets. In our study, key erythrocyte-associated genes, including carbonic anhydrase 5A (*ca5a*) and band 3 anion exchange protein-like (*slc4a1*), were downregulated in fish fed with the PP diet, with more pronounced effects observed at D15 compared to D30, suggesting an early phase of cellular adaptation (Supplementary Table S5). Carbonic anhydrase plays a fundamental role in acid–base balance and CO_2_ excretion, while band 3 anion exchangers are key to normal erythrocyte function [31,33]. Their downregulation could indicate a temporary disruption in gas exchange efficiency and ionic regulation, potentially driven by nutritional differences between fishmeal- and plant-based feeds. Studies on red drum (*Sciaenops ocellatus*) have shown that different feeding regimes significantly influence hematological parameters and erythrocyte membrane composition, supporting the idea that dietary changes can alter erythrocyte function beyond cell numbers [34]. The initial downregulation of these genes at D15, followed by partial recovery at D30, suggests that fish underwent a period of physiological adjustment, progressively stabilizing their erythrocyte profile over time. This response could be attributed to transcriptional regulation, post-translational modifications, or compensatory mechanisms within the RBC membrane. Given that plant-based diets differ from fishmeal in amino acid composition, mineral content, and bioactive compounds, the observed erythrocyte transcriptomic shifts may reflect systemic metabolic adaptations to maintain homeostasis under altered dietary conditions.

### 4.1. Oxidative Phosphorylation

The oxidative phosphorylation (OxPhos) pathway, responsible for 90% of the energy production in aerobic organisms, is highly sensitive to metabolic and environmental changes, including dietary shifts. The analysis of DEGs in the OxPhos pathway revealed significant alterations across all complexes of the electron transport chain (CI-CV). A large proportion of genes associated with these complexes were differentially expressed, indicating a broad transcriptional response of gilthead seabream to the PP diet. Moreover, the overlap in DEGs between two experimental years (Y1 and Y2) across different time points (D15, D30, and D20) highlights the robustness and reproducibility of the transcriptional response to the PP diet. Despite potential environmental and temporal variations, a conserved set of genes was upregulated, particularly those involved in electron transport and ATP synthesis [35].

The downregulation of OxPhos genes observed in our study suggests that the plant-protein (PP) diet may induce metabolic stress that compromises mitochondrial efficiency in erythrocytes, similar to findings from studies conducted in other tissues. Silva-Marrero et al. [36] observed this effect in gilthead seabream using both white muscle and liver, while Torricelli et al. [37] reported it in liver tissue of gilthead seabream, and Vera et al. [38] found similar patterns in liver tissue of salmon. While some studies have reported upregulation of OxPhos in response to plant-based diets, our findings align with others where downregulation occurs. This discrepancy could be due to the presence of anti-nutritional factors in plant-based feeds, which can impair mitochondrial function. The downregulation in our study may reflect a compensatory response to balance energy production with the stress induced by the PP diet, emphasizing the need for efficient energy regulation under these feeding conditions.

The temporal analysis of OxPhos gene expression also provides insight into the adaptation process over time. Comparing gene expression between different time points (D15 and D30) revealed that the transcriptional response to the PP diet evolves as the fish acclimates to the plant-based feed. The consistent downregulation of some OxPhos genes at later time points may reflect a metabolic shift toward stress adaptation rather than growth, further suggesting that the PP diet imposes trade-offs between energy production, growth, and stress management.

### 4.2. Ribosomal KEGG Pathway

The dietary shift particularly affected ribosomal KEGG pathways, a cornerstone of protein synthesis and cellular homeostasis, with 72 DEGs in a total of 92 expressed ribosomal genes identified across five full-sib families, reflecting differential translational capacity in response to dietary changes. Notably, family F06 exhibited no significant DEGs, suggesting minimal transcriptional response to the plant-protein (PP) diet, while the other families displayed varying degrees of ribosomal capacity alteration.

Specifically, 37 out of 39 genes associated with the large ribosomal subunit and 22 out of 24 genes from the small ribosomal subunit were differentially expressed. Additionally, mitochondrial ribosomal components were also impacted, with 8 out of 19 genes from the mitochondrial large ribosomal subunit and 5 out of 9 genes from the mitochondrial small ribosomal subunit exhibiting differential regulation. In addition, a total of 71 genes were consistently differentially expressed between experiments 1 and 2, reinforcing the importance of ribosomal regulation in dietary adaptation and providing grounds for the observed differences in growth and metabolic adjustments between dietary groups. The transcriptional changes observed might suggest that ribosomes pivot from supporting growth to facilitating stress adaptation. This aligns with the broader concept that plant-based diets may drive a reconfiguration of ribosomal activity, balancing growth, immunity, and stress management [39]. The temporal analysis of ribosomal gene expression further supports the notion of an adaptive response to plant-based diets.

A core set of 30 genes was consistently regulated across multiple families and time points, reinforcing the idea that ribosomal machinery plays a crucial role in dietary adaptation [40]. Besides these core genes, the heatmap analysis revealed varying degrees of overlap in ribosome-related DEGs between families, suggesting that genetic background may influence the boundaries of adaptation of *S. aurata* families to plant-based diets.

Proteomic and metabolomic studies provide further context for these results, as they corroborate the transcriptional changes observed in our study. Torricelli et al. [37] conducted their study in the liver and intestine of *S. aurata*, while Xu et al. [41] investigated these effects in the liver of Japanese seabass (*Lateolabrax japonicus*). Previous research has shown that plant-based diets can trigger ribosomal assembly stress, marked by the differential expression of genes involved in ribosomal RNA processing and ribosome biogenesis [39]. For example, stress can lead to the downregulation of insulin-like growth factor 1 (*IGF-1*) and related signaling pathways, suggesting a reduction in anabolic processes, including ribosomal biogenesis [42]. This reduction in ribosome production could impair protein synthesis efficiency, potentially limiting growth and overall performance in European seabass [43] and salmon [44]. Our findings, with the identification of differentially expressed ribosomal genes across families, suggest that these regulatory changes in ribosome production may be part of a broader metabolic reprogramming under the PP diet.

Additionally, metabolic challenges posed by plant-based diets show that nutrient deficiencies, such as the lack of essential amino acids like methionine and lysine, trigger stress responses in fish, including amino acid starvation pathways. As described by Magalhaes et al. [42] the activation of kinases like GCN2, which phosphorylate eukaryotic initiation factor 2α (*eIF2α*) to globally suppress ribosomal initiation, might be a key mechanism in response to these deficiencies. These processes could limit protein synthesis while allowing selective translation of stress-response proteins, such as chaperones and detoxification enzymes [40].

### 4.3. Metabolic Pathways

The substitution of fishmeal with plant-based proteins in aquafeeds induced significant metabolic reprogramming, as evidenced by the differential expression of genes involved in key metabolic pathways. Our analysis of DEGs across the families revealed widespread metabolic adjustments to the PP diet. Notably, a core set of 21 genes was consistently differentially expressed across five families, suggesting that certain metabolic pathways—specifically those involved in energy metabolism, amino acid biosynthesis, and lipid metabolism—were activated by the PP diet. While some families exhibited distinct patterns, the core set of metabolic genes regulating energy production and nutrient utilization was consistently expressed, reinforcing the idea of shared metabolic adaptations to the PP diet. Specifically, we observed changes in the regulation of glycolysis, gluconeogenesis, and lipid metabolism, which are indicative of adaptive responses aimed at optimizing nutrient use under the dietary conditions [45]. Lipid metabolism was particularly impacted, with significant alterations in cholesterol biosynthesis regulation (Supplementary Figures S25 and S26). These changes likely reflect the adaptive response to the inclusion of plant sterols in the diet, which can influence membrane properties [46]. This is consistent with prior research in Atlantic salmon indicating that plant sterols alter membrane fluidity and permeability, necessitating an increase in cholesterol production to maintain membrane integrity and cellular function [47]. Geay et al. [43] reported similar effects in the liver of European seabass, Kemski et al. [48] observed this response in the intestine of yellow perch (*Perca flavescens*), and Krogdahl et al. [49] documented comparable adaptations in the intestine of salmon.

The interplay between oxidative phosphorylation, ribosome function, metabolic pathways, and protein degradation provides a mechanistic explanation for the observed lower growth in fish fed on the PP diet. The interconnected nature of these pathways highlights the complexity of the metabolic adjustments induced by the PP diet. The downregulation of OxPhos reduces energy availability, which in turn limits protein synthesis via the ribosome pathway [50]. This reduced protein synthesis, coupled with increased protein degradation through ubiquitin-mediated proteolysis, creates an imbalance where protein loss surpasses protein accumulation, leading to impaired growth [43,51,52]. Additionally, metabolic reprogramming in response to plant-derived nutrients appears to alter lipid metabolism, which may further constrain growth performance by modifying energy utilization and membrane stability. The downregulation of genes participating in these pathways may indicate that the fish have experienced an energy deficit, forcing a metabolic shift that prioritizes maintenance over growth.

## 5. Conclusions

In summary, our study demonstrates the potential of red blood cell (RBC) transcriptomics as a minimally invasive tool for assessing physiological responses to dietary shifts in gilthead seabream. The consistent transcriptional changes observed across two independent experiments highlight key metabolic and cellular adaptations to plant-protein-based diets, particularly in oxidative phosphorylation, ribosomal activity, and lipid metabolism. These findings underscore the importance of integrating transcriptomic biomarkers into aquaculture breeding and nutrition programs to improve feed efficiency, fish welfare, and sustainability. By providing an early and reliable molecular readout of fish responses to dietary changes, this approach offers valuable insights for optimizing feed formulations and genetic selection strategies, ultimately contributing to a more resilient and environmentally responsible aquaculture industry.

## Figures and Tables

**Figure 3:**
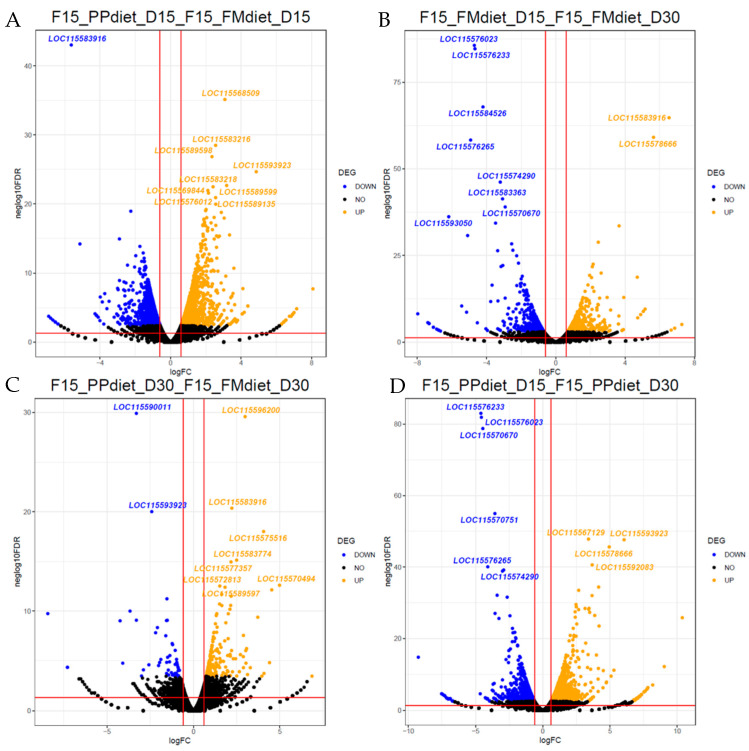
Volcano plots of differentially expressed genes in F15. Comparisons drawn: (**A**) PP vs. FM diets on D15, (**B**) PP vs. FM diets on D30, (**C**) PP diet: D15 vs. D30, and (**D**) FM diet: D15 vs. D30. Statistically significant (*p* value < 0.05) downregulated DEGs in condition 2 are depicted in blue, upregulated DEGs in condition 2 are depicted in orange, and black shows non-statistically significant regulated genes. The name of the top ten up and down DEGs is also given in the plot.

**Table 1:** Formulation of the two diets expressed as percentage of dry weight. Table adapted by [13].

Ingredients	FM Diet	PP Diet
Fish meal standard %	24.6	
Fish meal LT %		8.1
Fish oil %	4.6	5.0
Salmon oil %	6.5	
Rapeseed oil %		6.5
Soya bean meal %	9.9	21.0
Rapeseed meal %		14.0
Soya protein concentrate %	17.0	7.0
Corn gluten %	14.5	9.6
Plant premix * %	6.6	8.0
Sunflower meal %	5.0	4.1
Wheat %	9.6	10.7
Amino acid premix ** %		3.2
Vitamin and mineral premix %	1.0	1.0
Phospholipids *** %	0.3	0.8
Ca & P source **** %	0.4	1.0

* Mix of single cell protein, by-products of amino-acids production and by-products of nGMO cereals’ fermentation. ** L-Lysine 79%, DL-Methionine 99%, L-Threonine 98.5%. *** Hydrolyzed Lecithin source FRA Lecimax. **** Monocalcium phosphate and carbon carbonate.

**Table 2:** Genes selected for validation of differential expression through Real-Time PCR.

Gene ID	Gene Description	Gene Name	Forward Primer	Reverse Primer	Product Size (bp)
ENSSAUG00010018560/XM_030411990.1	elongation factor 1-alpha, somatic form	*ef1a*	TCAAGGGATGGAAGGTTGAG	AGTTCCAATACCGCCGAT	152
ENSSAUG00010007010	hemoglobin, beta adult 2	*hbba2*	GCAAGGGTGCTGATCGTCTA	GGGCTGCCACTTTGGAGTTA	103
ENSSAUG00010003114	ribosomal protein L13a	*rpl13*	TCTGGAGGACTGTCAGGGGCATGC	AGACGCACAATCTTAAGAGCAG	197
ENSSAUG00010000811	40S ribosomal protein S18	*rps18*	AGGGTGTTGGCAGACGTTAC	GAGGACCTGGCTGTATTTGC	148
ENSSAUG00010012992	complement C1q-like protein 3	*c1ql3*	TTTGGAGACGGAGCGAAGAC	CCATACGCCTCACCACCTTT	121
ENSSAUG00010012990	complement C1q-like protein 4	*c1ql4*	AGGTTGACACAGCCTTCCATA	CACTCATGTTGGGTTTGCAGG	111
ENSSAUG00010011851	Carbonic anhydrase	*cahz*	AGGTGGACTTTGTGGACGAC	AAGCTCACAGGGGAACTTGA	155
ENSSAUG00010003856	Rieske (Fe-S) domain containing	*rfe.sdt*	AGATGTGCATCGTTTGTCCA	TAGACATCCCCGTTGGTCTC	166
ENSSAUG00010006080	NADH:ubiquinone oxidoreductase core subunit S1	*ndufs*	CCCACTCTTCAACGCCAGAA	TCCCAGGTGGTCATACGAGT	106
ENSSAUG00010002150	NADH:ubiquinone oxidoreductase subunit A2	*ndufa2*	CAGTAAGGGGGCCAGAGATT	GTTGTCCACCATGACACTGC	157
ENSSAUG00010006606	mitochondrial ribosomal protein L11	*mrpl11*	ACGAGATCGCAAGGGTTAAA	GCTGCTCCAGGAAGATTTTG	159
ENSSAUG00010007642	translocase of outer mitochondrial membrane 34	*tomm34*	CCTGTCGGTGAAGCAGTACA	AGGTTGTTCAGGTCGTCCAC	150
ENSSAUG00010025859	heme oxygenase-like	*hmo2*	CGCCTACACCCGTTATCTGG	GCTGTTCATCCTGCTCCTGT	166
ENSSAUG00010025665	heat shock protein HSP 90-alpha	*hsp90aa*	TGACCCTCAGACACACTCCA	GTCGTCATCGTCCCCTTCAA	139
ENSSAUG00010000727	major histocompatibility complex classI-related gene protein -like	*mhc class 1*	AGATCGGATCGGAACCAACG	CGATGAATCCAACAGCACCG	107

**Table 3:** Zootechnical data throughout the experiment per family (FM diet/PP diet). IBW (Initial body weight), FBW (Final body weight), SGR1 (SGR: September–November), SGR2 (SGR: November–January), SGR3 (SGR: January–March), SGR4 (SGR: March–July), SGR5 (SGR: July–August), Final SGR (SGR: September–August) and Survival rate.

Families	IBW (g)	FBW (g)	SGR1 (%)	SGR2 (%)	SGR3 (%)	SGR4 (%)	SGR5 (%)	Final SGR (%)	Survival Rate (%)
F05	50/46	449/343	0.54/0.46	0.14/0.14	0.11/0.09	0.24/0.21	0.14/0.15	1.77/1.58	87/86
F06	56/54	556/512	0.55/0.51	0.17/0.18	0.1/0.11	0.27/0.27	0.13/0.12	1.85/1.81	89/86
F08	54/51	533/436	0.53/0.4	0.16/0.16	0.1/0.12	0.29/0.31	0.14/0.15	1.79/1.66	87/80
F15	53/49	439/343	0.51/0.44	0.15/0.12	0.1/0.08	0.22/0.24	0.14/0.14	1.75/1.62	85/78
F17	52/43	423/320	0.52/0.46	0.14/0.12	0.08/0.08	0.23/0.24	0.14/0.15	1.73/1.6	88/85
F20	37/34	479/407	0.63/0.52	0.17/0.22	0.13/0.11	0.3/0.35	0.19/0.15	2.03/1.99	91/92

## Data Availability

Transcriptome sequencing data used in this study are available through SRA (BioProject ID PRJNA1064006).

## Supplementary Materials

The following supporting information can be downloaded at https://www.mdpi.com/article/10.3390/ani15091279/s1, Figure S1: Volcano plots of differentially expressed genes in F05. Condition tested: PPvsFM diets on D15. Statistically significant (*p* value < 0.05) down regulated DEGS in condition 2 are depicted in blue, up regulated DEGS in condition 2 are depicted in orange, and black shows non statistically significant regulated genes. The name of the top ten up and down DEGs is also given in the plot; Figure S2: Volcano plots of differentially expressed genes in F05. Condition tested: PPvsFM diets on D30. Statistically significant (*p* value < 0.05) down regulated DEGS in condition 2 are depicted in blue, up regulated DEGS in condition 2 are depicted in orange, and black shows non statistically significant regulated genes. The name of the top ten up and down DEGs is also given in the plot; Figure S3: Volcano plots of differentially expressed genes in F05. Condition tested: FM diet: D15 vs. D30. Statistically significant (*p* value < 0.05) down regulated DEGS in condition 2 are depicted in blue, up regulated DEGS in condition 2 are depicted in orange, and black shows non statistically significant regulated genes. The name of the top ten up and down DEGs is also given in the plot; Figure S4: Volcano plots of differentially expressed genes in F05. Condition tested: PP diet: D15 vs. D30. Statistically significant (*p* value < 0.05) down regulated DEGS in condition 2 are depicted in blue, up regulated DEGS in condition 2 are depicted in orange, and black shows non statistically significant regulated genes. The name of the top ten up and down DEGs is also given in the plot; Figure S5: Volcano plots of differentially expressed genes in F06. Condition tested: PPvsFM diets on D15. Statistically significant (*p* value < 0.05) down regulated DEGS in condition 2 are depicted in blue, up regulated DEGS in condition 2 are depicted in orange, and black shows non statistically significant regulated genes. The name of the top ten up and down DEGs is also given in the plot; Figure S6: Volcano plots of differentially expressed genes in F06. Condition tested: PPvsFM diets on D30. Statistically significant (*p* value < 0.05) down regulated DEGS in condition 2 are depicted in blue, up regulated DEGS in condition 2 are depicted in orange, and black shows non statistically significant regulated genes. The name of the top ten up and down DEGs is also given in the plot; Figure S7: Volcano plots of differentially expressed genes in F06. Condition tested: FM diet: D15 vs. D30. Statistically significant (*p* value < 0.05) down regulated DEGS in condition 2 are depicted in blue, up regulated DEGS in condition 2 are depicted in orange, and black shows non statistically significant regulated genes. The name of the top ten up and down DEGs is also given in the plot; Figure S8: Volcano plots of differentially expressed genes in F06. Condition tested: PP diet: D15 vs. D30. Statistically significant (*p* value < 0.05) down regulated DEGS in condition 2 are depicted in blue, up regulated DEGS in condition 2 are depicted in orange, and black shows non statistically significant regulated genes. The name of the top ten up and down DEGs is also given in the plot; Figure S9: Volcano plots of differentially expressed genes in F08. Condition tested: PPvsFM diets on D15. Statistically significant (*p* value < 0.05) down regulated DEGS in condition 2 are depicted in blue, up regulated DEGS in condition 2 are depicted in orange, and black shows non statistically significant regulated genes. The name of the top ten up and down DEGs is also given in the plot; Figure S10: Volcano plots of differentially expressed genes in F08. Condition tested: PPvsFM diets on D30. Statistically significant (*p* value < 0.05) down regulated DEGS in condition 2 are depicted in blue, up regulated DEGS in condition 2 are depicted in orange, and black shows non statistically significant regulated genes. The name of the top ten up and down DEGs is also given in the plot; Figure S11: Volcano plots of differentially expressed genes in F08. Condition tested: FM diet: D15 vs. D30. Statistically significant (*p* value < 0.05) down regulated DEGS in condition 2 are depicted in blue, up regulated DEGS in condition 2 are depicted in orange, and black shows non statistically significant regulated genes. The name of the top ten up and down DEGs is also given in the plot; Figure S12: Volcano plots of differentially expressed genes in F08. Condition tested: PP diet: D15 vs. D30. Statistically significant (*p* value < 0.05) down regulated DEGS in condition 2 are depicted in blue, up regulated DEGS in condition 2 are depicted in orange, and black shows non statistically significant regulated genes. The name of the top ten up and down DEGs is also given in the plot; Figure S13: Volcano plots of differentially expressed genes in F15. Condition tested: PPvsFM diets on D15. Statistically significant (*p* value < 0.05) down regulated DEGS in condition 2 are depicted in blue, up regulated DEGS in condition 2 are depicted in orange, and black shows non statistically significant regulated genes. The name of the top ten up and down DEGs is also given in the plot; Figure S14: Volcano plots of differentially expressed genes in F15. Condition tested: PPvsFM diets on D30. Statistically significant (*p* value < 0.05) down regulated DEGS in condition 2 are depicted in blue, up regulated DEGS in condition 2 are depicted in orange, and black shows non statistically significant regulated genes. The name of the top ten up and down DEGs is also given in the plot; Figure S15: Volcano plots of differentially expressed genes in F15. Condition tested: FM diet: D15 vs. D30. Statistically significant (*p* value < 0.05) down regulated DEGS in condition 2 are depicted in blue, up regulated DEGS in condition 2 are depicted in orange, and black shows non statistically significant regulated genes. The name of the top ten up and down DEGs is also given in the plot; Figure S16: Volcano plots of differentially expressed genes in F15. Condition tested: PP diet: D15 vs. D30. Statistically significant (*p* value < 0.05) down regulated DEGS in condition 2 are depicted in blue, up regulated DEGS in condition 2 are depicted in orange, and black shows non statistically significant regulated genes. The name of the top ten up and down DEGs is also given in the plot; Figure S17: Volcano plots of differentially expressed genes in F17. Condition tested: PPvsFM diets on D15. Statistically significant (*p* value < 0.05) down regulated DEGS in condition 2 are depicted in blue, up regulated DEGS in condition 2 are depicted in orange, and black shows non statistically significant regulated genes. The name of the top ten up and down DEGs is also given in the plot; Figure S18: Volcano plots of differentially expressed genes in F17. Condition tested: PPvsFM diets on D30. Statistically significant (*p* value < 0.05) down regulated DEGS in condition 2 are depicted in blue, up regulated DEGS in condition 2 are depicted in orange, and black shows non statistically significant regulated genes. The name of the top ten up and down DEGs is also given in the plot; Figure S19: Volcano plots of differentially expressed genes in F17. Condition tested: FM diet: D15 vs. D30. Statistically significant (*p* value < 0.05) down regulated DEGS in condition 2 are depicted in blue, up regulated DEGS in condition 2 are depicted in orange, and black shows non statistically significant regulated genes. The name of the top ten up and down DEGs is also given in the plot; Figure S20: Volcano plots of differentially expressed genes in F17. Condition tested: PP diet: D15 vs. D30. Statistically significant (*p* value < 0.05) down regulated DEGS in condition 2 are depicted in blue, up regulated DEGS in condition 2 are depicted in orange, and black shows non statistically significant regulated genes. The name of the top ten up and down DEGs is also given in the plot; Figure S21: Volcano plots of differentially expressed genes in F20. Condition tested: PPvsFM diets on D15. Statistically significant (*p* value < 0.05) down regulated DEGS in condition 2 are depicted in blue, up regulated DEGS in condition 2 are depicted in orange, and black shows non statistically significant regulated genes. The name of the top ten up and down DEGs is also given in the plot; Figure S22: Volcano plots of differentially expressed genes in F20. Condition tested: PPvsFM diets on D30. Statistically significant (*p* value < 0.05) down regulated DEGS in condition 2 are depicted in blue, up regulated DEGS in condition 2 are depicted in orange, and black shows non statistically significant regulated genes. The name of the top ten up and down DEGs is also given in the plot; Figure S23: Volcano plots of differentially expressed genes in F20. Condition tested: FM diet: D15 vs. D30. Statistically significant (*p* value < 0.05) down regulated DEGS in condition 2 are depicted in blue, up regulated DEGS in condition 2 are depicted in orange, and black shows non statistically significant regulated genes. The name of the top ten up and down DEGs is also given in the plot; Figure S24: Volcano plots of differentially expressed genes in F20. Condition tested: PP diet: D15 vs. D30. Statistically significant (*p* value < 0.05) down regulated DEGS in condition 2 are depicted in blue, up regulated DEGS in condition 2 are depicted in orange, and black shows non statistically significant regulated genes. The name of the top ten up and down DEGs is also given in the plot; Figure S25: Go term enrichment analysis of differentially expressed proteins in the three GO annotation domains: Biological Processes (BP), Cellular Components (CC), and Molecular Functions (MF) E1: A: FM vs. PP D15, B: FM vs. PP D30. The x-axis represents the fold enrichment, indicating the magnitude of overrepresentation of GO terms in the dataset compared to the background. The y-axis lists the enriched GO terms, ordered by fold enrichment within each category. The size of the circles corresponds to the number of differentially expressed proteins associated with each GO term, with larger circles representing GO terms involving more proteins. The color gradient of the circles, ranging from blue to red, reflects the significance of enrichment as measured by the −log10(FDR) value, with red representing more significant enrichment; Figure S26: Go term enrichment analysis of differentially expressed proteins in the three GO annotation domains: Biological Processes (BP), Cellular Components (CC), and Molecular Functions (MF) E2: FM vs. PP. The x-axis represents the fold enrichment, indicating the magnitude of overrepresentation of GO terms in the dataset compared to the background. The y-axis lists the enriched GO terms, ordered by fold enrichment within each category. The size of the circles corresponds to the number of differentially expressed proteins associated with each GO term, with larger circles representing GO terms involving more proteins. The color gradient of the circles, ranging from blue to red, reflects the significance of enrichment as measured by the −log10(FDR) value, with red representing more significant enrichment; Table S1: Common oxidative phosphorylation (OxPhos) genes identified across full-sib families. The table lists OxPhos-related genes that were differentially expressed in response to the plant-protein (PP) diet and indicates the families (F5, F08, F15, F17, and F20) in which each gene was detected; Table S2: Common Ribosome genes identified across full-sib families. The table lists Ribosome-related genes that were differentially expressed in response to the plant-protein (PP) diet and indicates the families (F5, F08, F15, F17, and F20) in which each gene was detected; Table S3: Ribosomal Genes Identified in E1 vs. E2 Comparison. This table lists ribosomal genes identified in the comparison between experimental groups E1 and E2 across different sampling time points; Table S4: Common Metabolic pathways related genes identified across full-sib families. The table lists Metabolic pathways-related genes that were differentially expressed in response to the plant-protein (PP) diet and indicates the families (F5, F08, F15, F17, and F20) in which each gene was detected; Table S5: DEGs for key erythrocyte-associated genes.

## References

[1] Naylor R.L., Hardy R.W., Buschmann A.H., Bush S.R., Cao L., Klinger D.H., Little D.C., Lubchenco J., Shumway S.E., Troell M. (2021). A 20-Year Retrospective Review of Global Aquaculture. Nature.

[2] Egerton S., Wan A., Murphy K., Collins F., Ahern G., Sugrue I., Busca K., Egan F., Muller N., Whooley J. (2020). Replacing Fishmeal with Plant Protein in Atlantic Salmon (*Salmo salar*) Diets by Supplementation with Fish Protein Hydrolysate. Sci. Rep..

[3] Turchini G.M., Trushenski J.T., Glencross B.D. (2019). Thoughts for the Future of Aquaculture Nutrition: Realigning Perspectives to Reflect Contemporary Issues Related to Judicious Use of Marine Resources in Aquafeeds. N. Am. J. Aquac..

[4] Pavan Kumar B., Ramudu K.R., Devi B.C. (2014). Mini Review on Incorporation of Cotton Seed Meal, an Alternative to Fish Meal in Aquaculture Feeds. Int. J. Biol. Res..

[5] Morais S., Pratoomyot J., Taggart J.B., Bron J.E., Guy D.R., Bell J.G., Tocher D.R. (2011). Genotype-Specific Responses in Atlantic Salmon (*Salmo salar*) Subject to Dietary Fish Oil Replacement by Vegetable Oil: A Liver Transcriptomic Analysis. BMC Genom..

[6] Hardy R.W. (2010). Utilization of Plant Proteins in Fish Diets: Effects of Global Demand and Supplies of Fishmeal. Aquac. Res..

[7] Hussain S.M., Bano A.A., Ali S., Rizwan M., Adrees M., Zahoor A.F., Sarker P.K., Hussain M., Arsalan M.Z.-u.-H., Yong J.W.H. (2024). Substitution of Fishmeal: Highlights of Potential Plant Protein Sources for Aquaculture Sustainability. Heliyon.

[8] Perera E., Simó-Mirabet P., Shin H.S., Rosell-Moll E., Naya-Catalá F., de las Heras V., Martos-Sitcha J.A., Karalazos V., Armero E., Arizcun M. (2019). Selection for Growth Is Associated in Gilthead Sea Bream (*Sparus aurata*) with Diet Flexibility, Changes in Growth Patterns and Higher Intestine Plasticity. Aquaculture.

[9] Xu W., Jin J., Han D., Liu H., Zhu X., Yang Y., Xie S. (2019). Physiological and Transcriptomic Responses to Fishmeal-Based Diet and Rapeseed Meal-Based Diet in Two Strains of Gibel Carp (*Carassius gibelio*). Fish Physiol. Biochem..

[10] Sabbagh M., Schiavone R., Brizzi G., Sicuro B., Zilli L., Vilella S. (2019). Poultry By-Product Meal as an Alternative to Fish Meal in the Juvenile Gilthead Seabream (*Sparus aurata*) Diet. Aquaculture.

[11] Aragão C., Gonçalves A.T., Costas B., Azeredo R., Xavier M.J., Engrola S. (2022). Alternative Proteins for Fish Diets: Implications beyond Growth. Animals.

[12] Liew C.-C., Ma J., Tang H.-C., Zheng R., Dempsey A.A. (2006). The Peripheral Blood Transcriptome Dynamically Reflects System Wide Biology: A Potential Diagnostic Tool. J. Lab. Clin. Med..

[13] Angelakopoulos R., Tsipourlianos A., Moutou K.A., Fytsili A.E., Tsingene A., Galliopoulou E., Papaharisis L., Mamuris Z., Giannoulis T., Dimitroglou A. (2025). Selection of Nonlethal Early Biomarkers to Predict Gilthead Seabream (*Sparus aurata*) Growth. Aquac. Nutr..

[14] HAPO (2023). Annual Report: Aquaculture in Greece. https://fishfromgreece.com/wp-content/uploads/2023/10/HAPO_AR23_WEB-NEW.pdf.

[15] FEAP (2024). European Aquaculture Production Report.

[16] Andrew S.C., Primmer C.R., Debes P.V., Erkinaro J., Verta J.P. (2021). The Atlantic Salmon Whole Blood Transcriptome and How It Relates to Major Locus Maturation Genotypes and Other Tissues. Mar. Genom..

[17] Jégou M., Gondret F., Vincent A., Tréfeu C., Gilbert H., Louveau I. (2016). Whole Blood Transcriptomics Is Relevant to Identify Molecular Changes in Response to Genetic Selection for Feed Efficiency and Nutritional Status in the Pig. PLoS ONE.

[18] Götting M., Nikinmaa M.J. (2017). Transcriptomic Analysis of Young and Old Erythrocytes of Fish. Front. Physiol..

[19] Xie F., Xiao P., Chen D., Xu L., Zhang B. (2012). MiRDeepFinder: A MiRNA Analysis Tool for Deep Sequencing of Plant Small RNAs. Plant Mol. Biol..

[20] Čikoš Š., Bukovská A., Koppel J. (2007). Relative Quantification of MRNA: Comparison of Methods Currently Used for Real-Time PCR Data Analysis. BMC Mol. Biol..

[21] Vandesompele J., De Preter K., Pattyn F., Poppe B., Van Roy N., De Paepe A., Speleman F. (2002). Accurate Normalization of Real-Time Quantitative RT-PCR Data by Geometric Averaging of Multiple Internal Control Genes. Genome Biol..

[22] R Core Team (2021). R: A Language and Environment for Statistical Computing.

[23] RStudio Team (2020). RStudio: Integrated Development for R. RStudio.

[24] Shapiro S.S., Wilk M.B. (1965). An Analysis of Variance Test for Normality (Complete Samples). Biometrika.

[25] Dodge Y. (2008). Kruskal-Wallis Test. The Concise Encyclopedia of Statistics.

[26] Wickham H. (2016). Ggplot2: Elegant Graphics for Data Analysis.

[27] Kassambara A. (2023). ggpubr: ‘ggplot2’ Based Publication Ready Plots. R Package Version 0.6.0. https://rpkgs.datanovia.com/ggpubr/.

[28] Leduc A., Zatylny-Gaudin C., Robert M., Corre E., Corguille G.L., Castel H., Lefevre-Scelles A., Fournier V., Gisbert E., Andree K.B. (2018). Dietary Aquaculture By-Product Hydrolysates: Impact on the Transcriptomic Response of the Intestinal Mucosa of European Seabass (*Dicentrarchus labrax*) Fed Low Fish Meal Diets. BMC Genom..

[29] Francis G., Makkar H.P.S., Becker K. (2001). Antinutritional Factors Present in Plant-Derived Alternate Fish Feed Ingredients and Their Effects in Fish. Aquaculture.

[30] Harter T.S., Brauner C.J. (2021). Teleost Red Blood Cells Actively Enhance the Passive Diffusion of Oxygen That Was Discovered by August Krogh. Comp. Biochem. Physiol.-Part A Mol. Integr. Physiol..

[31] Gilmour K.M., Perry S.F. (2009). Carbonic Anhydrase and Acid-Base Regulation in Fish. J. Exp. Biol..

[32] Thomas S., Egée S. (1998). Fish Red Blood Cells: Characteristics and Physiological Role of the Membrane Ion Transporters. Comp. Biochem. Physiol. Part A Mol. Integr. Physiol..

[33] Dichiera A.M., Khursigara A.J., Esbaugh A.J. (2021). The Effects of Warming on Red Blood Cell Carbonic Anhydrase Activity and Respiratory Performance in a Marine Fish. Comp. Biochem. Physiol.-Part A Mol. Integr. Physiol..

[34] Quyet D.H., Dung P.T., Le Na N.T., Dung M.B., Huong N.T.M., Vuong T.P. (2025). Hematological Profile of Red Drum *Sciaenops ocellatus* (Linnaeus, 1766) under Natural and Commercial Feed Nutritional Conditions. Isr. J. Aquac.-Bamidgeh.

[35] Tsipourlianos A., Cardoso J.C.R., Angelakopoulos R., Kotoula A., Power D.M., Mamuris Z., Moutou K.A. (2024). Regulatory Subfunctionalization Drives OXPHOS Evolution in Teleosts. bioRxiv.

[36] Silva-Marrero J.I., Sáez A., Caballero-Solares A., Viegas I., Almajano M.P., Fernández F., Baanante I.V., Metón I. (2017). A Transcriptomic Approach to Study the Effect of Long-Term Starvation and Diet Composition on the Expression of Mitochondrial Oxidative Phosphorylation Genes in Gilthead Sea Bream (*Sparus aurata*). BMC Genom..

[37] Torricelli M., Felici A., Branciari R., Trabalza-Marinucci M., Galarini R., Biagetti M., Manfrin A., Boriani L., Radicchi E., Sebastiani C. (2024). Gene Expression Study in Gilthead Seabream (*Sparus aurata*): Effects of Dietary Supplementation with Olive Oil Polyphenols on Immunity, Metabolic, and Oxidative Stress Pathways. Int. J. Mol. Sci..

[38] Vera L.M., Metochis C., Taylor J.F., Clarkson M., Skjærven K.H., Migaud H., Tocher D.R. (2017). Early Nutritional Programming Affects Liver Transcriptome in Diploid and Triploid Atlantic Salmon, *Salmo salar*. BMC Genom..

[39] Han Y.K., Xu Y.C., Luo Z., Zhao T., Zheng H., Tan X.Y. (2022). Fish Meal Replacement by Mixed Plant Protein in the Diets for Juvenile Yellow Catfish *Pelteobagrus gulvidraco*: Effects on Growth Performance and Health Status. Aquac. Nutr..

[40] Calduch-Giner J.A., Sitjà-Bobadilla A., Davey G.C., Cairns M.T., Kaushik S., Pérez-Sánchez J. (2012). Dietary Vegetable Oils Do Not Alter the Intestine Transcriptome of Gilthead Sea Bream (*Sparus aurata*), but Modulate the Transcriptomic Response to Infection with Enteromyxum Leei. BMC Genom..

[41] Xu H., Liao Z., Wang C., Wei Y., Liang M. (2019). Hepatic Transcriptome of the Euryhaline Teleost Japanese Seabass *(Lateolabrax japonicus*) Fed Diets Characterized by α-Linolenic Acid or Linoleic Acid. Comp. Biochem. Physiol. Part D Genom. Proteom..

[42] de Magalhães C.R., Sandoval K., Kagan F., McCormack G., Schrama D., Carrilho R., Farinha A.P., Cerqueira M., Rodrigues P.M. (2024). Transcriptomic Changes behind *Sparus aurata* Hepatic Response to Different Aquaculture Challenges: An RNA-Seq Study and Multiomics Integration. PLoS ONE.

[43] Geay F., Ferraresso S., Zambonino-Infante J.L., Bargelloni L., Quentel C., Vandeputte M., Kaushik S., Cahu C.L., Mazurais D. (2011). Effects of the Total Replacement of Fish-Based Diet with Plant-Based Diet on the Hepatic Transcriptome of Two European Sea Bass (*Dicentrarchus labrax*) Half-Sibfamilies Showing Different Growth Rates with the Plant-Based Diet. BMC Genom..

[44] De Santis C., Crampton V.O., Bicskei B., Tocher D.R. (2015). Replacement of Dietary Soy- with Air Classified Faba Bean Protein Concentrate Alters the Hepatic Transcriptome in Atlantic Salmon (*Salmo salar*) Parr. Comp. Biochem. Physiol. Part D Genom. Proteom..

[45] Estruch G., Collado M.C., Monge-Ortiz R., Tomás-Vidal A., Jover-Cerdá M., Peñaranda D.S., Pérez Martínez G., Martínez-Llorens S. (2018). Long-Term Feeding with High Plant Protein Based Diets in Gilthead Seabream (*Sparus aurata*, L.) Leads to Changes in the Inflammatory and Immune Related Gene Expression at Intestinal Level. BMC Vet. Res..

[46] Dias J., Alvarez M.J., Arzel J., Corraze G., Diez A., Bautista J.M., Kaushik S.J. (2005). Dietary Protein Source Affects Lipid Metabolism in the European Seabass (*Dicentrarchus labrax*). Comp. Biochem. Physiol. Part A Mol. Integr. Physiol..

[47] Kortner T.M., Björkhem I., Krasnov A., Timmerhaus G., Krogdahl Å. (2014). Dietary Cholesterol Supplementation to a Plant-Based Diet Suppresses the Complete Pathway of Cholesterol Synthesis and Induces Bile Acid Production in Atlantic Salmon (*Salmo salar* L.). Br. J. Nutr..

[48] Kemski M.M., Rappleye C.A., Dabrowski K., Bruno R.S., Wick M. (2020). Transcriptomic Response to Soybean Meal-Based Diets as the First Formulated Feed in Juvenile Yellow Perch (*Perca flavescens*). Sci. Rep..

[49] Krogdahl Å., Penn M., Thorsen J., Refstie S., Bakke A.M. (2010). Important Antinutrients in Plant Feedstuffs for Aquaculture: An Update on Recent Findings Regarding Responses in Salmonids. Aquac. Res..

[50] Bermejo-Nogales A., Calduch-Giner J.A., Pérez-Sánchez J. (2015). Unraveling the Molecular Signatures of Oxidative Phosphorylation to Cope with the Nutritionally Changing Metabolic Capabilities of Liver and Muscle Tissues in Farmed Fish. PLoS ONE.

[51] Kristoffersen S., Tobiassen T., Steinsund V., Olsen R.L. (2006). Slaughter Stress, Postmortem Muscle PH and Rigor Development in Farmed Atlantic Cod (*Gadus morhua* L.). Int. J. Food Sci. Technol..

[52] Cai W., Liu H., Han D., Zhu X., Jin J., Yang Y., Xie S. (2022). Complete Replacement of Fishmeal With Plant Protein Ingredients in Gibel Carp (*Carassius auratus gibelio*) Diets by Supplementation With Essential Amino Acids Without Negative Impact on Growth Performance and Muscle Growth-Related Biomarkers. Front. Mar. Sci..

