# Supplementary material for: Red Blood Cell Transcriptome Reflects Physiological Responses to Alternative Nutrient Sources in Gilthead Seabream (Sparus aurata)
Source: Animals (Basel). 2025 Apr 30;15(9):1279. doi: 10.3390/ani15091279 (PMC12070918; doi:10.3390/ani15091279)
Supplement: Supplementary file 1 [file animals-15-01279-s001.zip › animals-3539890-Supplementary done.pdf]

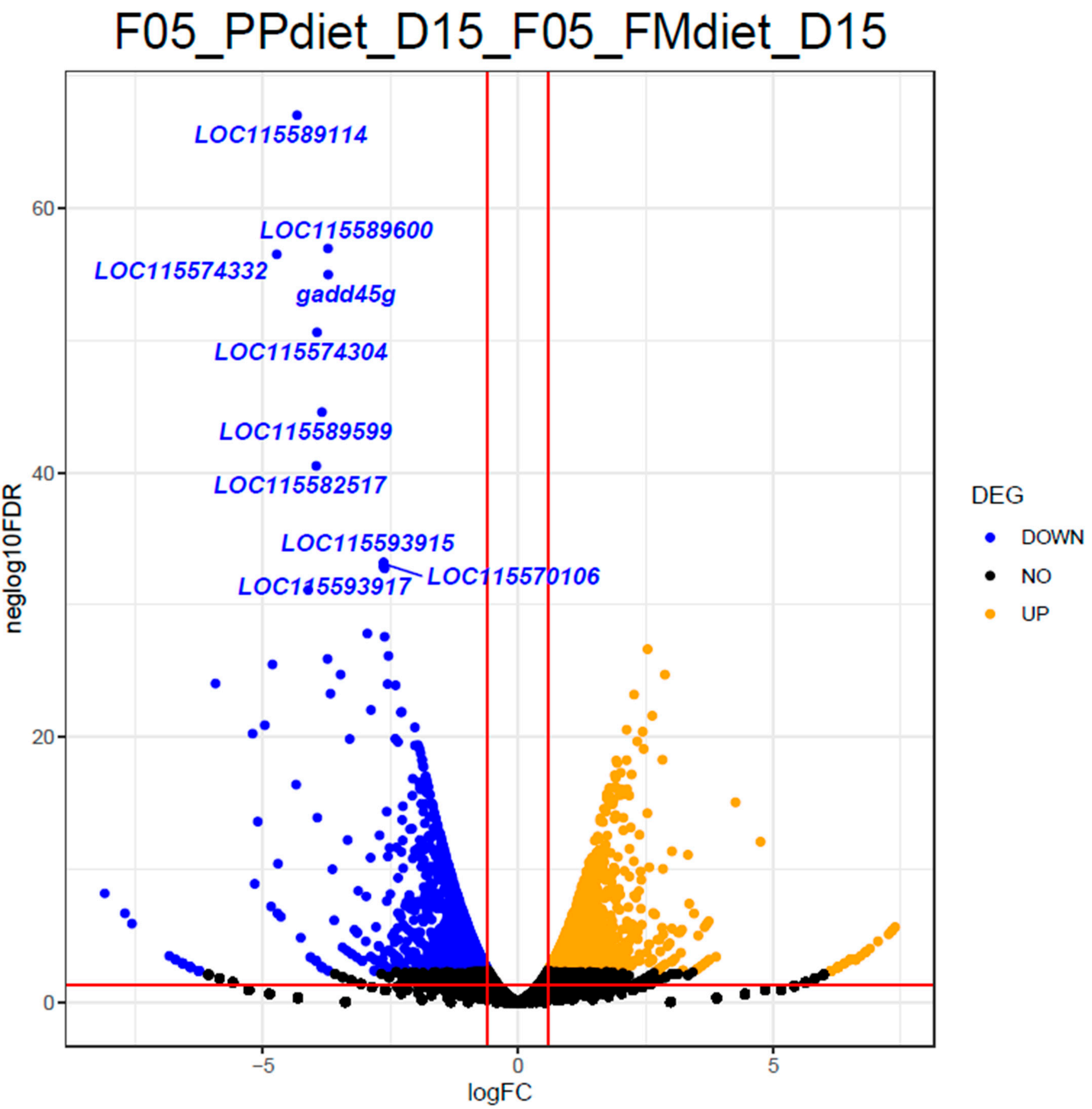

Supplementary Figure 1. Volcano plots of differentially expressed genes in F05. Condition tested: PPvsFM diets on D15. Statistically significant (p value< 0.05) down regulated DEGS in condition 2 are depicted in blue, up regulated DEGS in condition 2 are depicted in orange, and black shows non statistically significant regulated genes. The name of the top ten up and down DEGs is also given in the plot.

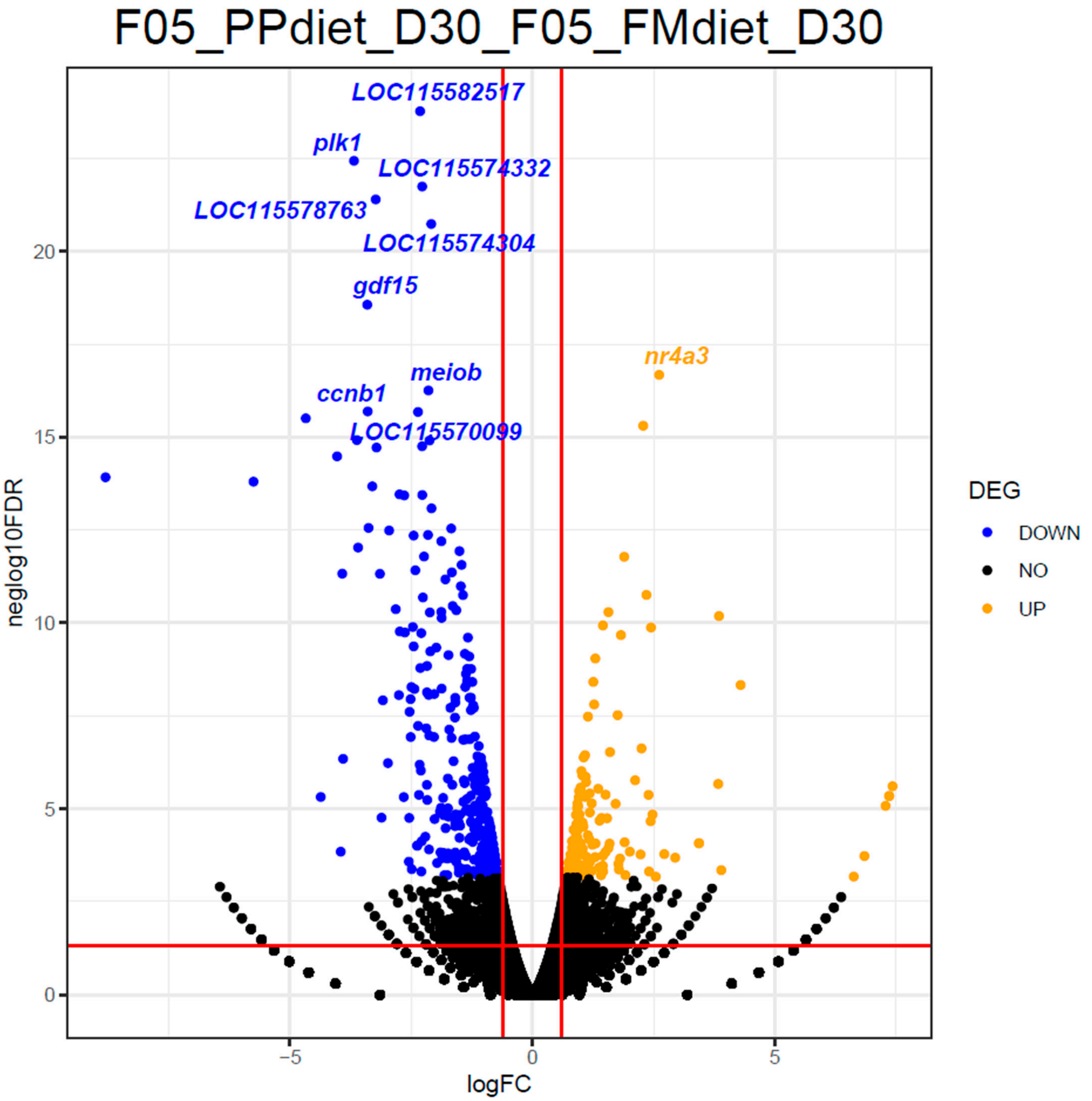

Supplementary Figure 2. Volcano plots of differentially expressed genes in F05. Condition tested: PPvsFM diets on D30. Statistically significant (p value< 0.05) down regulated DEGS in condition 2 are depicted in blue, up regulated DEGS in condition 2 are depicted in orange, and black shows non statistically significant regulated genes. The name of the top ten up and down DEGs is also given in the plot.

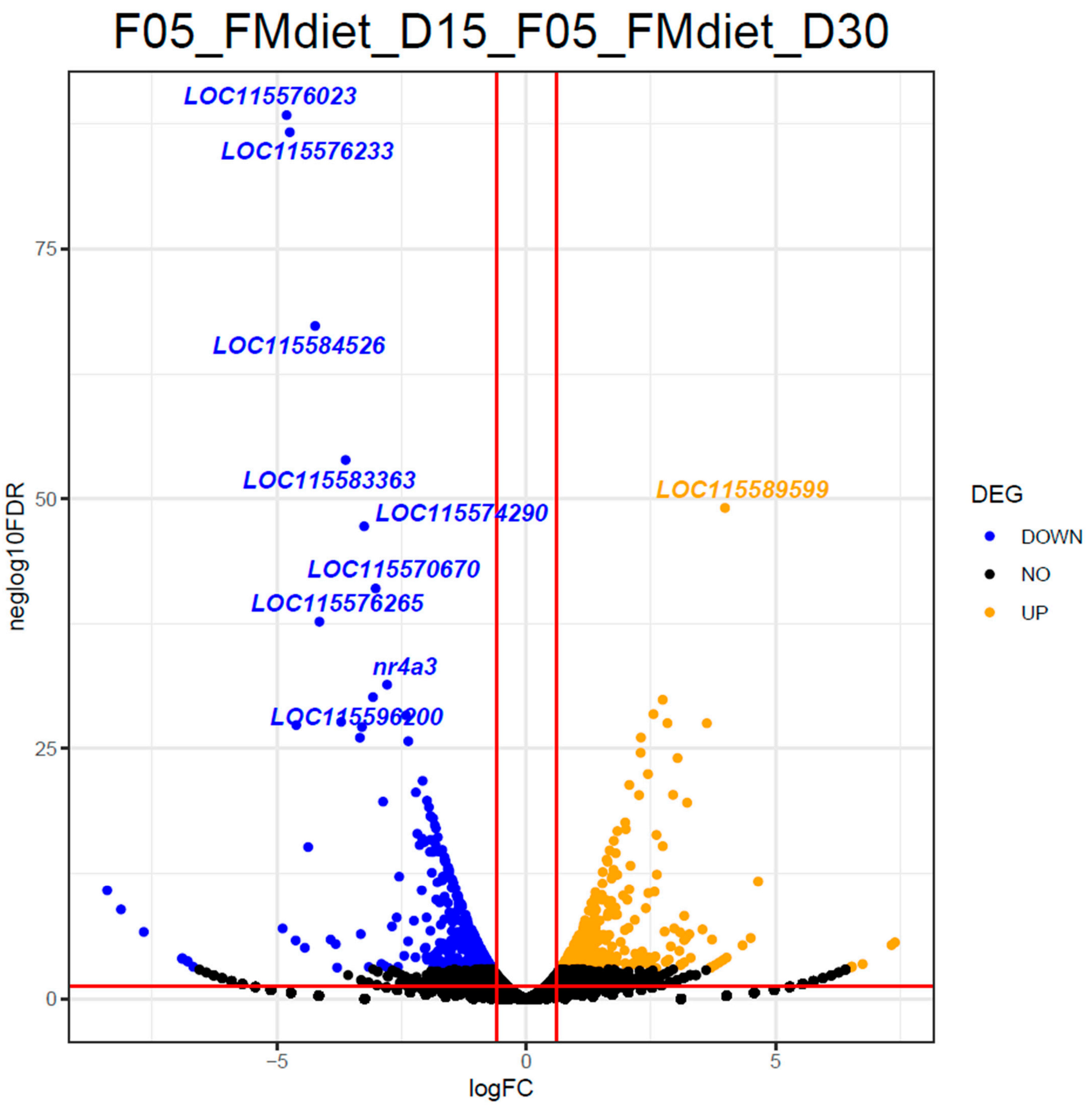

Supplementary Figure 3. Volcano plots of differentially expressed genes in F05. Condition tested: FM diet: D15 vs D30. Statistically significant (p value< 0.05) down regulated DEGS in condition 2 are depicted in blue, up regulated DEGS in condition 2 are depicted in orange, and black shows non statistically significant regulated genes. The name of the top ten up and down DEGs is also given in the plot.

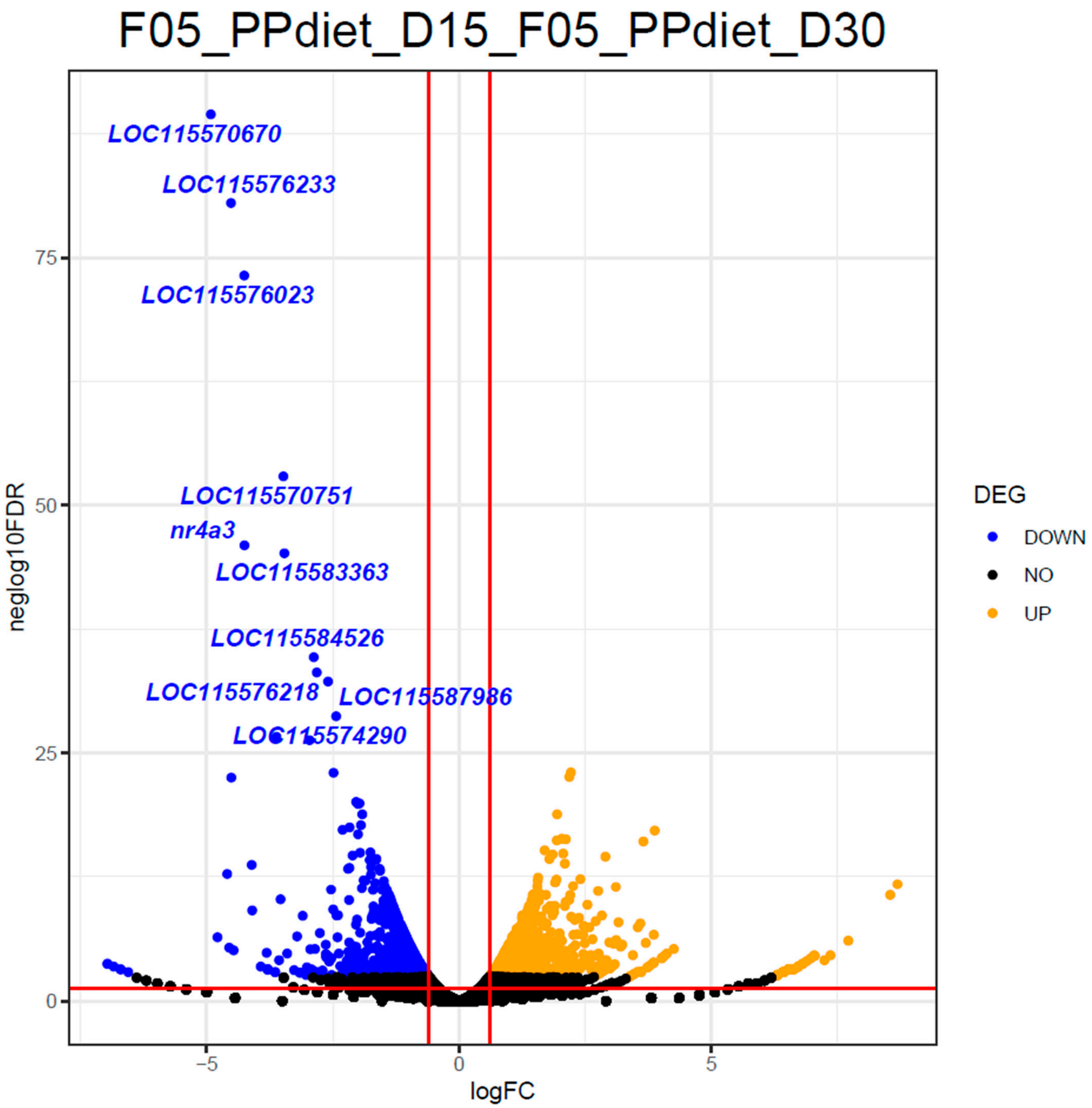

Supplementary Figure 4. Volcano plots of differentially expressed genes in F05. Condition tested: PP diet: D15 vs D30. Statistically significant (p value< 0.05) down regulated DEGS in condition 2 are depicted in blue, up regulated DEGS in condition 2 are depicted in orange, and black shows non statistically significant regulated genes. The name of the top ten up and down DEGs is also given in the plot.

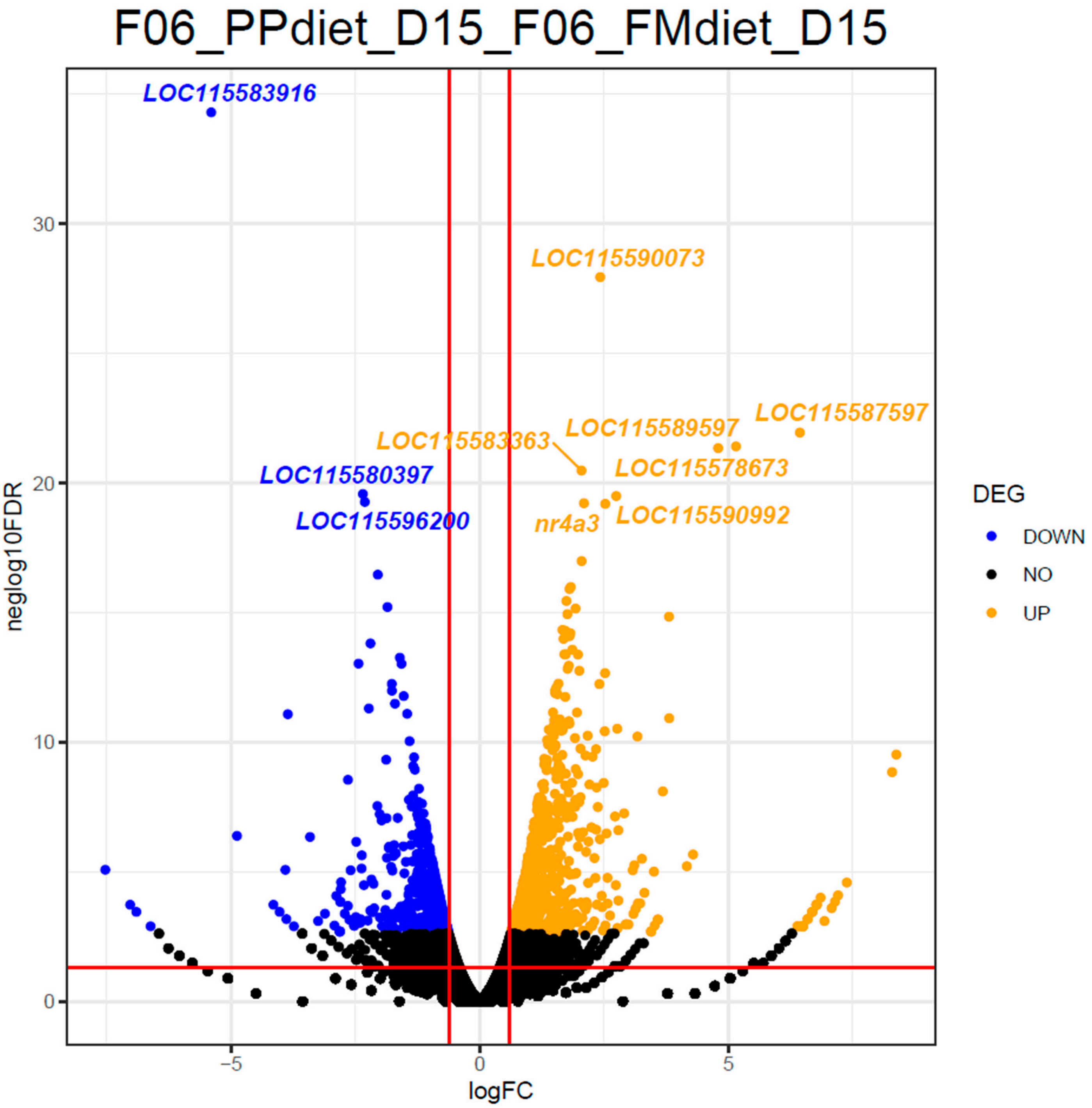

Supplementary Figure 5. Volcano plots of differentially expressed genes in F06. Condition tested: PPvsFM diets on D15. Statistically significant (p value< 0.05) down regulated DEGS in condition 2 are depicted in blue, up regulated DEGS in condition 2 are depicted in orange, and black shows non statistically significant regulated genes. The name of the top ten up and down DEGs is also given in the plot.

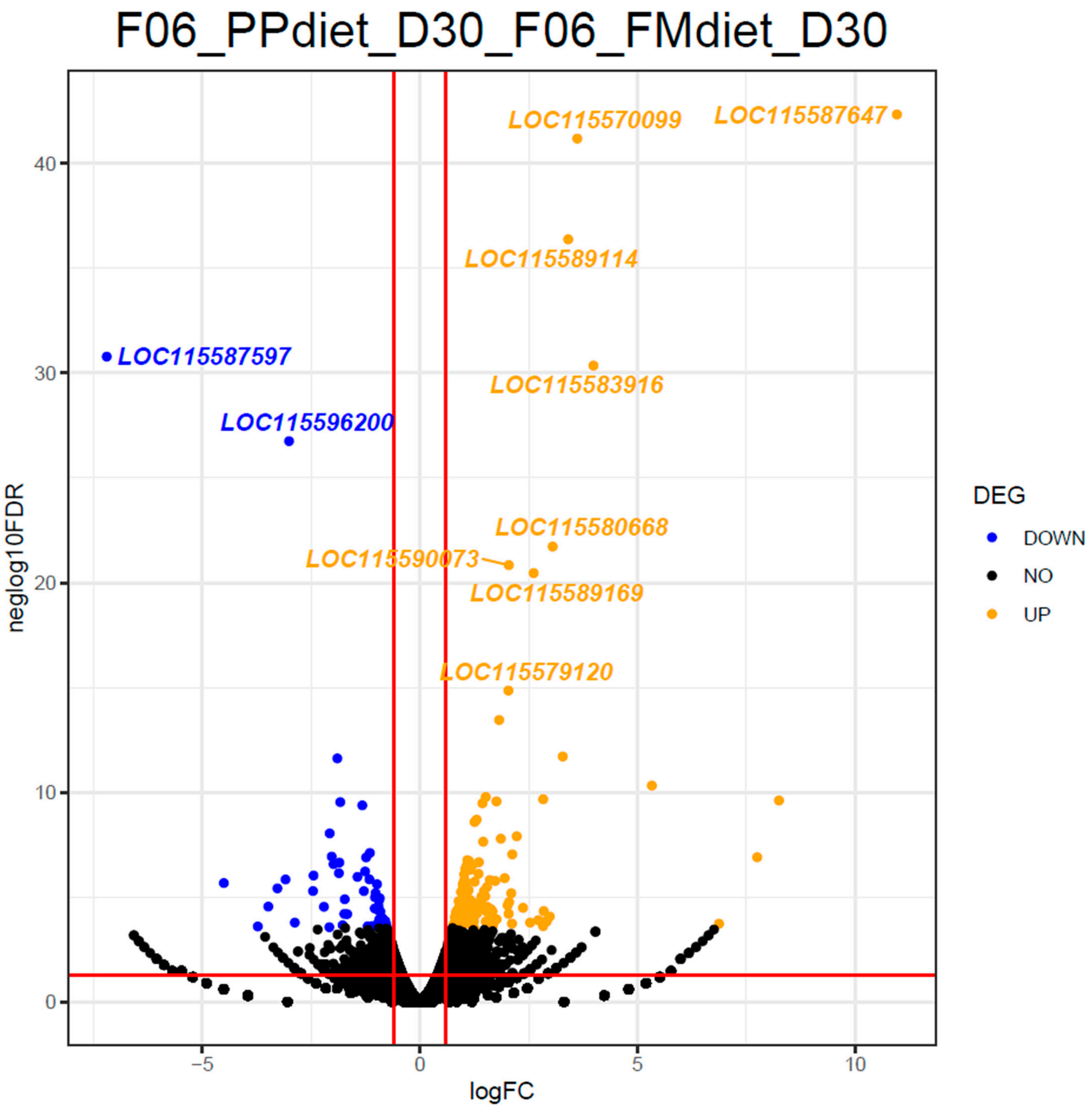

Supplementary Figure 6. Volcano plots of differentially expressed genes in F06. Condition tested: PPvsFM diets on D30. Statistically significant (p value< 0.05) down regulated DEGS in condition 2 are depicted in blue, up regulated DEGS in condition 2 are depicted in orange, and black shows non statistically significant regulated genes. The name of the top ten up and down DEGs is also given in the plot.

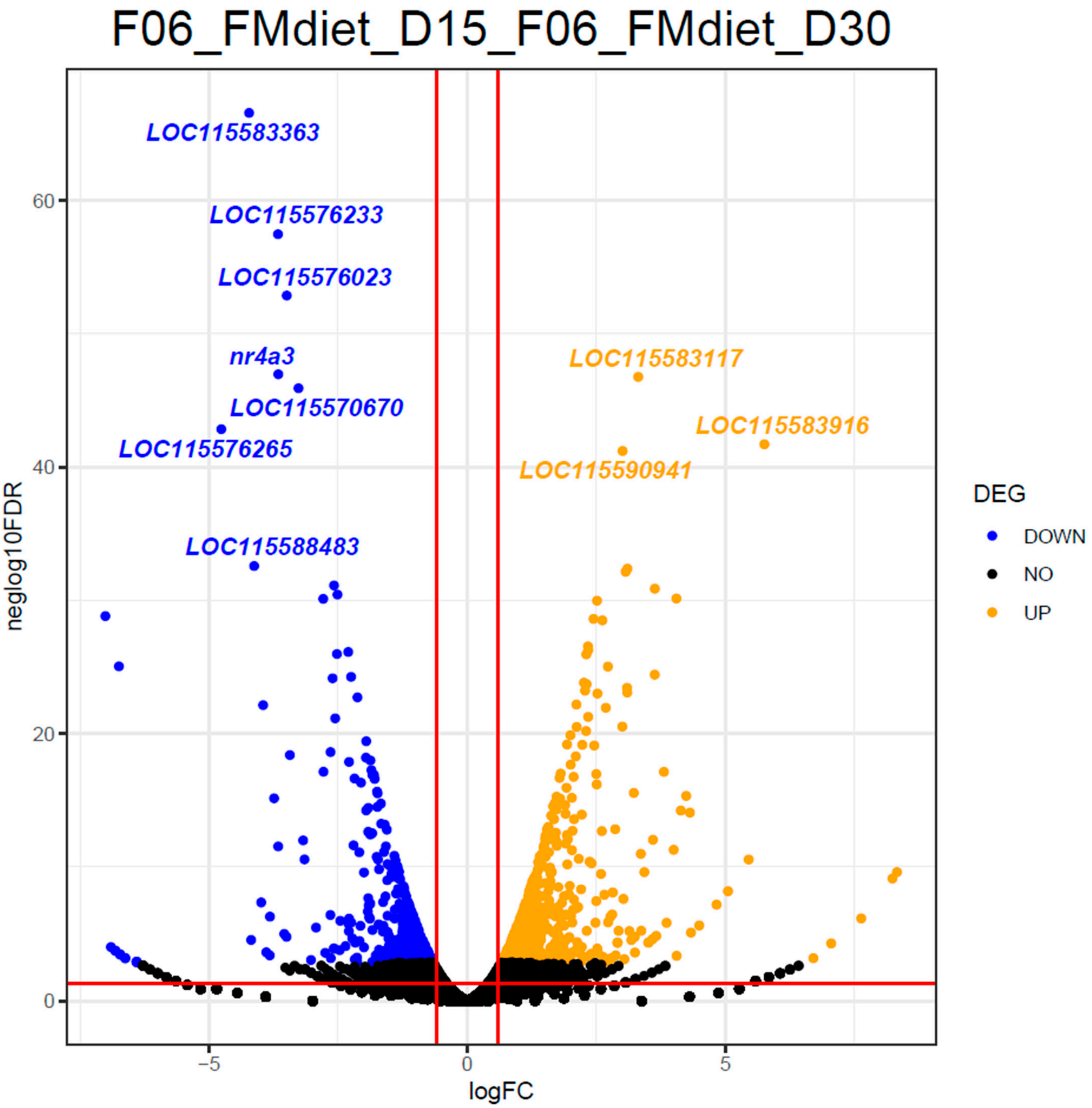

Supplementary Figure 7. Volcano plots of differentially expressed genes in F*06*. Condition tested: FM diet: D15 vs D30. Statistically significant (p value< 0.05) down regulated DEGS in condition 2 are depicted in blue, up regulated DEGS in condition 2 are depicted in orange, and black shows non statistically significant regulated genes. The name of the top ten up and down DEGs is also given in the plot.

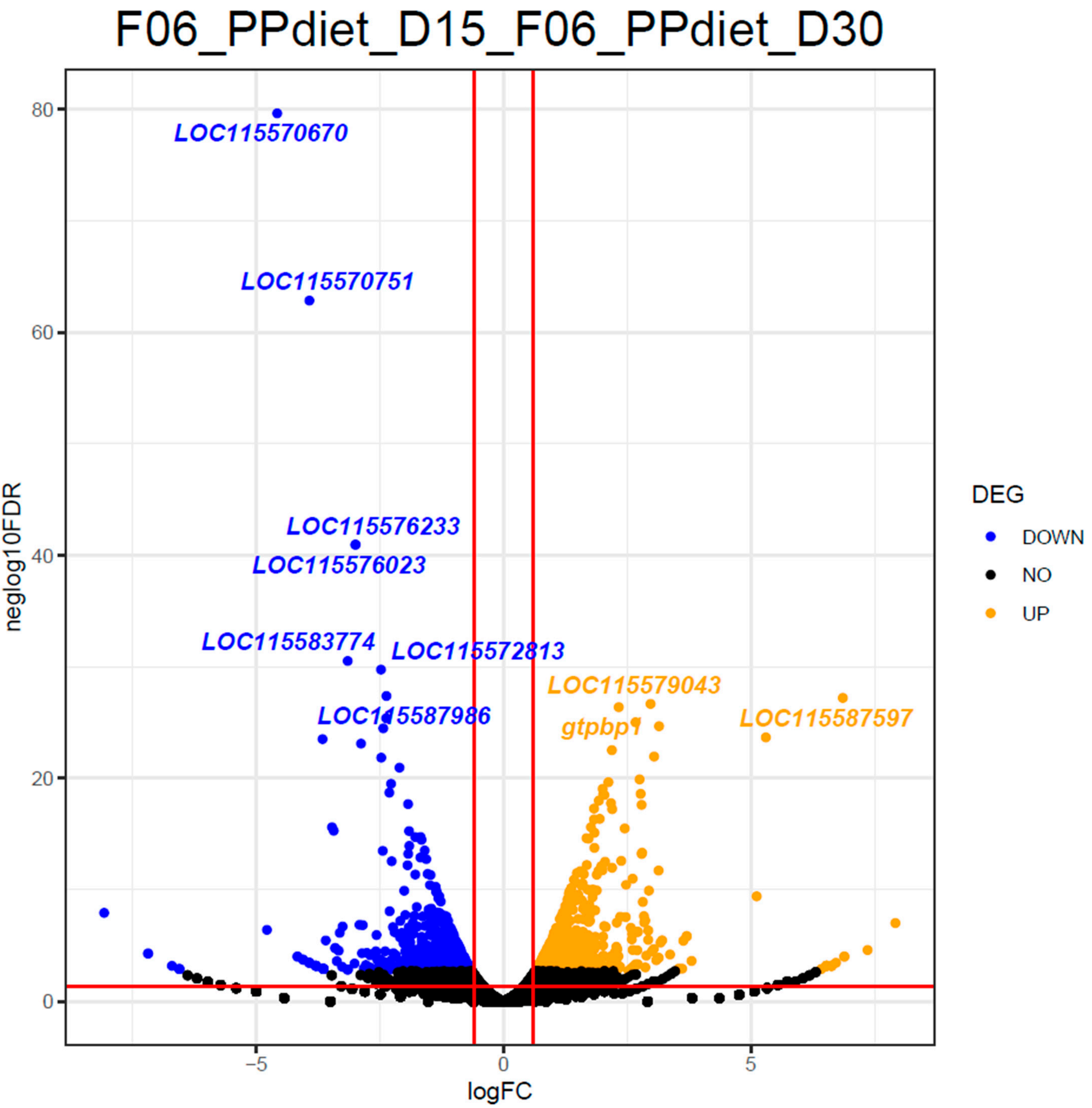

Supplementary Figure 8. Volcano plots of differentially expressed genes in F06. Condition tested: PP diet: D15 vs D30. Statistically significant (p value< 0.05) down regulated DEGS in condition 2 are depicted in blue, up regulated DEGS in condition 2 are depicted in orange, and black shows non statistically significant regulated genes. The name of the top ten up and down DEGs is also given in the plot.

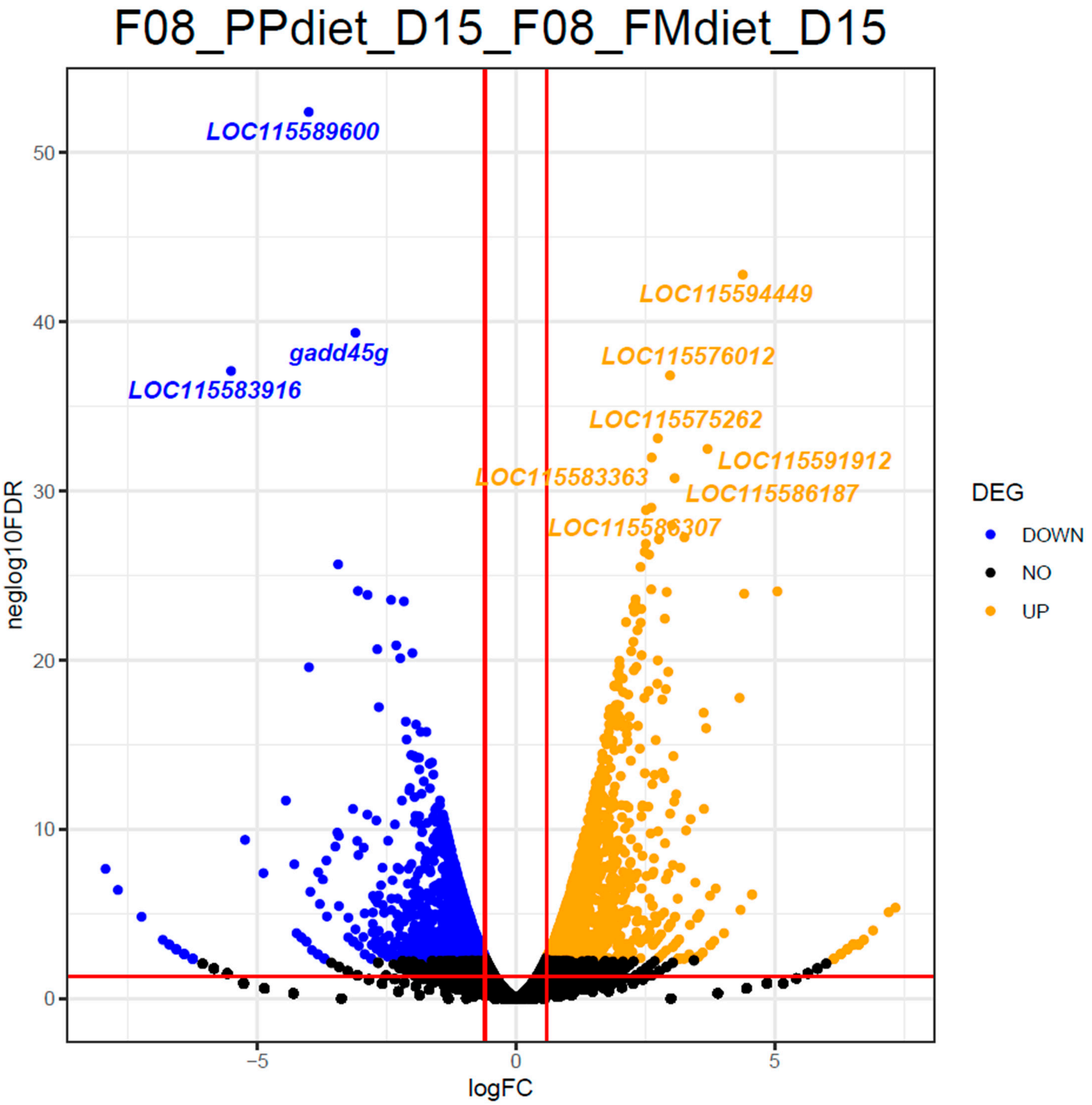

Supplementary Figure 9. Volcano plots of differentially expressed genes in F*08*. Condition tested: PPvsFM diets on D15. Statistically significant (p value< 0.05) down regulated DEGS in condition 2 are depicted in blue, up regulated DEGS in condition 2 are depicted in orange, and black shows non statistically significant regulated genes. The name of the top ten up and down DEGs is also given in the plot.

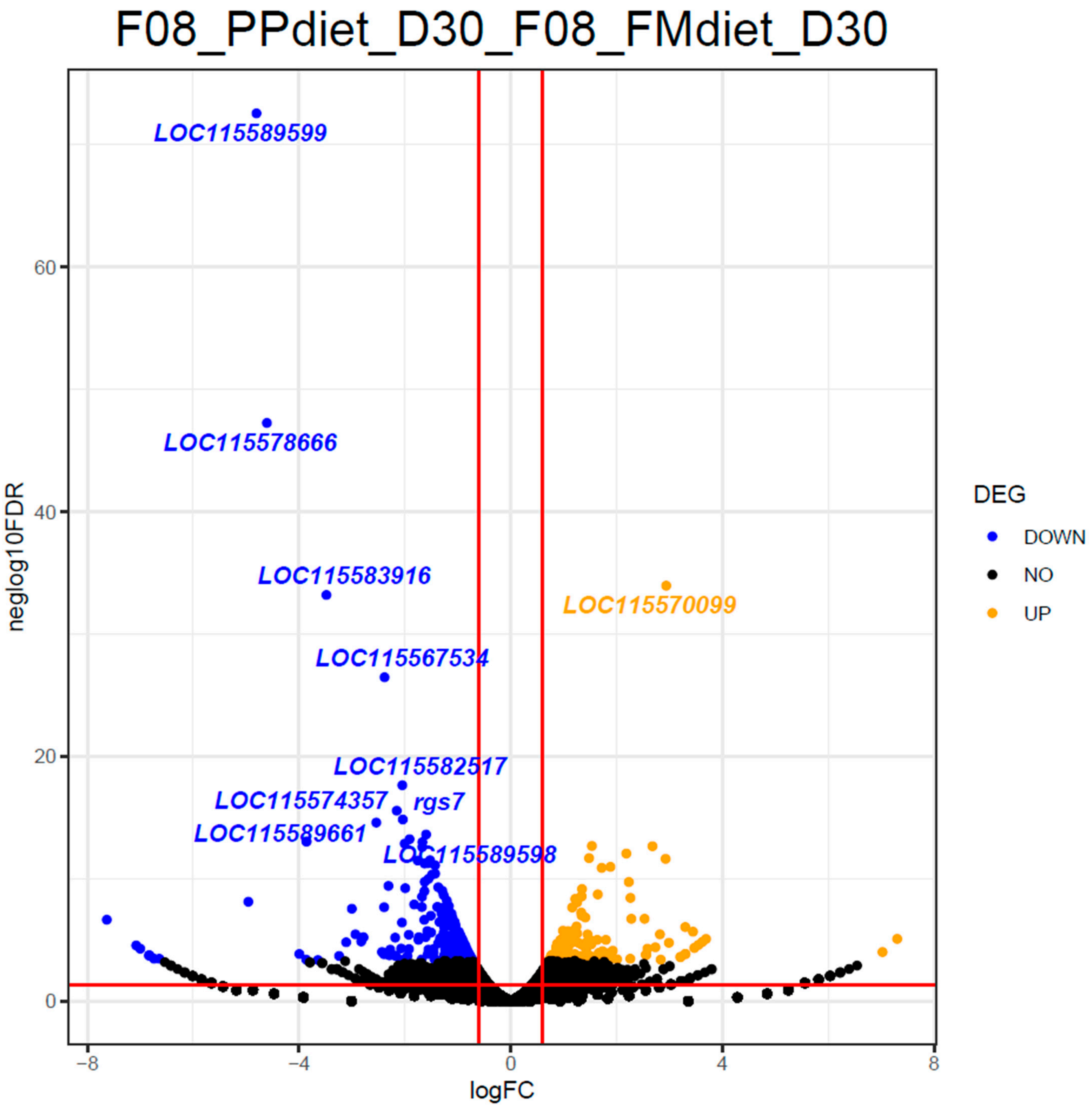

Supplementary Figure 10. Volcano plots of differentially expressed genes in F*08*. Condition tested: PPvsFM diets on D30. Statistically significant (p value< 0.05) down regulated DEGS in condition 2 are depicted in blue, up regulated DEGS in condition 2 are depicted in orange, and black shows non statistically significant regulated genes. The name of the top ten up and down DEGs is also given in the plot.

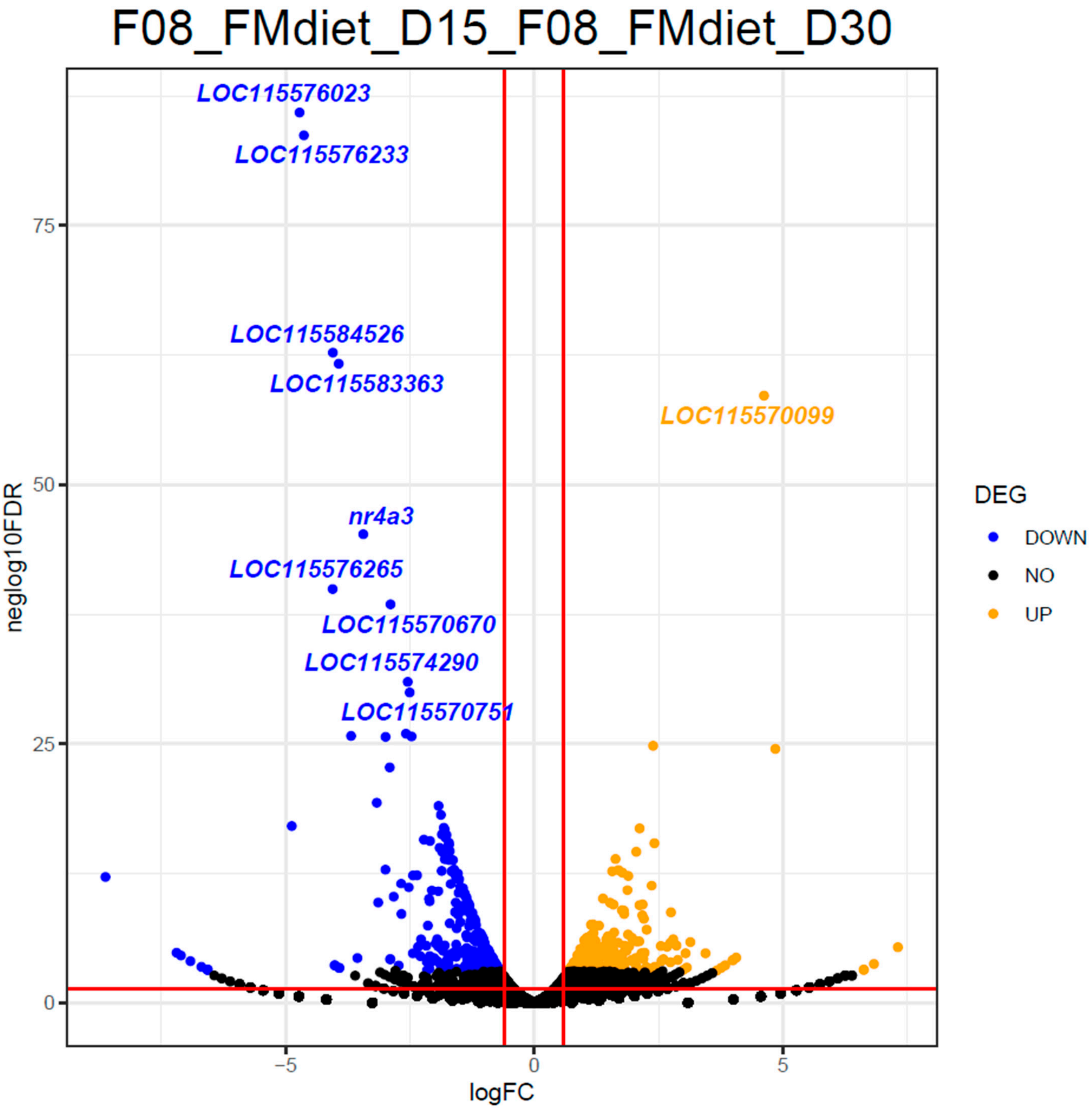

Supplementary Figure 11. Volcano plots of differentially expressed genes in F*08*. Condition tested: FM diet: D15 vs D30. Statistically significant (p value< 0.05) down regulated DEGS in condition 2 are depicted in blue, up regulated DEGS in condition 2 are depicted in orange, and black shows non statistically significant regulated genes. The name of the top ten up and down DEGs is also given in the plot.

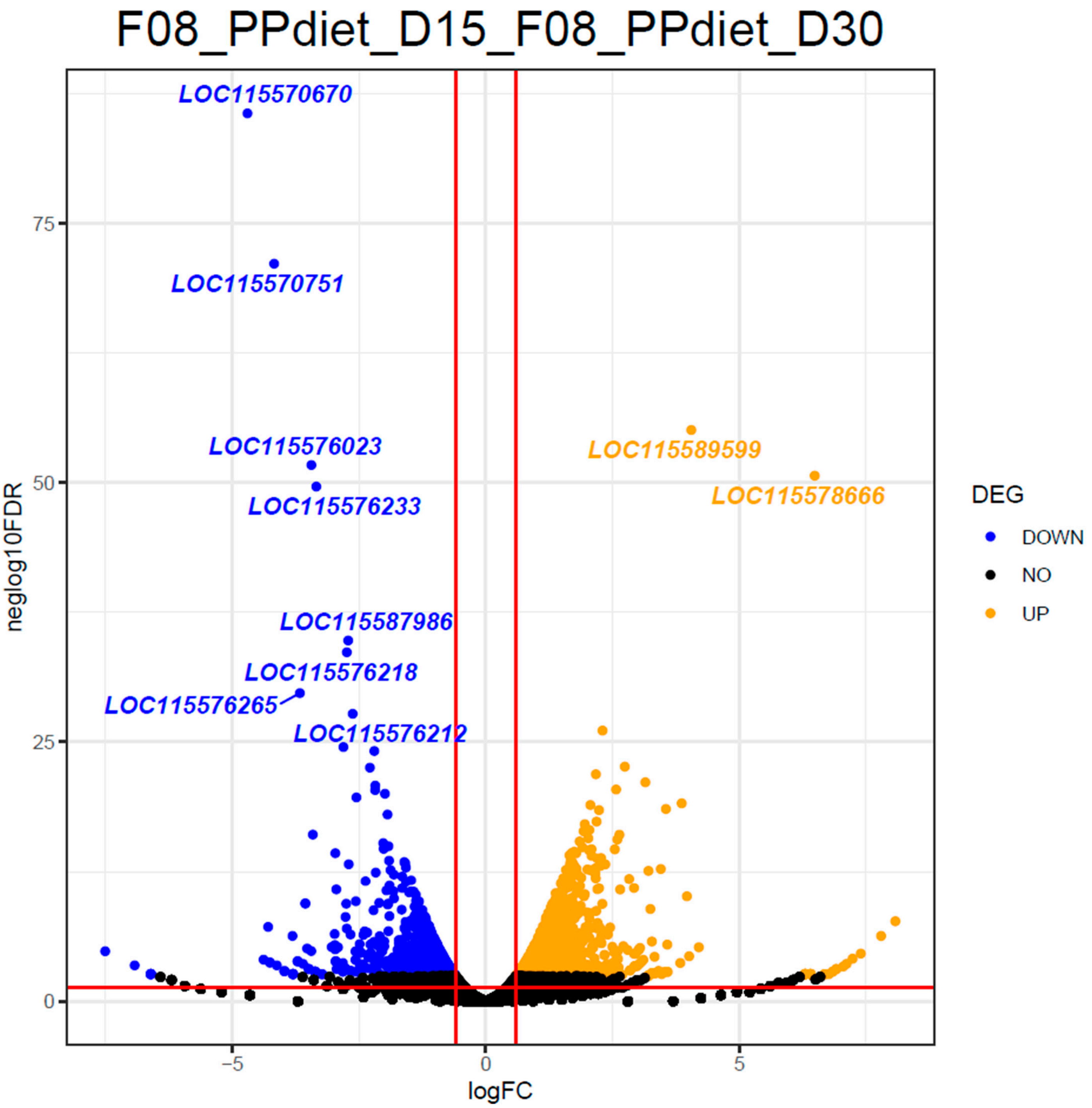

Supplementary Figure 12. Volcano plots of differentially expressed genes in F*08*. Condition tested: PP diet: D15 vs D30. Statistically significant (p value< 0.05) down regulated DEGS in condition 2 are depicted in blue, up regulated DEGS in condition 2 are depicted in orange, and black shows non statistically significant regulated genes. The name of the top ten up and down DEGs is also given in the plot.

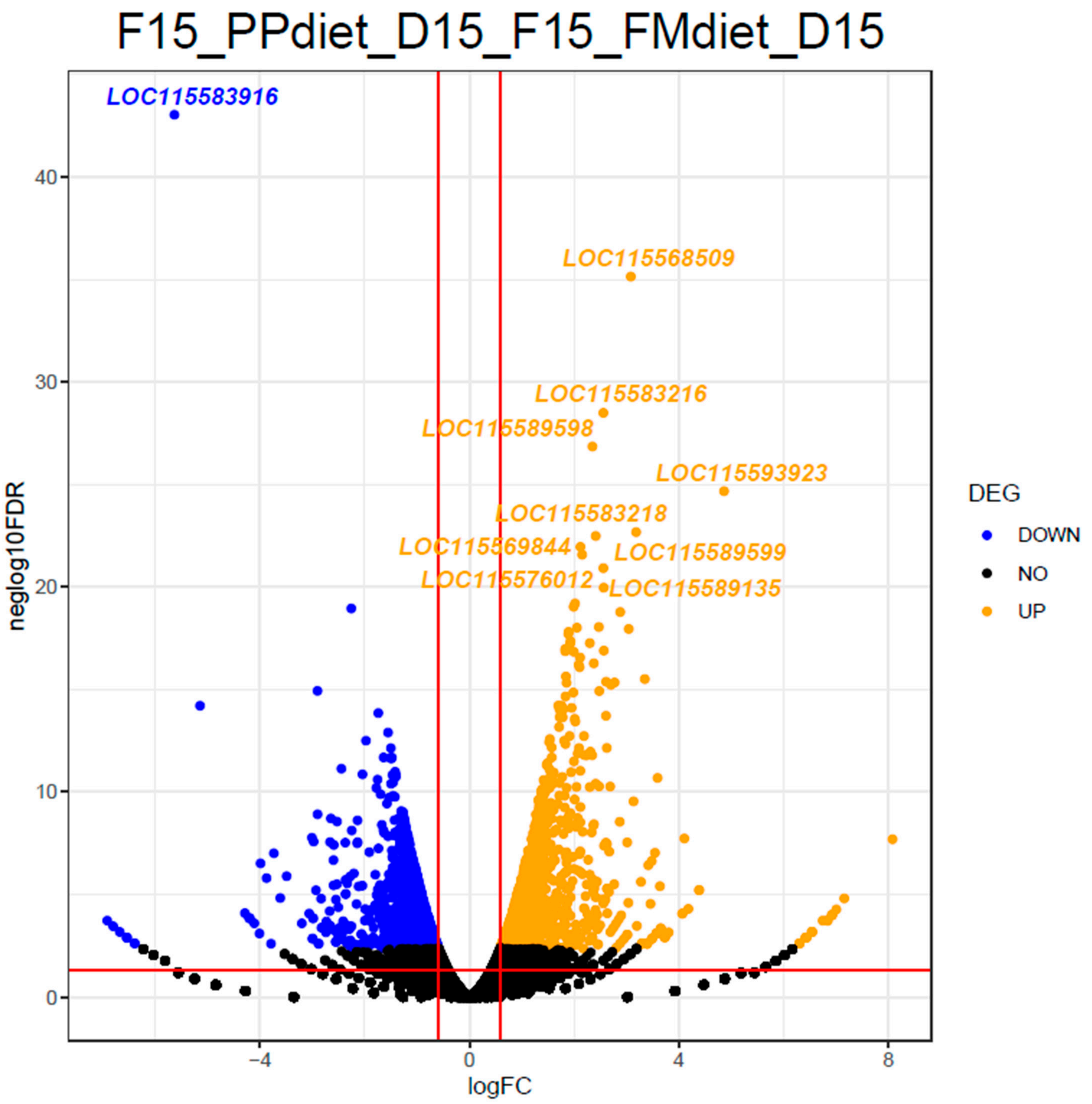

Supplementary Figure 13. Volcano plots of differentially expressed genes in F15. Condition tested: PPvsFM diets on D15. Statistically significant (p value< 0.05) down regulated DEGS in condition 2 are depicted in blue, up regulated DEGS in condition 2 are depicted in orange, and black shows non statistically significant regulated genes. The name of the top ten up and down DEGs is also given in the plot.

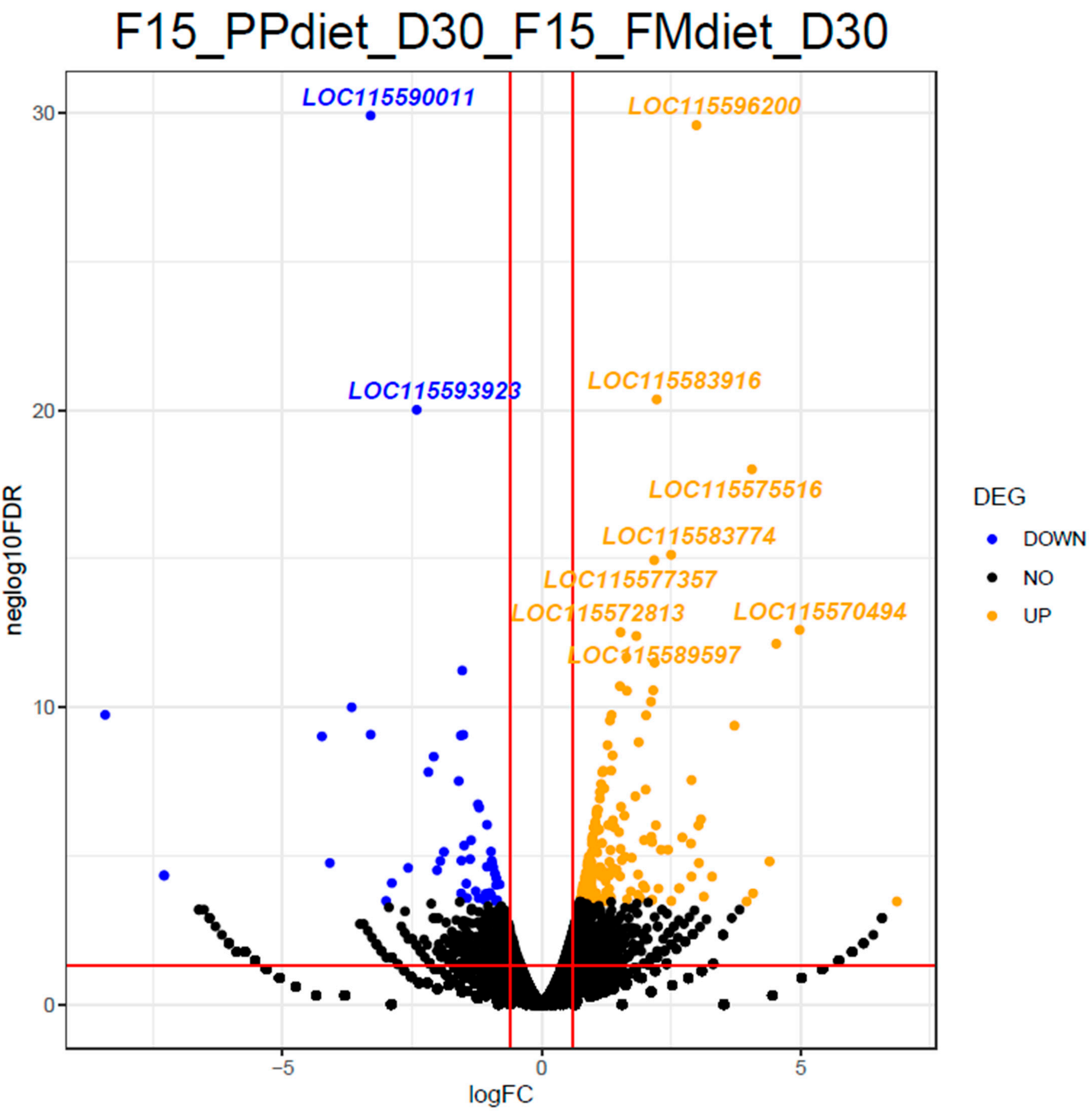

Supplementary Figure 14. Volcano plots of differentially expressed genes in F15. Condition tested: PPvsFM diets on D30. Statistically significant (p value< 0.05) down regulated DEGS in condition 2 are depicted in blue, up regulated DEGS in condition 2 are depicted in orange, and black shows non statistically significant regulated genes. The name of the top ten up and down DEGs is also given in the plot.

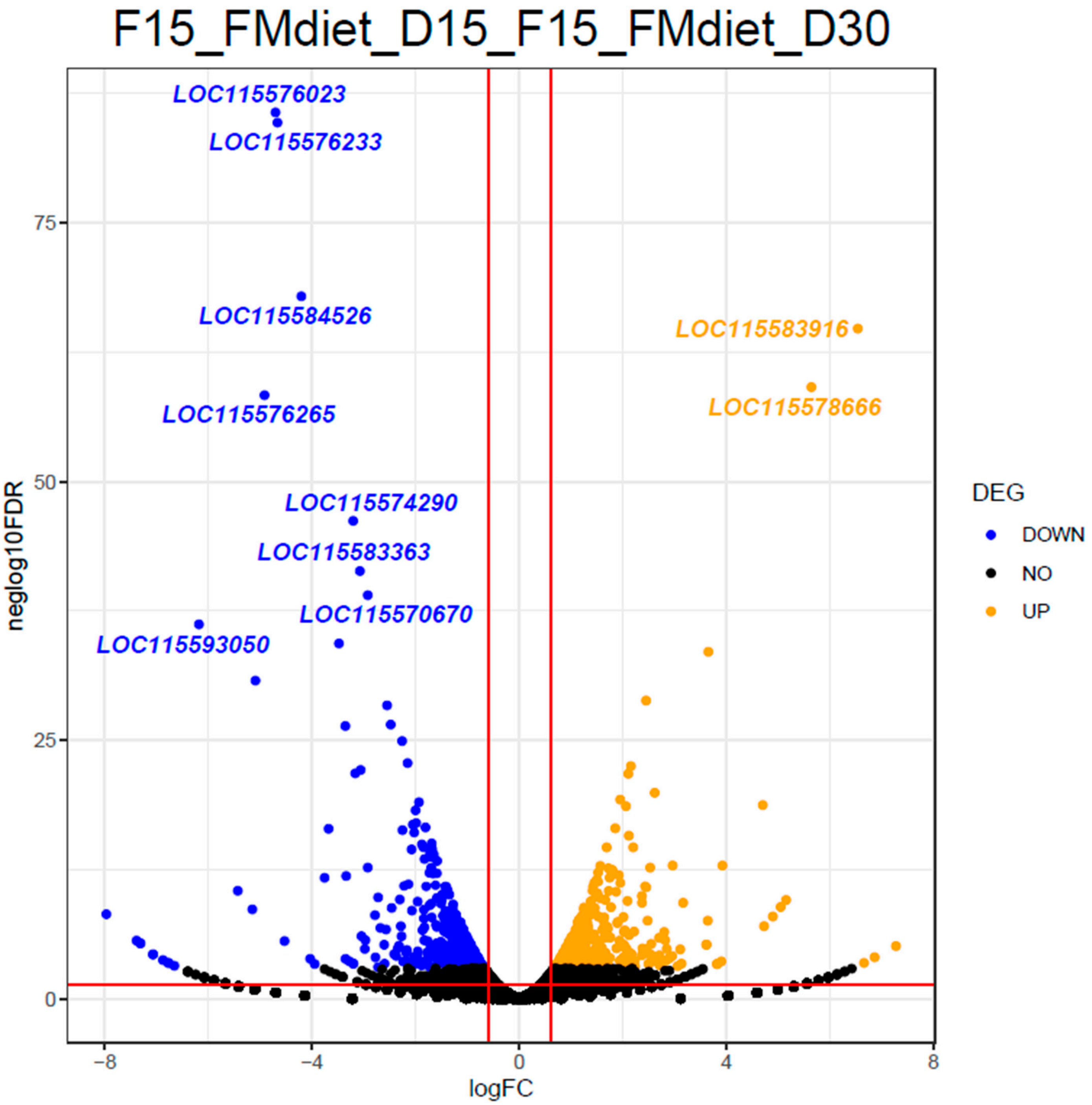

Supplementary Figure 15. Volcano plots of differentially expressed genes in F15. Condition tested: FM diet: D15 vs D30. Statistically significant (p value< 0.05) down regulated DEGS in condition 2 are depicted in blue, up regulated DEGS in condition 2 are depicted in orange, and black shows non statistically significant regulated genes. The name of the top ten up and down DEGs is also given in the plot.

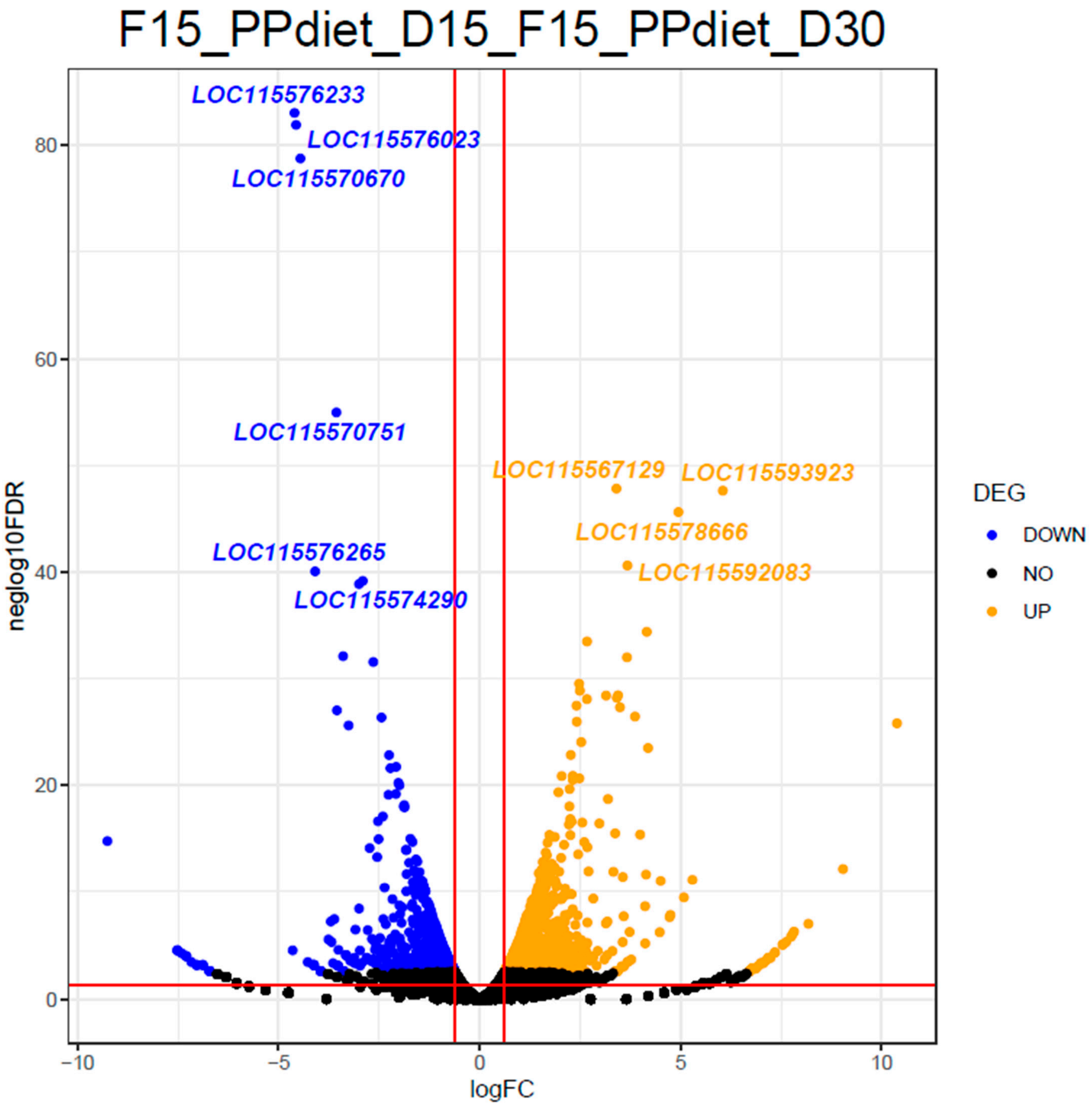

Supplementary Figure 16. Volcano plots of differentially expressed genes in F15. Condition tested: PP diet: D15 vs D30. Statistically significant (p value< 0.05) down regulated DEGS in condition 2 are depicted in blue, up regulated DEGS in condition 2 are depicted in orange, and black shows non statistically significant regulated genes. The name of the top ten up and down DEGs is also given in the plot.

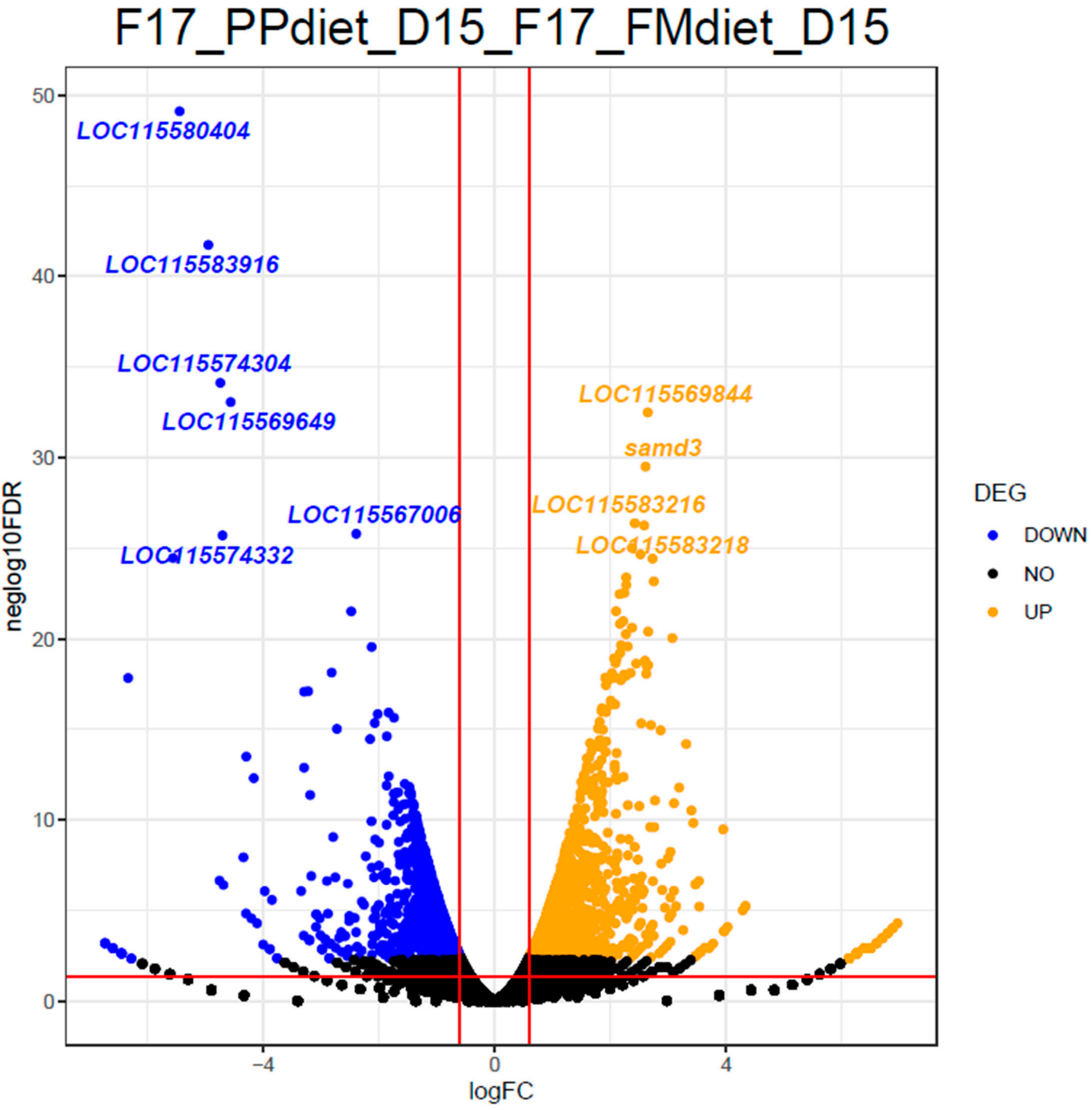

Supplementary Figure 17. Volcano plots of differentially expressed genes in F17. Condition tested: PPvsFM diets on D15. Statistically significant (p value< 0.05) down regulated DEGS in condition 2 are depicted in blue, up regulated DEGS in condition 2 are depicted in orange, and black shows non statistically significant regulated genes. The name of the top ten up and down DEGs is also given in the plot.

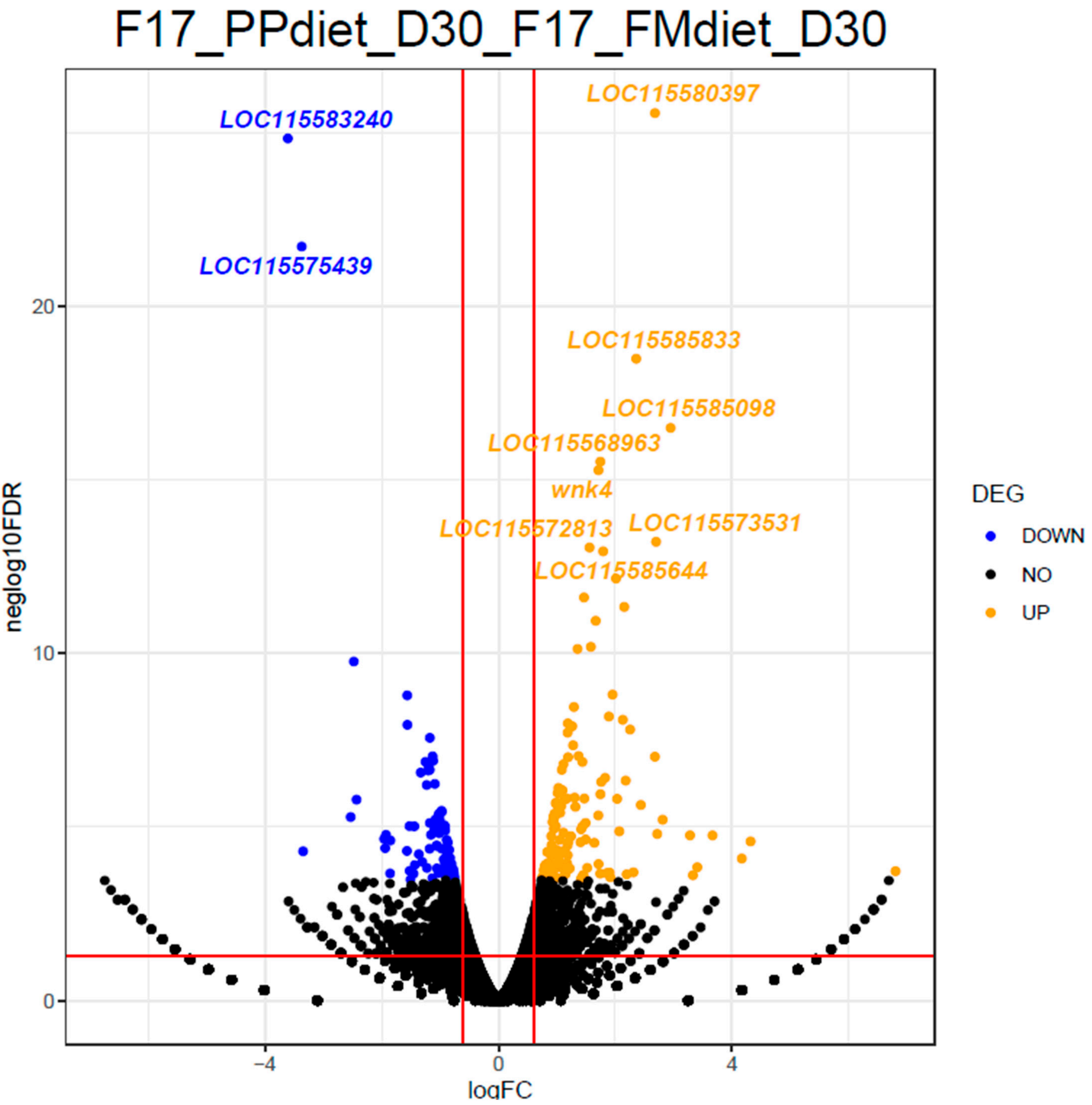

Supplementary Figure 18. Volcano plots of differentially expressed genes in F17. Condition tested: PPvsFM diets on D30. Statistically significant (p value< 0.05) down regulated DEGS in condition 2 are depicted in blue, up regulated DEGS in condition 2 are depicted in orange, and black shows non statistically significant regulated genes. The name of the top ten up and down DEGs is also given in the plot.

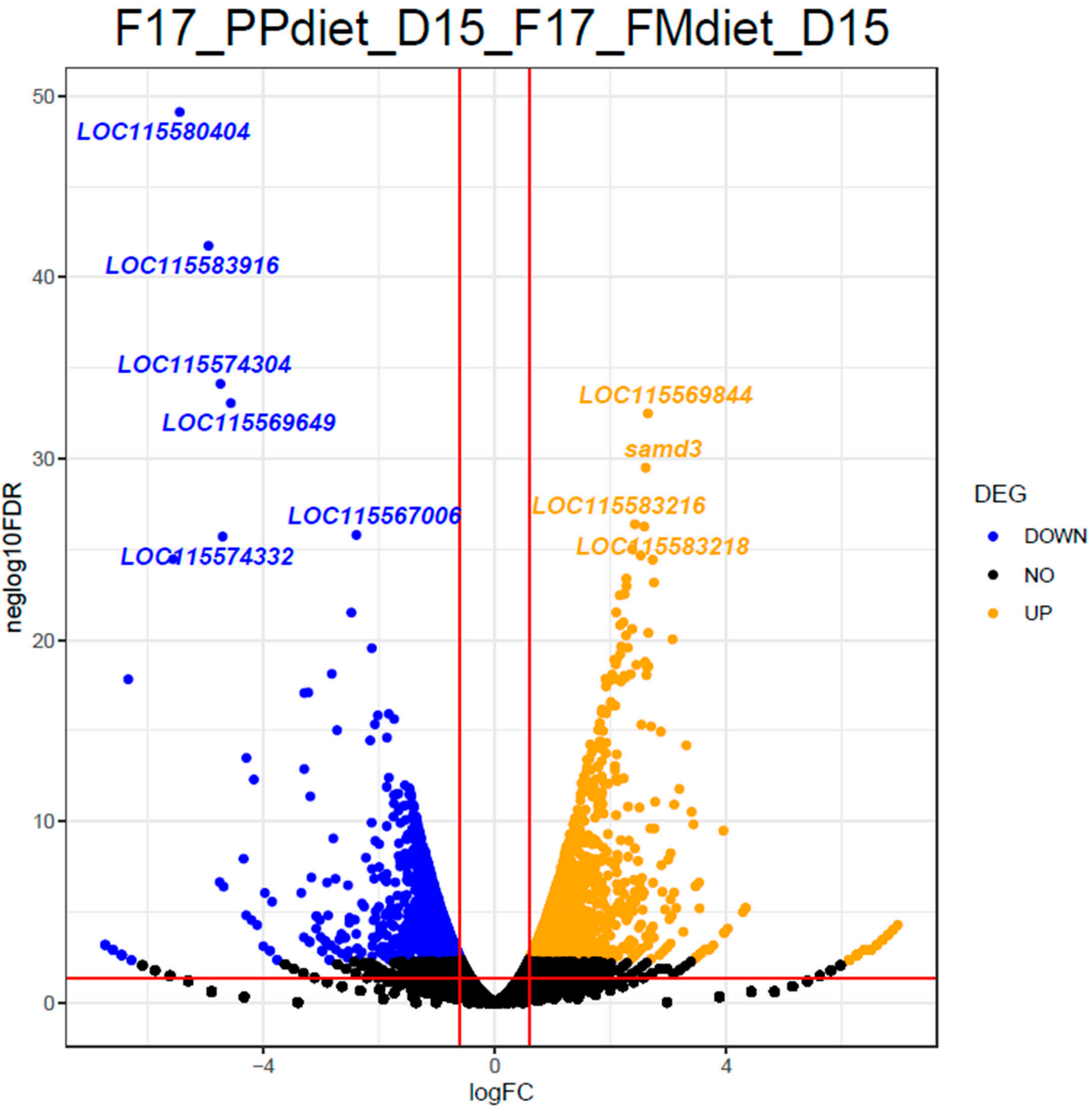

Supplementary Figure 19. Volcano plots of differentially expressed genes in F17. Condition tested: FM diet: D15 vs D30. Statistically significant (p value< 0.05) down regulated DEGS in condition 2 are depicted in blue, up regulated DEGS in condition 2 are depicted in orange, and black shows non statistically significant regulated genes. The name of the top ten up and down DEGs is also given in the plot.

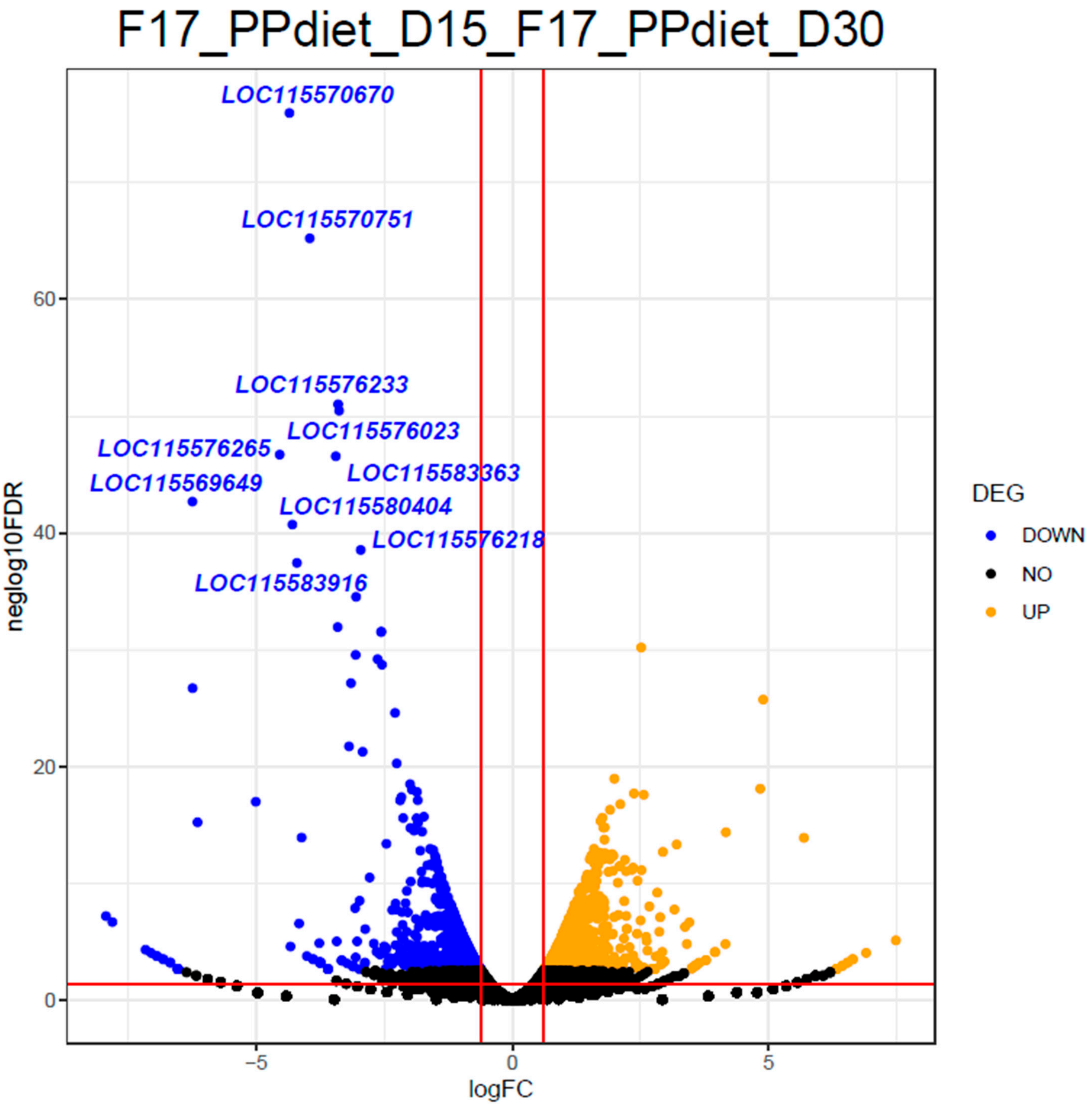

Supplementary Figure 20. Volcano plots of differentially expressed genes in F17. Condition tested: PP diet: D15 vs D30. Statistically significant (p value< 0.05) down regulated DEGS in condition 2 are depicted in blue, up regulated DEGS in condition 2 are depicted in orange, and black shows non statistically significant regulated genes. The name of the top ten up and down DEGs is also given in the plot.

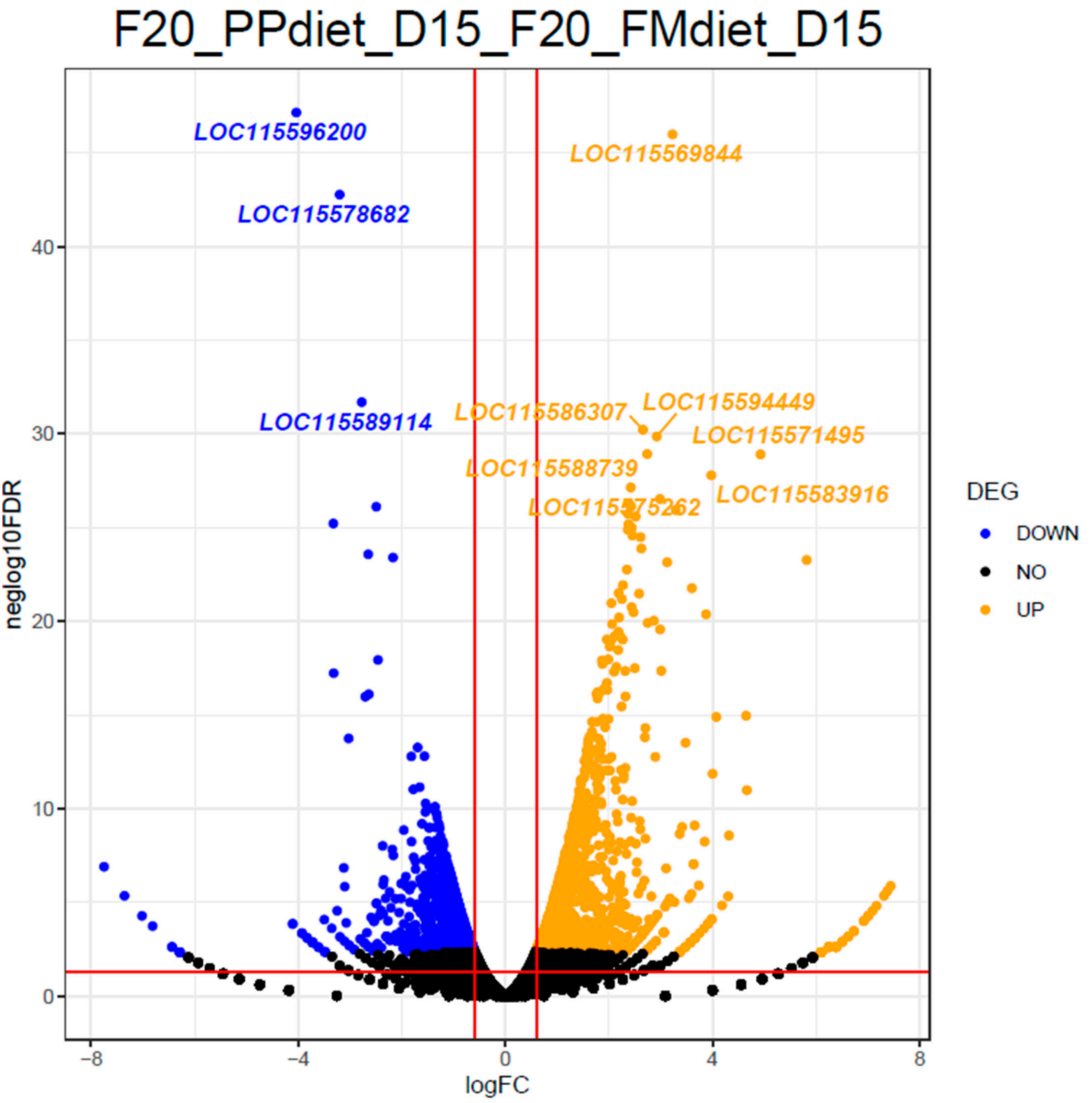

Supplementary Figure 21. Volcano plots of differentially expressed genes in F20. Condition tested: PPvsFM diets on D15. Statistically significant (p value< 0.05) down regulated DEGS in condition 2 are depicted in blue, up regulated DEGS in condition 2 are depicted in orange, and black shows non statistically significant regulated genes. The name of the top ten up and down DEGs is also given in the plot.

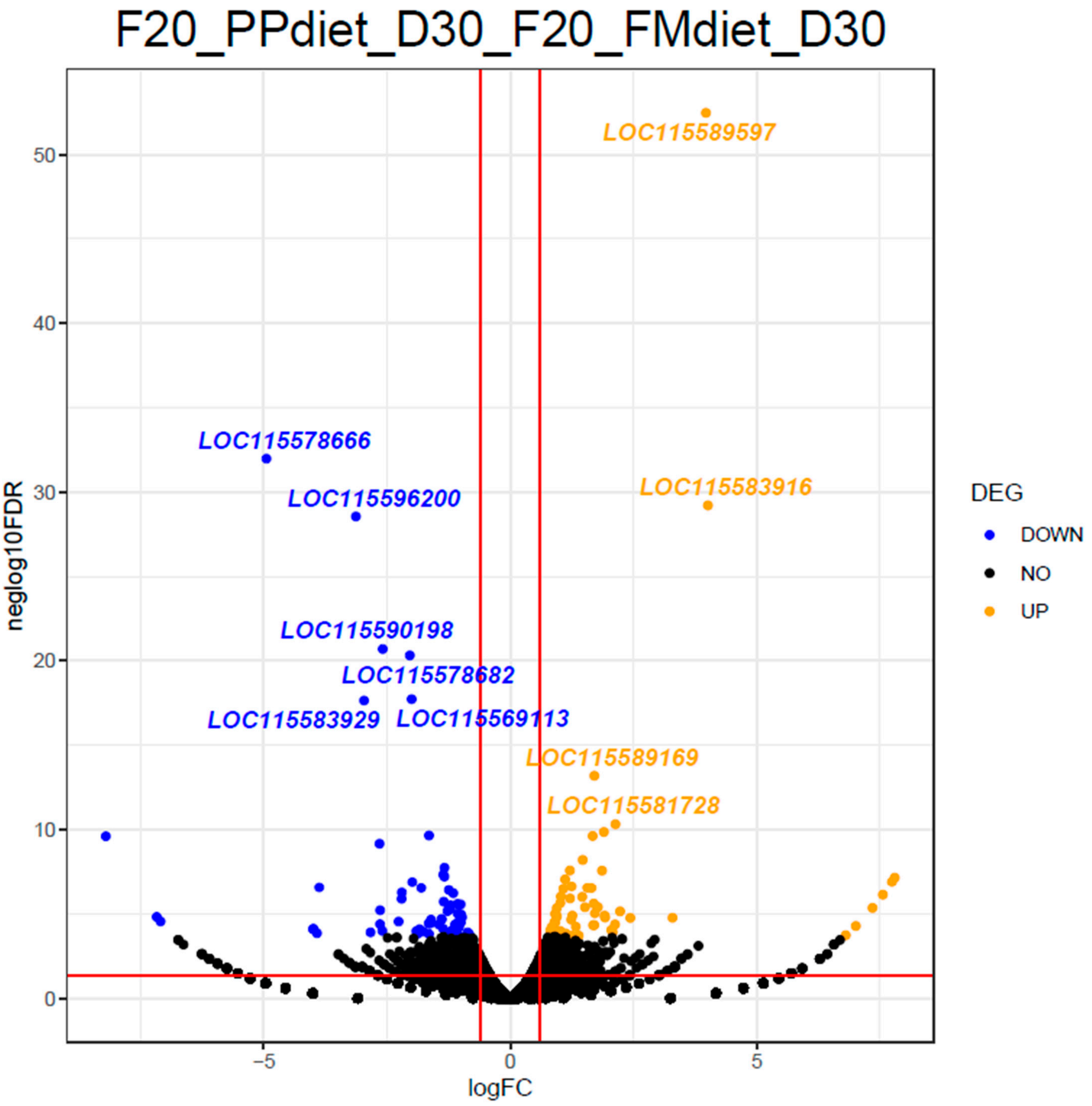

Supplementary Figure 22. Volcano plots of differentially expressed genes in F20. Condition tested: PPvsFM diets on D30. Statistically significant (p value< 0.05) down regulated DEGS in condition 2 are depicted in blue, up regulated DEGS in condition 2 are depicted in orange, and black shows non statistically significant regulated genes. The name of the top ten up and down DEGs is also given in the plot.

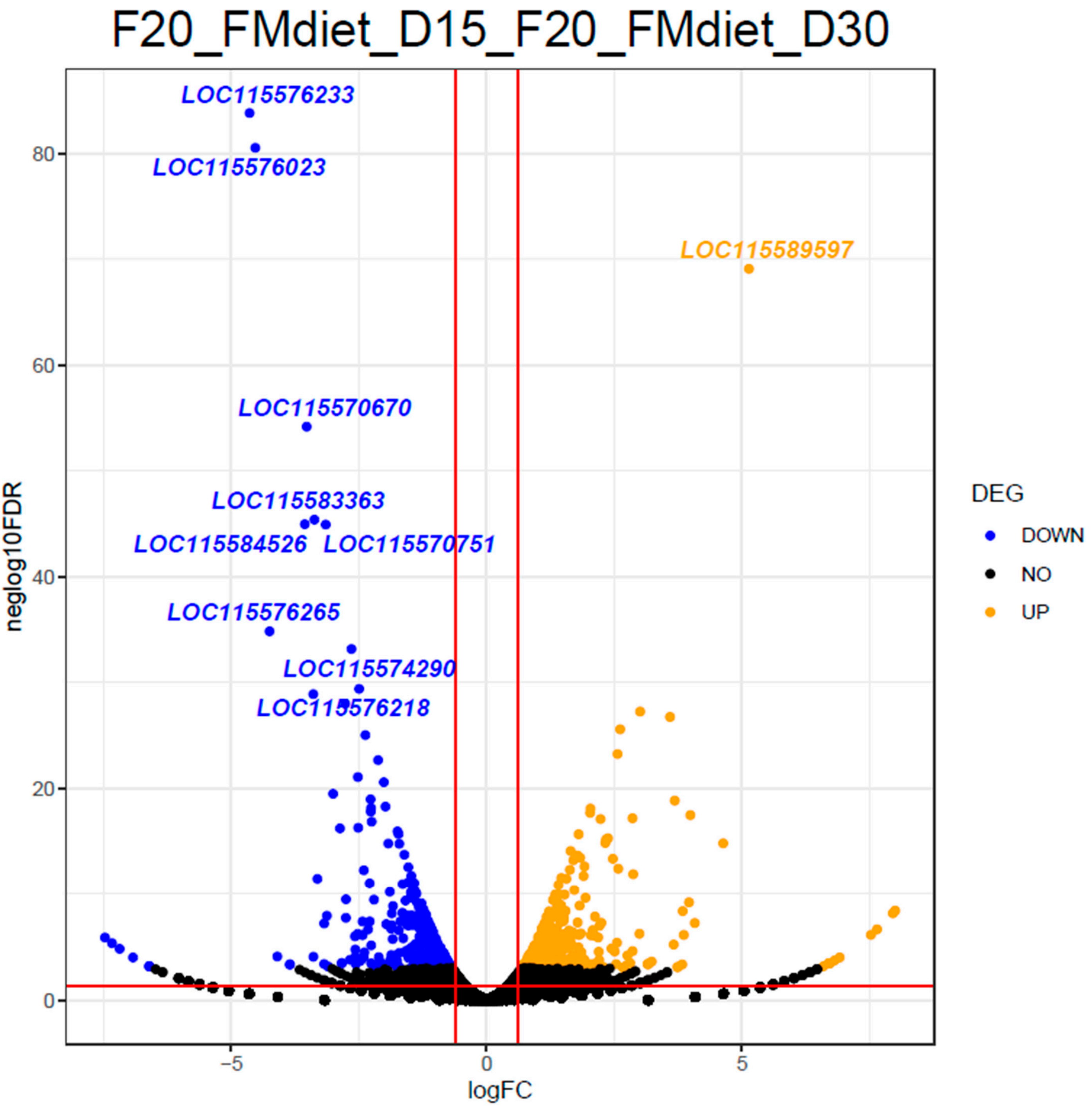

Supplementary Figure 23. Volcano plots of differentially expressed genes in F20. Condition tested: FM diet: D15 vs D30. Statistically significant (p value< 0.05) down regulated DEGS in condition 2 are depicted in blue, up regulated DEGS in condition 2 are depicted in orange, and black shows non statistically significant regulated genes. The name of the top ten up and down DEGs is also given in the plot.

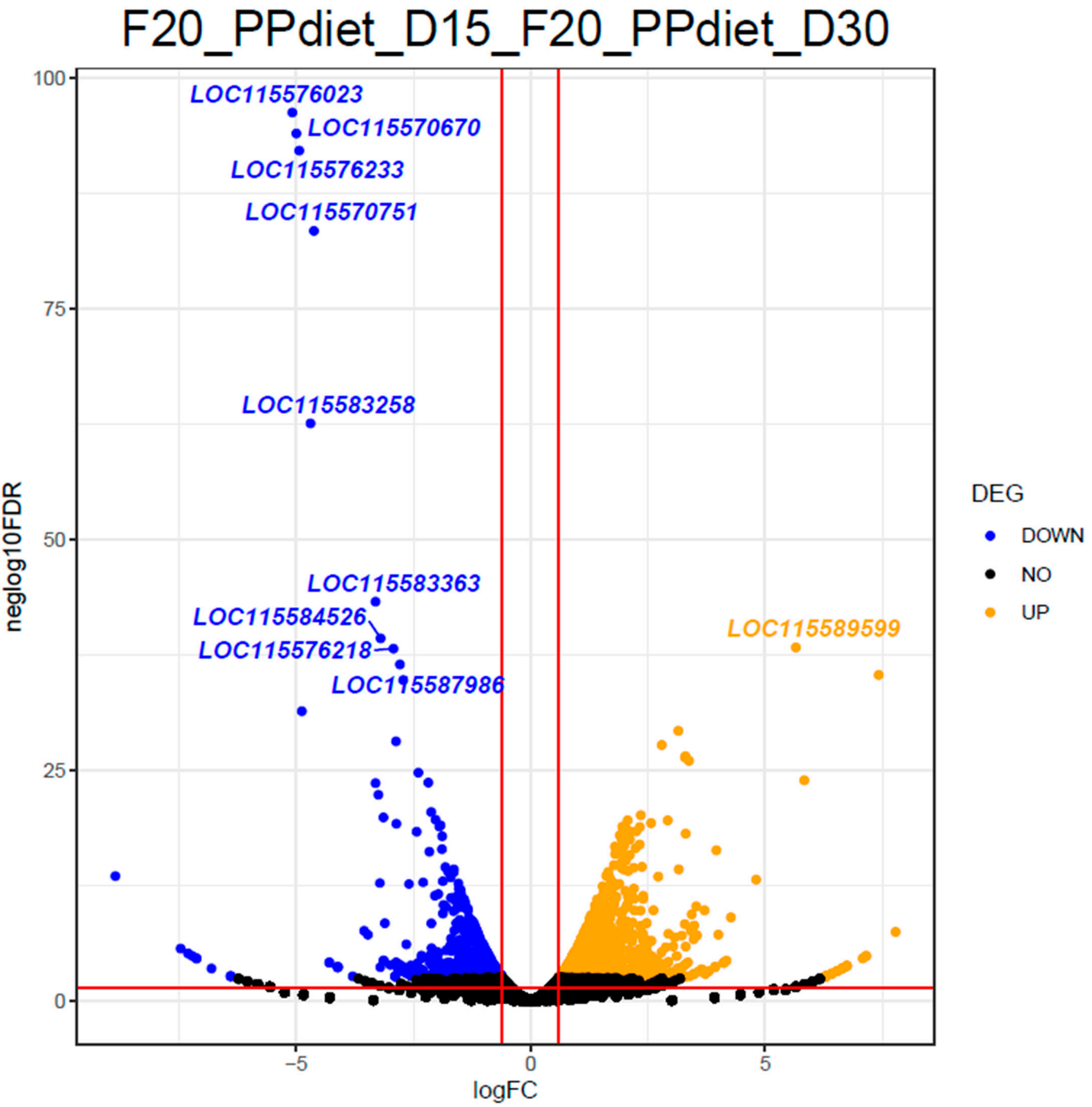

Supplementary Figure 24. Volcano plots of differentially expressed genes in F20. Condition tested: PP diet: D15 vs D30. Statistically significant (p value< 0.05) down regulated DEGS in condition 2 are depicted in blue, up regulated DEGS in condition 2 are depicted in orange, and black shows non statistically significant regulated genes. The name of the top ten up and down DEGs is also given in the plot.

Supplementary Table 1. Common oxidative phosphorylation (OxPhos) genes identified across full-sib families. The table lists OxPhos-related genes that were differentially expressed in response to the plant-protein (PP) diet and indicates the families (F5, F08, F15, F17, and F20) in which each gene was detected.

Gene	Present In
atp5f1e	F5, F08, F15, F17, F20
atp5l	F5, F08, F15, F17, F20
atp5mf	F5, F08, F15, F17, F20
cox4i2	F5, F08, F15, F17, F20
cox5b2	F5, F08, F15, F17, F20
cox7c	F5, F08, F15, F17, F20
lhpp	F5, F08, F15, F17, F20
ndufa1	F5, F08, F15, F17, F20
ndufa12	F5, F08, F15, F17, F20
ndufa5	F5, F08, F15, F17, F20
ndufa6	F5, F08, F15, F17, F20
ndufb2	F5, F08, F15, F17, F20
ndufs4	F5, F08, F15, F17, F20
ndufs6	F5, F08, F15, F17, F20
uqcr10	F5, F08, F15, F17, F20
uqcrb	F5, F08, F15, F17, F20
uqcrq	F5, F08, F15, F17, F20
atp5mc1	F5, F08, F17, F20
atp6v1h	F5, F15, F17, F20
cox5ab	F5, F08, F17, F20
cox6c	F5, F08, F17, F20
cox7a1	F5, F08, F15, F20
ndufa3	F5, F08, F15, F17
ndufb10	F5, F08, F17, F20
ndufb6	F5, F08, F17, F20
ndufb9	F5, F08, F17, F20
ndufs5	F5, F08, F17, F20
atp6v0e1	F5, F08, F17
ndufa2	F5, F08, F17
ndufs3	F5, F15, F17
sdhb	F5, F17, F20
atp5po	F5, F08
atp6v1f	F5, F20
cox6b2	F5, F20
cyc1	F15, F20
ndufa7	F5, F15
ndufb11	F5, F08
ndufb7	F5, F15
sdhc	F5, F17

Supplementary Table 2. Common Ribosome genes identified across full-sib families. The table lists Ribosome-related genes that were differentially expressed in response to the plant-protein (PP) diet and indicates the families (F5, F08, F15, F17, and F20) in which each gene was detected.

Gene	Present In	Gene	Present In
mrpl11	F05, F08, F15, F17, F20	*rps26*	F05, F08, F17, F20
mrpl18	F05, F08, F15, F17, F20	*mrpl34*	F05, F17, F20
mrps21	F05, F08, F15, F17, F20	*mrpl9*	F05, F08, F20
rpl12	F05, F08, F15, F17, F20	*rpl27*	F05, F08, F17
rpl13	F05, F08, F15, F17, F20	*rpl32*	F05, F08, F17
rpl14	F05, F08, F15, F17, F20	*rpl34*	F05, F08, F17
rpl18	F05, F08, F15, F17, F20	*rpl36a*	F05, F15, F17
rpl19	F05, F08, F15, F17, F20	*rps12*	F05, F17, F20
rpl22	F05, F08, F15, F17, F20	*rps25*	F05, F17, F20
rpl22l1	F05, F08, F15, F17, F20	*mrpl17*	F05, F15
rpl31	F05, F08, F15, F17, F20	*mrpl24*	F05, F17
rpl35a	F05, F08, F15, F17, F20	*mrpl27*	F05, F20
rpl37	F05, F08, F15, F17, F20	*mrps11*	F05, F20
rpl6	F05, F08, F15, F17, F20	*mrps9*	F05, F17
rplp1	F05, F08, F15, F17, F20	*rpl10*	F05, F17
rplp2	F05, F08, F15, F17, F20	*rpl15*	F05, F17
rps10	F05, F08, F15, F17, F20	*rpl23*	F05, F17
rps14	F05, F08, F15, F17, F20	*rpl23a*	F05, F17
rps17	F05, F08, F15, F17, F20	*rpl28*	F05, F17
rps18	F05, F08, F15, F17, F20	*rpl35*	F05, F17
rps19	F05, F08, F15, F17, F20	*rpl5b*	F05, F17
rps21	F05, F08, F15, F17, F20	*rps11*	F05, F17
rps24	F05, F08, F15, F17, F20	*rps15*	F05, F17
rps27a	F05, F08, F15, F17, F20	*rps16*	F05, F17
rps28	F05, F08, F15, F17, F20	*rps7*	F05, F17
rps3	F05, F08, F15, F17, F20	*si:dkey-159f12.2*	F05, F17
rps6	F05, F08, F15, F17, F20		
rps9	F05, F08, F15, F17, F20		
rpsa	F05, F08, F15, F17, F20		
uba52	F05, F08, F15, F17, F20		
mrpl14	F05, F08, F17, F20		
mrpl21	F05, F08, F17, F20		
rpl13a	F05, F15, F17, F20		
rpl18a	F05, F08, F15, F17		
rpl21	F05, F08, F15, F17		
rpl24	F05, F08, F15, F17		
rps15a	F05, F08, F17, F20		
rps2	F05, F08, F15, F17		

Supplementary Table 3. Ribosomal Genes Identified in E1 vs E2 Comparison. This table lists ribosomal genes identified in the comparison between experimental groups E1 and E2 across different sampling time points.

Gene	Present In	Gene	Present In	Gene	Present In
mrpl1	E1: D15, E1: D30, E2: D20	*rpl35*	E1: D15, E1: D30, E2: D20	*LOC115568713*	E1: D15, E1: D30
mrpl11	E1: D15, E1: D30, E2: D20	*rpl35a*	E1: D15, E1: D30, E2: D20	*LOC115576191*	E1: D15, E1: D30
mrpl12	E1: D15, E1: D30, E2: D20	*rpl36*	E1: D15, E1: D30, E2: D20	*LOC115577508*	E1: D15, E1: D30
mrpl18	E1: D15, E1: D30, E2: D20	*rpl36a*	E1: D15, E1: D30, E2: D20	*LOC115577589*	E1: D15, E1: D30
mrpl21	E1: D15, E1: D30, E2: D20	*rpl37*	E1: D15, E1: D30, E2: D20	*LOC115578772*	E1: D15, E1: D30
mrpl22	E1: D15, E1: D30, E2: D20	*rpl38*	E1: D15, E1: D30, E2: D20	*LOC115579752*	E1: D15, E1: D30
mrpl23	E1: D15, E1: D30, E2: D20	*rpl4*	E1: D15, E1: D30, E2: D20	*LOC115579804*	E1: D15, E1: D30
mrpl24	E1: D15, E1: D30, E2: D20	*rpl5*	E1: D15, E1: D30, E2: D20	*LOC115582713*	E1: D15, E1: D30
mrpl30	E1: D15, E1: D30, E2: D20	*rpl6*	E1: D15, E1: D30, E2: D20	*LOC115585140*	E1: D15, E1: D30
mrpl34	E1: D15, E1: D30, E2: D20	*rpl7*	E1: D15, E1: D30, E2: D20	*LOC115585953*	E1: D15, E1: D30
mrpl9	E1: D15, E1: D30, E2: D20	*rpl7a*	E1: D15, E1: D30, E2: D20	*mrpl10*	E1: D15, E1: D30
mrps11	E1: D15, E1: D30, E2: D20	*rpl8*	E1: D15, E1: D30, E2: D20	*mrpl14*	E1: D15, E2: D20
mrps15	E1: D15, E1: D30, E2: D20	*rpl9*	E1: D15, E1: D30, E2: D20	*mrpl19*	E1: D15, E1: D30
mrps6	E1: D15, E1: D30, E2: D20	*rplp0*	E1: D15, E1: D30, E2: D20	*mrpl2*	E1: D15, E1: D30
mrps9	E1: D15, E1: D30, E2: D20	*rplp1*	E1: D15, E1: D30, E2: D20	*mrpl28*	E1: D15, E1: D30
rpl10a	E1: D15, E1: D30, E2: D20	*rps10*	E1: D15, E1: D30, E2: D20	*mrpl35*	E1: D15, E1: D30
rpl11	E1: D15, E1: D30, E2: D20	*rps14*	E1: D15, E1: D30, E2: D20	*mrps10*	E1: D15, E2: D20
rpl12	E1: D15, E1: D30, E2: D20	*rps15*	E1: D15, E1: D30, E2: D20	*mrps14*	E1: D15, E1: D30
rpl13	E1: D15, E1: D30, E2: D20	*rps15a*	E1: D15, E1: D30, E2: D20	*mrps17*	E1: D15, E1: D30
rpl13a	E1: D15, E1: D30, E2: D20	*rps16*	E1: D15, E1: D30, E2: D20	*mrps18a*	E1: D15, E1: D30
rpl14	E1: D15, E1: D30, E2: D20	*rps17*	E1: D15, E1: D30, E2: D20	*mrps21*	E1: D15, E2: D20
rpl18	E1: D15, E1: D30, E2: D20	*rps2*	E1: D15, E1: D30, E2: D20	*mrps5*	E1: D30, E2: D20
rpl18a	E1: D15, E1: D30, E2: D20	*rps20*	E1: D15, E1: D30, E2: D20	*rpl10*	E1: D15, E1: D30
rpl19	E1: D15, E1: D30, E2: D20	*rps21*	E1: D15, E1: D30, E2: D20	*rpl15*	E1: D15, E1: D30
rpl21	E1: D15, E1: D30, E2: D20	*rps24*	E1: D15, E1: D30, E2: D20	*rpl37a*	E1: D15, E1: D30
rpl22	E1: D15, E1: D30, E2: D20	*rps25*	E1: D15, E1: D30, E2: D20	*rpl39*	E1: D15, E1: D30
rpl22l1	E1: D15, E1: D30, E2: D20	*rps26*	E1: D15, E1: D30, E2: D20	*rps11*	E1: D15, E1: D30
rpl23	E1: D15, E1: D30, E2: D20	*rps27a*	E1: D15, E1: D30, E2: D20	*rps13*	E1: D15, E1: D30
rpl23a	E1: D15, E1: D30, E2: D20	*rps28*	E1: D15, E1: D30, E2: D20	*rps23*	E1: D15, E1: D30
rpl24	E1: D15, E1: D30, E2: D20	*rps3*	E1: D15, E1: D30, E2: D20	*rps29*	E1: D15, E1: D30
rpl27	E1: D15, E1: D30, E2: D20	*rps3a*	E1: D15, E1: D30, E2: D20	*rps5*	E1: D15, E1: D30
rpl27a	E1: D15, E1: D30, E2: D20	*rps4x*	E1: D15, E1: D30, E2: D20	*rps8*	E1: D15, E1: D30
rpl28	E1: D15, E1: D30, E2: D20	*rps6*	E1: D15, E1: D30, E2: D20		
rpl29	E1: D15, E1: D30, E2: D20	*rps7*	E1: D15, E1: D30, E2: D20		
rpl3	E1: D15, E1: D30, E2: D20	*rps9*	E1: D15, E1: D30, E2: D20		
rpl30	E1: D15, E1: D30, E2: D20	*rsl24d1*	E1: D15, E1: D30, E2: D20		
rpl31	E1: D15, E1: D30, E2: D20	*uba52*	E1: D15, E1: D30, E2: D20		
rpl32	E1: D15, E1: D30, E2: D20	*fau*	E1: D15, E1: D30		
rpl34	E1: D15, E1: D30, E2: D20	*LOC115567885*	E1: D15, E1: D30		

Supplementary Table 4. Common Metabolic pathways related genes identified across full-sib families. The table lists Metabolic pathways-related genes that were differentially expressed in response to the plant-protein (PP) diet and indicates the families (F5, F08, F15, F17, and F20) in which each gene was detected.

Gene	Present In	Gene	Present In
alox12	F05, F08, F15, F17, F20	*h6pd*	F08, F15, F17, F20
ANPEP	F05, F08, F15, F17, F20	*hmox1a*	F08, F15, F17, F20
atp5l	F05, F08, F15, F17, F20	*kmt2ba*	F08, F15, F17, F20
atp5mf	F05, F08, F15, F17, F20	*kmt2bb*	F08, F15, F17, F20
cox4i2	F05, F08, F15, F17, F20	*kmt5c*	F08, F15, F17, F20
cox7c	F05, F08, F15, F17, F20	*mgat3b*	F08, F15, F17, F20
mthfd2	F05, F08, F15, F17, F20	*nadka*	F08, F15, F17, F20
ndufa1	F05, F08, F15, F17, F20	*ndufa12*	F08, F15, F17, F20
ndufa6	F05, F08, F15, F17, F20	*ndufa5*	F08, F15, F17, F20
ndufb2	F05, F08, F15, F17, F20	*ndufb10*	F05, F08, F17, F20
oplah	F05, F08, F15, F17, F20	*ndufb6*	F05, F08, F17, F20
pgap1	F05, F08, F15, F17, F20	*ndufs4*	F08, F15, F17, F20
plcb3	F05, F08, F15, F17, F20	*ndufs6*	F08, F15, F17, F20
ptges3a	F05, F08, F15, F17, F20	*nsd3*	F05, F08, F17, F20
sdr16c5a	F05, F08, F15, F17, F20	*nt5c2b*	F08, F15, F17, F20
si:ch211-195e19.1	F05, F08, F15, F17, F20	*nt5c2l1*	F08, F15, F17, F20
tdh	F05, F08, F15, F17, F20	*pi4kaa*	F08, F15, F17, F20
uap1l1	F05, F08, F15, F17, F20	*setd1a*	F08, F15, F17, F20
uqcr10	F05, F08, F15, F17, F20	*setd2*	F08, F15, F17, F20
uqcrq	F05, F08, F15, F17, F20	*uqcrb*	F08, F15, F17, F20
zgc:92907	F05, F08, F15, F17, F20	*acacb*	F08, F17, F20
alg13	F08, F15, F17, F20	*acsl3b*	F15, F17, F20
ash1l	F08, F15, F17, F20	*adprm*	F08, F17, F20
asl	F05, F08, F17, F20	*ahcyl2b*	F08, F15, F17
atp5f1e	F08, F15, F17, F20	*aldh6a1*	F05, F15, F20
atp5mc1	F05, F08, F17, F20	*alpl*	F05, F08, F20
atp6v1h	F05, F15, F17, F20	*arg2*	F05, F17, F20
cdab	F05, F08, F15, F20	*chdh*	F05, F08, F17
cerk	F08, F15, F17, F20	*ckba*	F08, F17, F20
ckbb	F05, F08, F17, F20	*coq2*	F05, F15, F20
cox5ab	F05, F08, F17, F20	*cox6c*	F08, F17, F20
cox5b2	F08, F15, F17, F20	*cox7a1*	F08, F15, F20
cps1	F08, F15, F17, F20	*cyp3a65*	F08, F15, F17
dot1l	F08, F15, F17, F20	*dctpp1*	F08, F17, F20
eprs1	F08, F15, F17, F20	*DGKI*	F05, F08, F20
etnk2	F08, F15, F17, F20	*entpd4*	F05, F17, F20
ezh1	F08, F15, F17, F20	*extl3*	F08, F17, F20
fech	F08, F15, F17, F20	*idi1*	F08, F15, F20
gnpnat1	F08, F15, F17, F20	*impa1*	F05, F17, F20
gnsa	F08, F15, F17, F20	*man1b1b*	F08, F17, F20
mdh1aa	F05, F08, F15	*hacd3*	F08, F20
mmab	F08, F17, F20	*hkdc1*	F05, F20
ndufa3	F08, F15, F17	*kl*	F08, F15
ndufb9	F08, F17, F20	*lipt2*	F17, F20

ndufs5	F08, F17, F20
nfs1	F08, F15, F17
nit2	F05, F15, F20
p4ha1a	F08, F17, F20
phykpl	F08, F15, F17
pik3c2a	F08, F15, F20
pik3ca	F08, F15, F20
pmvk	F05, F15, F20
tusc3	F08, F15, F17
uap1	F05, F17, F20
xylt2	F08, F15, F20
aco2	F15, F20
acsbg1	F05, F15
ahcy	F05, F15
aldob	F15, F20
alg12	F17, F20
arg1	F17, F20
atp5po	F05, F08
atp6v0e1	F08, F17
atp6v1f	F05, F20
blvrb	F05, F20
ca2	F15, F17
cdo1	F08, F17
chka	F08, F17
cox6b2	F05, F20
cyc1	F15, F20
dpm3	F05, F08
ehmt1a	F05, F17
enpp1	F17, F20
etnk1	F15, F20
fdps	F17, F20
gatc	F08, F20
glyctk	F08, F17
gstm.1	F08, F17
gstm.2	F08, F17
gstm.3	F08, F17
lpin2	F17, F20
man1a1	F08, F15
mocs1	F08, F15
msmo1	F08, F20
mthfs	F05, F17
mtmr1a	F17, F20
mtmr7b	F08, F15
mtr	F08, F17
naprt	F08, F20
ndufa2	F08, F17
ndufa7	F05, F15
ndufb7	F05, F15
ndufs3	F15, F17
neu1	F17, F20
nsd1b	F17, F20
paics	F08, F15
pdxp	F15, F20
pfkfb3	F08, F20
phgdh	F17, F20
pik3cd	F17, F20
plod3	F08, F15
ppox	F15, F20
ppt2	F05, F17
sardh	F05, F17
sdhb	F17, F20
setmar	F15, F20
srd5a1	F15, F20
tecra	F15, F17
tecrb	F15, F17
uros	F15, F20

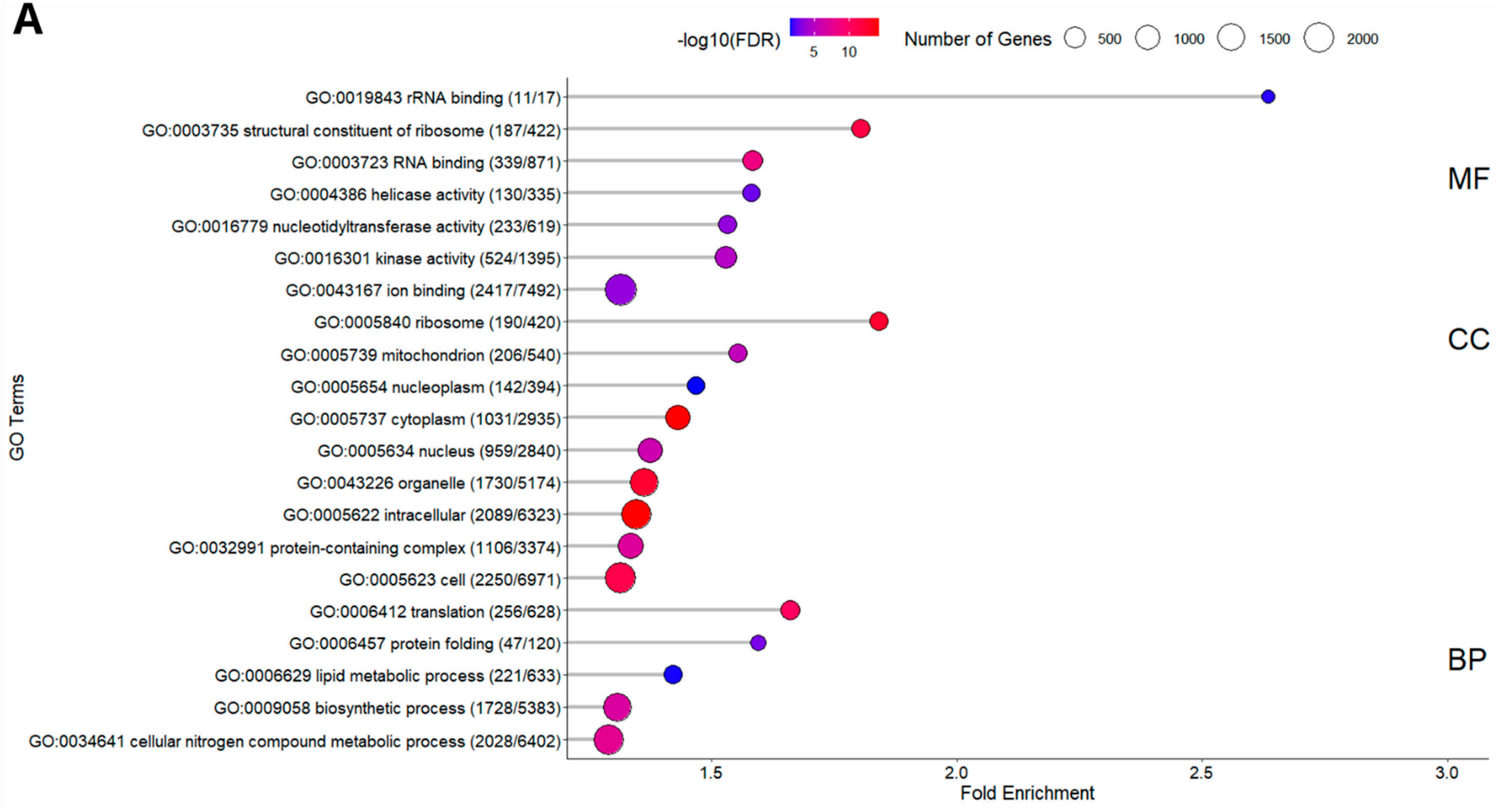
A
-log10(FDR)
5
10
Number of Genes
500
1000
1500
2000
GO:0019843 rRNA binding (11/17)
GO:0003735 structural constituent of ribosome (187/422)
GO:0003723 RNA binding (339/871)
GO:0004386 helicase activity (130/335)
GO:0016779 nucleotidyltransferase activity (233/619)
GO:0016301 kinase activity (524/1395)
GO:0043167 ion binding (2417/7492)
GO:0005840 ribosome (190/420)
GO:0005739 mitochondrion (206/540)
GO:0005654 nucleoplasm (142/394)
GO:0005737 cytoplasm (1031/2935)
GO:0005634 nucleus (959/2840)
GO:0043226 organelle (1730/5174)
GO:0005622 intracellular (2089/6323)
GO:0032991 protein-containing complex (1106/3374)
GO:0005623 cell (2250/6971)
GO:0006412 translation (256/628)
GO:0006457 protein folding (47/120)
GO:0006629 lipid metabolic process (221/633)
GO:0009058 biosynthetic process (1728/5383)
GO:0034641 cellular nitrogen compound metabolic process (2028/6402)
GO Terms
MF
CC
BP
1.5
2.0
2.5
3.0
Fold Enrichment

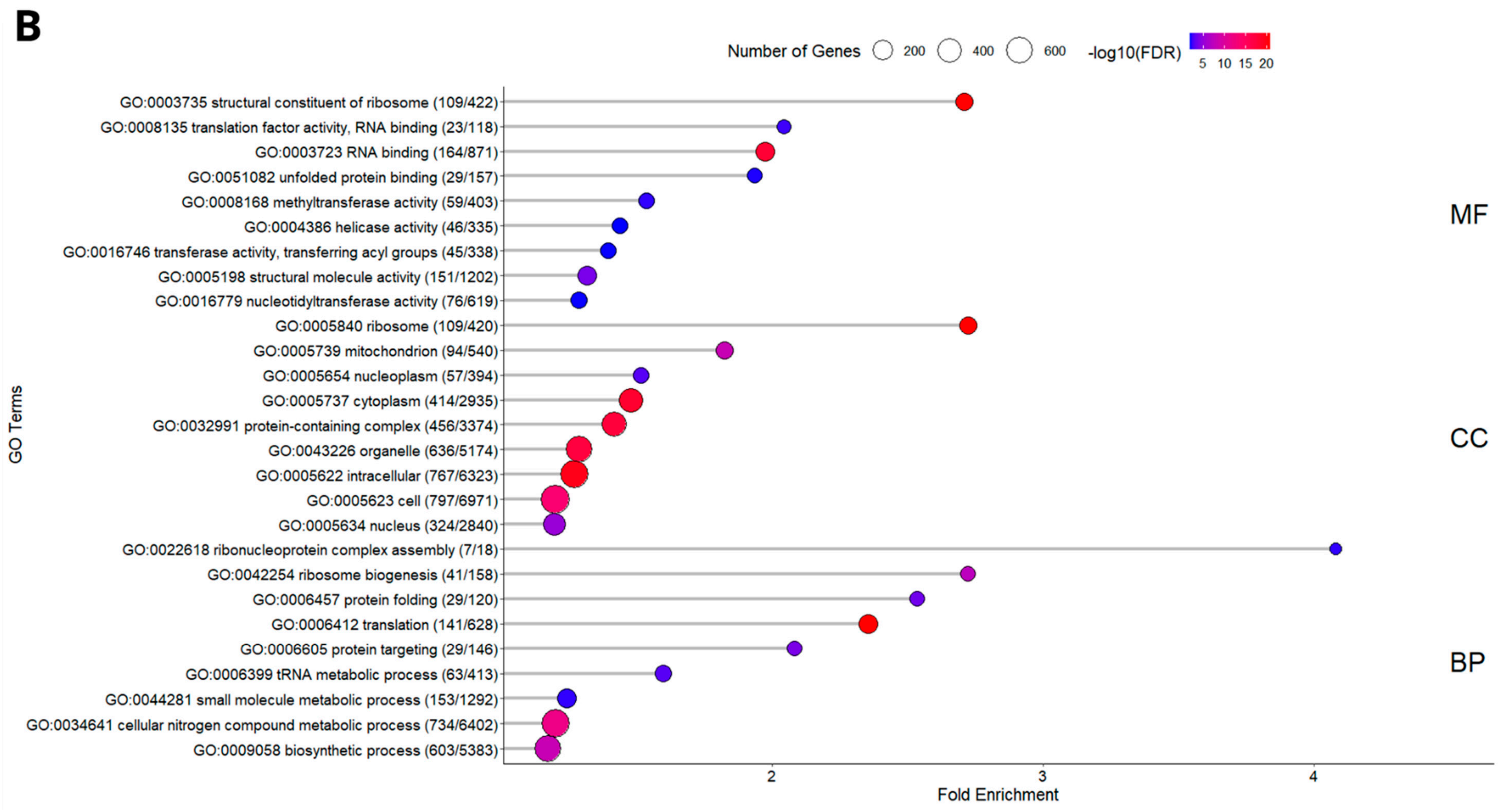

Supplementary Figure 25. Go term enrichment analysis of differentially expressed proteins in the three GO annotation domains: Biological Processes (BP), Cellular Components (CC), and Molecular Functions (MF) E1: A: FM vs PP D15, B: FM vs PP D30. The x-axis represents the fold enrichment, indicating the magnitude of overrepresentation of GO terms in the dataset compared to the background. The y-axis lists the enriched GO terms, ordered by fold enrichment within each category. The size of the circles corresponds to the number of differentially expressed proteins associated with each GO term, with larger circles representing GO terms involving more proteins. The color gradient of the circles, ranging from blue to red, reflects the significance of enrichment as measured by the -log10(FDR) value, with red representing more significant enrichment.

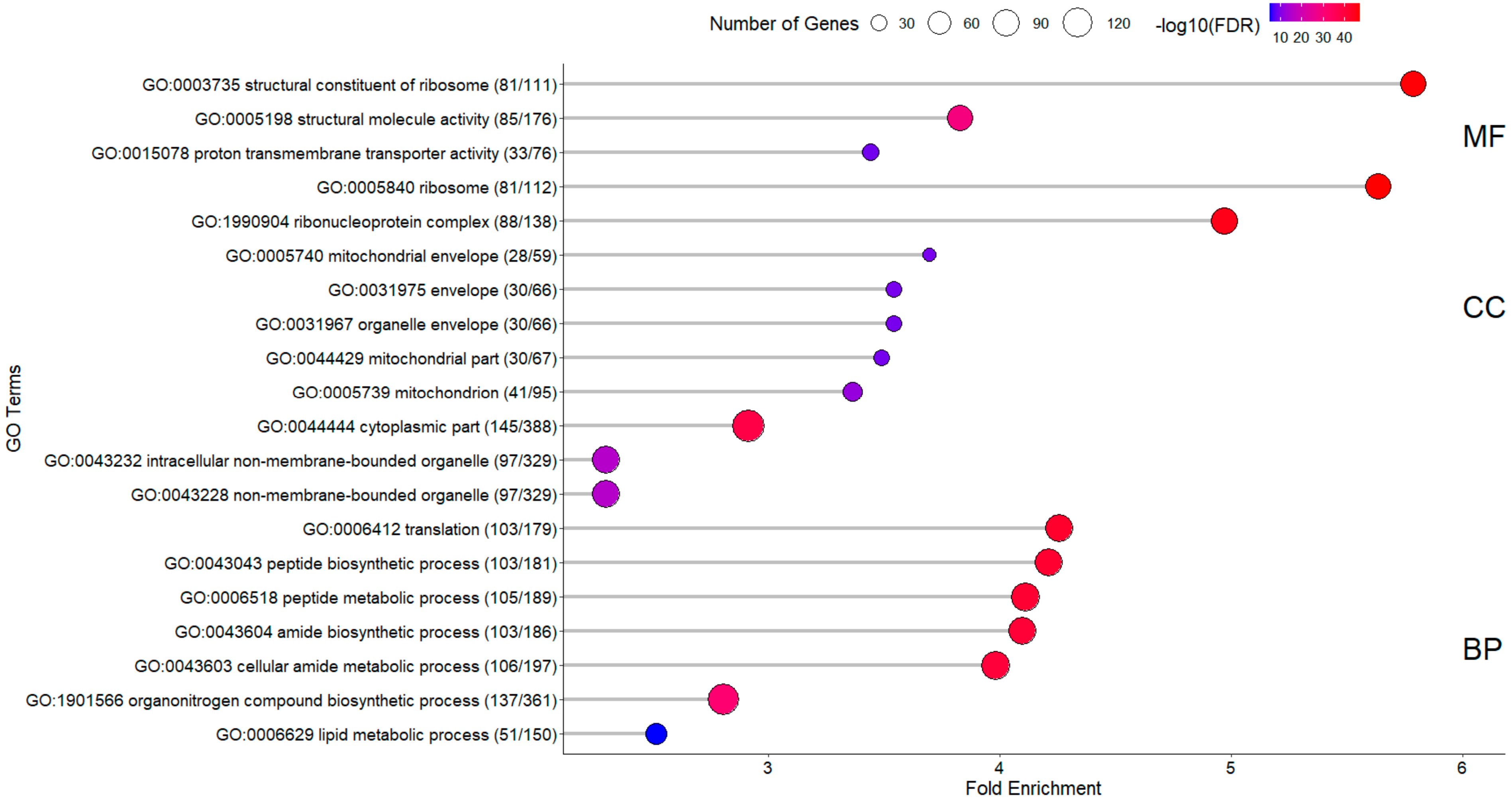

Supplementary Figure 26. Go term enrichment analysis of differentially expressed proteins in the three GO annotation domains: Biological Processes (BP), Cellular Components (CC), and Molecular Functions (MF) E2: FM vs PP. The x-axis represents the fold enrichment, indicating the magnitude of overrepresentation of GO terms in the dataset compared to the background. The y-axis lists the enriched GO terms, ordered by fold enrichment within each category. The size of the circles corresponds to the number of differentially expressed proteins associated with each GO term, with larger circles representing GO terms involving more proteins. The color gradient of the circles, ranging from blue to red, reflects the significance of enrichment as measured by the -log10(FDR) value, with red representing more significant enrichment.

Supplementary Table 5. DEGs for key erythrocyte-associated genes

Genes	logFC	PValue	FDR	DEG	Description	Comparisons
ca5a	1.488358743	6.58587E-11	2.52458E-10	UP	carbonic anhydrase 5A [Source:NCBI gene;Acc:115586666]	**F05_PPdiet_D15_F05_PPdiet_D30**
ca6	0.904373512	5.0833E-05	7.79439E-05	UP	carbonic anhydrase 6 [Source:NCBI gene;Acc:115584806]	
LOC115568977	1.257612455	5.23457E-07	1.33772E-06	UP	sodium bicarbonate transporter-like protein 11 [Source:NCBI gene;Acc:115568977]	
LOC115570135	1.256573475	1.4113E-09	4.63712E-09	UP	carbonic anhydrase 1-like [Source:NCBI gene;Acc:115570135]	
LOC115570185	2.641464032	1.36697E-05	2.24574E-05	UP	anion exchange protein 2-like [Source:NCBI gene;Acc:115570185]	
LOC115570670	-3.637727596	6.45692E-57	1.48509E-55	DOWN	hemoglobin subunit alpha-1-like [Source:NCBI gene;Acc:115570670]	
LOC115570751	-2.200659921	1.16838E-24	1.34363E-23	DOWN	hemoglobin subunit beta-1-like [Source:NCBI gene;Acc:115570751]	
LOC115576210	1.042550793	0.030066952	0.040678818	UP	hemoglobin subunit beta-like [Source:NCBI gene;Acc:115575823]	
LOC115576212	-1.207663855	7.35372E-07	1.69136E-06	DOWN	hemoglobin subunit beta-2-like [Source:NCBI gene;Acc:115576212]	
LOC115576214	0.789437694	0.000128147	0.000184212	UP	hemoglobin cathodic subunit beta-like [Source:NCBI gene;Acc:115576214]	
LOC115576218	-1.538737808	7.80585E-12	3.59069E-11	DOWN	hemoglobin subunit alpha-1 [Source:NCBI gene;Acc:115576218]	
LOC115576321	1.484368855	1.14905E-12	6.60705E-12	UP	band 3 anion exchange protein-like [Source:NCBI gene;Acc:115576321]	
LOC115578684	1.786851397	2.26592E-07	6.51453E-07	UP	sodium bicarbonate cotransporter 3-like [Source:NCBI gene;Acc:115578684]	
LOC115588298	1.194247128	9.25094E-07	1.93429E-06	UP	glucose-6-phosphate exchanger SLC37A1-like [Source:NCBI gene;Acc:115588298]	
LOC115592532	2.556156844	2.04767E-06	3.9247E-06	UP	electrogenic sodium bicarbonate cotransporter 4-like [Source:NCBI gene;Acc:115592532]	
slc26a5	1.091210167	5.6564E-06	1.00075E-05	UP	solute carrier family 26 member 5 [Source:NCBI gene;Acc:115595297]	
slc4a11	2.409381355	8.96873E-14	6.87602E-13	UP	solute carrier family 4 member 11 [Source:NCBI gene;Acc:115597494]	
ca5a	0.946262815	3.30642E-05	0.00010864	UP	carbonic anhydrase 5A [Source:NCBI gene;Acc:115586666]	**F05_PPdiet_D15_F05_FMdiet_D15**
LOC115570185	1.851423862	0.00644454	0.014822441	UP	anion exchange protein 2-like [Source:NCBI gene;Acc:115570185]	
LOC115570670	-0.810658373	8.69818E-05	0.000250073	DOWN	hemoglobin subunit alpha-1-like [Source:NCBI gene;Acc:115570670]	
LOC115570751	-1.221365049	4.37912E-09	3.35733E-08	DOWN	hemoglobin subunit beta-1-like [Source:NCBI gene;Acc:115570751]	
LOC115576321	1.436584435	5.5263E-12	1.27105E-10	UP	band 3 anion exchange protein-like [Source:NCBI gene;Acc:115576321]	
LOC115578684	1.478282698	1.44345E-05	5.53323E-05	UP	sodium bicarbonate cotransporter 3-like [Source:NCBI gene;Acc:115578684]	
LOC115592532	2.896260965	3.7454E-09	3.35733E-08	UP	electrogenic sodium bicarbonate cotransporter 4-like [Source:NCBI gene;Acc:115592532]	
LOC115594511	2.247462703	2.71595E-07	1.24934E-06	UP	carbonic anhydrase 4-like [Source:NCBI gene;Acc:115594511]	
slc26a5	0.688970257	0.0046416	0.011861866	UP	solute carrier family 26 member 5 [Source:NCBI gene;Acc:115595297]	
slc4a11	1.78698713	7.44757E-08	4.28235E-07	UP	solute carrier family 4 member 11 [Source:NCBI gene;Acc:115597494]	
LOC115568977	-0.881928149	0.00046115	0.005303223	DOWN	sodium bicarbonate transporter-like protein 11 [Source:NCBI gene;Acc:115568977]	**F05_PPdiet_D30_F05_FMdiet_D30**
LOC115570135	-0.754096882	0.000244284	0.005303223	DOWN	carbonic anhydrase 1-like [Source:NCBI gene;Acc:115570135]	

LOC115570751	-0.68248834	0.001117766	0.008569538	DOWN	hemoglobin subunit beta-1-like [Source:NCBI gene;Acc:115570751]	
LOC115570670	-2.790100586	1.77136E-36	4.07412E-35	DOWN	hemoglobin subunit alpha-1-like [Source:NCBI gene;Acc:115570670]	F05_FMdiet_D15_F05_FMdiet_D30
LOC115570751	-1.722057488	5.29884E-16	6.09366E-15	DOWN	hemoglobin subunit beta-1-like [Source:NCBI gene;Acc:115570751]	
LOC115576212	-1.340910761	5.84046E-08	3.35827E-07	DOWN	hemoglobin subunit beta-2-like [Source:NCBI gene;Acc:115576212]	
LOC115576218	-1.686166784	2.82087E-13	2.16267E-12	DOWN	hemoglobin subunit alpha-1 [Source:NCBI gene;Acc:115576218]	
ca5a	1.57086527	6.21425E-12	3.57319E-11	UP	carbonic anhydrase 5A [Source:NCBI gene;Acc:115586666]	F06_PPdiet_D15_F06_PPdiet_D30
LOC115568977	1.03936128	0.000547773	0.001145344	UP	sodium bicarbonate transporter-like protein 11 [Source:NCBI gene;Acc:115568977]	
LOC115570135	1.500376901	6.90703E-13	5.29539E-12	UP	carbonic anhydrase 1-like [Source:NCBI gene;Acc:115570135]	
LOC115570185	1.587981239	0.012098155	0.018550505	UP	anion exchange protein 2-like [Source:NCBI gene;Acc:115570185]	
LOC115570654	0.608026902	0.003070575	0.00588527	UP	hemoglobin subunit alpha-D-like [Source:NCBI gene;Acc:115570654]	
LOC115570670	-3.384245954	2.25001E-50	5.17502E-49	DOWN	hemoglobin subunit alpha-1-like [Source:NCBI gene;Acc:115570670]	
LOC115570751	-2.733725379	2.77799E-35	3.19469E-34	DOWN	hemoglobin subunit beta-1-like [Source:NCBI gene;Acc:115570751]	
LOC115576210	-3.04458333	0.026780481	0.036232416	DOWN	hemoglobin subunit beta-like [Source:NCBI gene;Acc:115575823]	
LOC115576212	-1.082839441	8.48993E-06	2.16965E-05	DOWN	hemoglobin subunit beta-2-like [Source:NCBI gene;Acc:115576212]	
LOC115576214	0.87896168	2.04265E-05	4.6981E-05	UP	hemoglobin cathodic subunit beta-like [Source:NCBI gene;Acc:115576214]	
LOC115576218	-1.181516926	5.89734E-08	1.9377E-07	DOWN	hemoglobin subunit alpha-1 [Source:NCBI gene;Acc:115576218]	
LOC115576321	1.201825972	6.67608E-09	2.55916E-08	UP	band 3 anion exchange protein-like [Source:NCBI gene;Acc:115576321]	
LOC115578684	0.951377017	0.004728708	0.008366176	UP	sodium bicarbonate cotransporter 3-like [Source:NCBI gene;Acc:115578684]	
LOC115588298	1.457036606	2.27495E-09	1.04648E-08	UP	glucose-6-phosphate exchanger SLC37A1-like [Source:NCBI gene;Acc:115588298]	
LOC115592532	-2.670928225	0.024634949	0.03541274	DOWN	electrogenic sodium bicarbonate cotransporter 4-like [Source:NCBI gene;Acc:115592532]	
slc26a5	1.250588346	2.17013E-07	6.23914E-07	UP	solute carrier family 26 member 5 [Source:NCBI gene;Acc:115595297]	
slc4a11	0.648990223	0.03343248	0.04271928	UP	solute carrier family 4 member 11 [Source:NCBI gene;Acc:115597494]	
ca5a	0.957245055	2.70909E-05	0.000155773	UP	carbonic anhydrase 5A [Source:NCBI gene;Acc:115586666]	F06_PPdiet_D15_F06_FMdiet_D15
LOC115568977	0.971745959	0.000874149	0.003350906	UP	sodium bicarbonate transporter-like protein 11 [Source:NCBI gene;Acc:115568977]	
LOC115570135	1.047844222	4.00543E-07	3.07083E-06	UP	carbonic anhydrase 1-like [Source:NCBI gene;Acc:115570135]	
LOC115570654	0.631497608	0.002107223	0.005732475	UP	hemoglobin subunit alpha-D-like [Source:NCBI gene;Acc:115570654]	
LOC115576214	0.628193448	0.002243143	0.005732475	UP	hemoglobin cathodic subunit beta-like [Source:NCBI gene;Acc:115576214]	
LOC115576321	1.341173605	1.11603E-10	2.56687E-09	UP	band 3 anion exchange protein-like [Source:NCBI gene;Acc:115576321]	
LOC115578684	1.02744069	0.001237909	0.004067415	UP	sodium bicarbonate cotransporter 3-like [Source:NCBI gene;Acc:115578684]	
LOC115588298	1.252394912	2.12432E-07	2.44297E-06	UP	glucose-6-phosphate exchanger SLC37A1-like [Source:NCBI gene;Acc:115588298]	
slc26a5	0.827609164	0.000625577	0.002877654	UP	solute carrier family 26 member 5 [Source:NCBI gene;Acc:115595297]	

slc4a11	0.750099448	0.010572618	0.024317021	UP	solute carrier family 4 member 11 [Source:NCBI gene;Acc:115597494]	
LOC115576210	3.738970582	0.000794618	0.018276209	UP	hemoglobin subunit beta-like [Source:NCBI gene;Acc:115575823]	**F06_PPdiet_D30_F06_FMdiet_D30**
LOC115570185	2.084454553	0.00016937	0.000779104	UP	anion exchange protein 2-like [Source:NCBI gene;Acc:115570185]	
LOC115570670	-3.285264632	4.18252E-47	9.6198E-46	DOWN	hemoglobin subunit alpha-1-like [Source:NCBI gene;Acc:115570670]	
LOC115570751	-2.597139137	2.96432E-32	3.40896E-31	DOWN	hemoglobin subunit beta-1-like [Source:NCBI gene;Acc:115570751]	**F06_FMdiet_D15_F06_FMdiet_D30**
LOC115576210	1.968396104	0.010399363	0.034169335	UP	hemoglobin subunit beta-like [Source:NCBI gene;Acc:115575823]	
LOC115576212	-1.857800214	1.69196E-13	1.29717E-12	DOWN	hemoglobin subunit beta-2-like [Source:NCBI gene;Acc:115576212]	
LOC115576218	-1.165559063	9.85637E-08	5.66741E-07	DOWN	hemoglobin subunit alpha-1 [Source:NCBI gene;Acc:115576218]	
ca5a	1.543104525	1.17608E-10	4.5083E-10	UP	carbonic anhydrase 5A [Source:NCBI gene;Acc:115586666]	
LOC115568977	1.407492962	2.44894E-07	5.12052E-07	UP	sodium bicarbonate transporter-like protein 11 [Source:NCBI gene;Acc:115568977]	
LOC115570135	1.257700975	1.37685E-09	3.51861E-09	UP	carbonic anhydrase 1-like [Source:NCBI gene;Acc:115570135]	
LOC115570185	1.924870754	5.99083E-10	1.72236E-09	UP	anion exchange protein 2-like [Source:NCBI gene;Acc:115570185]	
LOC115570670	-3.60655129	9.98279E-57	2.29604E-55	DOWN	hemoglobin subunit alpha-1-like [Source:NCBI gene;Acc:115570670]	
LOC115570751	-3.080703122	9.70609E-44	1.1162E-42	DOWN	hemoglobin subunit beta-1-like [Source:NCBI gene;Acc:115570751]	
LOC115576212	-1.530751415	3.95692E-11	1.82018E-10	DOWN	hemoglobin subunit beta-2-like [Source:NCBI gene;Acc:115576212]	
LOC115576214	0.841162273	4.4873E-05	8.60066E-05	UP	hemoglobin cathodic subunit beta-like [Source:NCBI gene;Acc:115576214]	**F08_PPdiet_D15_F08_PPdiet_D30**
LOC115576218	-1.646557821	2.88002E-14	2.20801E-13	DOWN	hemoglobin subunit alpha-1 [Source:NCBI gene;Acc:115576218]	
LOC115576321	1.297255823	4.23456E-10	1.39135E-09	UP	band 3 anion exchange protein-like [Source:NCBI gene;Acc:115576321]	
LOC115578684	1.892782229	5.88811E-09	1.35427E-08	UP	sodium bicarbonate cotransporter 3-like [Source:NCBI gene;Acc:115578684]	
LOC115588298	1.714072985	1.44787E-11	8.32523E-11	UP	glucose-6-phosphate exchanger SLC37A1-like [Source:NCBI gene;Acc:115588298]	
LOC115592532	2.21758544	0.006647239	0.010192432	UP	electrogenic sodium bicarbonate cotransporter 4-like [Source:NCBI gene;Acc:115592532]	
slc26a5	0.85809085	0.000329249	0.000582518	UP	solute carrier family 26 member 5 [Source:NCBI gene;Acc:115595297]	
slc37a2	0.996031587	0.034093522	0.049009438	UP	solute carrier family 37 member 2 [Source:NCBI gene;Acc:115594661]	
slc4a11	0.925607763	0.002379554	0.003909268	UP	solute carrier family 4 member 11 [Source:NCBI gene;Acc:115597494]	
ca5a	0.825622908	0.000882802	0.002900634	UP	carbonic anhydrase 5A [Source:NCBI gene;Acc:115586666]	
LOC115570185	1.564415798	2.6042E-06	1.99655E-05	UP	anion exchange protein 2-like [Source:NCBI gene;Acc:115570185]	
LOC115576214	0.717914192	0.000486046	0.001863175	UP	hemoglobin cathodic subunit beta-like [Source:NCBI gene;Acc:115576214]	**F08_PPdiet_D15_F08_FMdiet_D15**
LOC115576321	1.354730235	7.39203E-11	1.70017E-09	UP	band 3 anion exchange protein-like [Source:NCBI gene;Acc:115576321]	
LOC115578684	1.437556198	2.86366E-05	0.000131729	UP	sodium bicarbonate cotransporter 3-like [Source:NCBI gene;Acc:115578684]	

LOC115588298	1.545382535	2.69798E-09	3.10268E-08	UP	glucose-6-phosphate exchanger SLC37A1-like [Source:NCBI gene;Acc:115588298]	
slc26a5	0.605810793	0.01397045	0.029210941	UP	solute carrier family 26 member 5 [Source:NCBI gene;Acc:115595297]	
slc4a11	1.256770174	2.85756E-05	0.000131729	UP	solute carrier family 4 member 11 [Source:NCBI gene;Acc:115597494]	
LOC115568977	-0.991107685	0.000212842	0.002447682	DOWN	sodium bicarbonate transporter-like protein 11 [Source:NCBI gene;Acc:115568977]	**F08_PPdiet_D30_F08_FMdiet_D30**
LOC115570135	-0.856808238	3.17587E-05	0.00073045	DOWN	carbonic anhydrase 1-like [Source:NCBI gene;Acc:115570135]	
LOC115570185	0.817369612	0.002588987	0.01190934	UP	anion exchange protein 2-like [Source:NCBI gene;Acc:115570185]	
LOC115570670	-3.000587736	8.50638E-42	1.95647E-40	DOWN	hemoglobin subunit alpha-1-like [Source:NCBI gene;Acc:115570670]	
LOC115570751	-2.614620288	4.40057E-33	5.06065E-32	DOWN	hemoglobin subunit beta-1-like [Source:NCBI gene;Acc:115570751]	**F08_FMdiet_D15_F08_FMdiet_D30**
LOC115576212	-1.908499943	3.14492E-16	2.41111E-15	DOWN	hemoglobin subunit beta-2-like [Source:NCBI gene;Acc:115576212]	
LOC115576218	-1.722761837	3.00124E-15	1.72572E-14	DOWN	hemoglobin subunit alpha-1 [Source:NCBI gene;Acc:115576218]	
ca5a	1.602552413	8.49913E-12	3.258E-11	UP	carbonic anhydrase 5A [Source:NCBI gene;Acc:115586666]	
ca6	0.976626565	1.01561E-05	2.3359E-05	UP	carbonic anhydrase 6 [Source:NCBI gene;Acc:115584806]	
LOC115568977	0.961776119	0.000184664	0.000303377	UP	sodium bicarbonate transporter-like protein 11 [Source:NCBI gene;Acc:115568977]	
LOC115570135	2.009299401	2.97863E-21	2.28362E-20	UP	carbonic anhydrase 1-like [Source:NCBI gene;Acc:115570135]	
LOC115570670	-3.121287564	1.28642E-44	2.95876E-43	DOWN	hemoglobin subunit alpha-1-like [Source:NCBI gene;Acc:115570670]	
LOC115570751	-2.226102229	3.83968E-25	4.41564E-24	DOWN	hemoglobin subunit beta-1-like [Source:NCBI gene;Acc:115570751]	
LOC115576210	-2.294780833	0.000819566	0.001256668	DOWN	hemoglobin subunit beta-like [Source:NCBI gene;Acc:115575823]	
LOC115576212	-0.883531909	7.25159E-05	0.000128297	DOWN	hemoglobin subunit beta-2-like [Source:NCBI gene;Acc:115576212]	
LOC115576214	0.836443686	4.95855E-05	9.50389E-05	UP	hemoglobin cathodic subunit beta-like [Source:NCBI gene;Acc:115576214]	**F15_PPdiet_D15_F15_PPdiet_D30**
LOC115576218	-1.309093364	1.00142E-09	2.87907E-09	DOWN	hemoglobin subunit alpha-1 [Source:NCBI gene;Acc:115576218]	
LOC115576321	1.452947527	3.22343E-12	1.48278E-11	UP	band 3 anion exchange protein-like [Source:NCBI gene;Acc:115576321]	
LOC115578684	2.362697809	4.64484E-13	2.67078E-12	UP	sodium bicarbonate cotransporter 3-like [Source:NCBI gene;Acc:115578684]	
LOC115588298	1.351221313	1.33666E-08	3.41592E-08	UP	glucose-6-phosphate exchanger SLC37A1-like [Source:NCBI gene;Acc:115588298]	
LOC115592532	1.049135006	0.008680466	0.011091707	UP	electrogenic sodium bicarbonate cotransporter 4-like [Source:NCBI gene;Acc:115592532]	
slc26a5	1.543858612	9.76435E-11	3.20829E-10	UP	solute carrier family 26 member 5 [Source:NCBI gene;Acc:115595297]	
slc37a2	1.570444179	0.00108952	0.001566185	UP	solute carrier family 37 member 2 [Source:NCBI gene;Acc:115594661]	
slc4a11	1.202140602	1.28756E-05	2.69217E-05	UP	solute carrier family 4 member 11 [Source:NCBI gene;Acc:115597494]	
LOC115570135	0.687733121	0.0008325	0.002735357	UP	carbonic anhydrase 1-like [Source:NCBI gene;Acc:115570135]	**F15_PPdiet_D15_F15_FMdiet_D15**
LOC115570670	-1.091081685	1.43627E-07	3.30342E-06	DOWN	hemoglobin subunit alpha-1-like [Source:NCBI gene;Acc:115570670]	

LOC115570751	-0.92442158	7.87617E-06	6.0384E-05	DOWN	hemoglobin subunit beta-1-like [Source:NCBI gene;Acc:115570751]	
LOC115576209	-0.699625644	0.000653931	0.002735357	DOWN	hemoglobin subunit beta-like [Source:NCBI gene;Acc:115576209]	
LOC115576218	-0.691490302	0.001017223	0.002924516	DOWN	hemoglobin subunit alpha-1 [Source:NCBI gene;Acc:115576218]	
LOC115576321	0.941668376	4.93491E-06	5.67515E-05	UP	band 3 anion exchange protein-like [Source:NCBI gene;Acc:115576321]	
LOC115578684	1.230598243	0.000755019	0.002735357	UP	sodium bicarbonate cotransporter 3-like [Source:NCBI gene;Acc:115578684]	
slc26a5	0.619462846	0.011229156	0.025827058	UP	solute carrier family 26 member 5 [Source:NCBI gene;Acc:115595297]	
slc4a11	1.096363158	6.51669E-05	0.00037471	UP	solute carrier family 4 member 11 [Source:NCBI gene;Acc:115597494]	
LOC115576210	3.505013972	1.05991E-09	2.43779E-08	UP	hemoglobin subunit beta-like [Source:NCBI gene;Acc:115575823]	**F15_PPdiet_D30_F15_FMdiet_D30**
LOC115570670	-2.713936408	5.5293E-35	1.27174E-33	DOWN	hemoglobin subunit alpha-1-like [Source:NCBI gene;Acc:115570670]	
LOC115570751	-2.050172378	1.17746E-21	1.35408E-20	DOWN	hemoglobin subunit beta-1-like [Source:NCBI gene;Acc:115570751]	**F15_FMdiet_D15_F15_FMdiet_D30**
LOC115576212	-1.638800334	1.57336E-12	1.20624E-11	DOWN	hemoglobin subunit beta-2-like [Source:NCBI gene;Acc:115576212]	
LOC115576218	-1.485166301	1.52947E-11	8.79443E-11	DOWN	hemoglobin subunit alpha-1 [Source:NCBI gene;Acc:115576218]	
ca5a	1.616374138	1.53539E-11	7.06279E-11	UP	carbonic anhydrase 5A [Source:NCBI gene;Acc:115586666]	
LOC115568977	1.392552158	1.34558E-06	3.26028E-06	UP	sodium bicarbonate transporter-like protein 11 [Source:NCBI gene;Acc:115568977]	
LOC115570135	1.92197478	1.14316E-19	8.76424E-19	UP	carbonic anhydrase 1-like [Source:NCBI gene;Acc:115570135]	
LOC115570670	-3.119920441	8.88287E-45	2.04306E-43	DOWN	hemoglobin subunit alpha-1-like [Source:NCBI gene;Acc:115570670]	
LOC115570751	-2.722705416	1.41897E-35	1.63182E-34	DOWN	hemoglobin subunit beta-1-like [Source:NCBI gene;Acc:115570751]	
LOC115576212	-1.397998205	4.93862E-10	1.62269E-09	DOWN	hemoglobin subunit beta-2-like [Source:NCBI gene;Acc:115576212]	
LOC115576214	0.696225762	0.00071735	0.001269157	UP	hemoglobin cathodic subunit beta-like [Source:NCBI gene;Acc:115576214]	**F17_PPdiet_D15_F17_PPdiet_D30**
LOC115576218	-1.728368921	1.21536E-15	6.9883E-15	DOWN	hemoglobin subunit alpha-1 [Source:NCBI gene;Acc:115576218]	
LOC115576321	1.372235006	4.28583E-11	1.6429E-10	UP	band 3 anion exchange protein-like [Source:NCBI gene;Acc:115576321]	
LOC115578684	1.763079795	1.41751E-06	3.26028E-06	UP	sodium bicarbonate cotransporter 3-like [Source:NCBI gene;Acc:115578684]	
LOC115588298	1.455713589	3.42105E-09	9.83551E-09	UP	glucose-6-phosphate exchanger SLC37A1-like [Source:NCBI gene;Acc:115588298]	
LOC115594511	3.026076979	4.67603E-05	8.96239E-05	UP	carbonic anhydrase 4-like [Source:NCBI gene;Acc:115594511]	
slc26a5	1.084351385	5.82325E-06	1.21759E-05	UP	solute carrier family 26 member 5 [Source:NCBI gene;Acc:115595297]	
slc4a11	0.805399624	0.005561316	0.009136448	UP	solute carrier family 4 member 11 [Source:NCBI gene;Acc:115597494]	
ca5a	0.966427983	6.79061E-05	0.00039046	UP	carbonic anhydrase 5A [Source:NCBI gene;Acc:115586666]	
LOC115568977	1.055426313	0.000238414	0.000783361	UP	sodium bicarbonate transporter-like protein 11 [Source:NCBI gene;Acc:115568977]	**F17_PPdiet_D15_F17_FMdiet_D15**
LOC115570135	1.488121939	1.11396E-12	1.28106E-11	UP	carbonic anhydrase 1-like [Source:NCBI gene;Acc:115570135]	

LOC115576210	1.127713209	0.004597086	0.010573298	UP	hemoglobin subunit beta-like [Source:NCBI gene;Acc:115575823]	
LOC115576321	1.567660622	6.67689E-14	1.53568E-12	UP	band 3 anion exchange protein-like [Source:NCBI gene;Acc:115576321]	
LOC115578684	1.588801478	1.35612E-05	0.000103969	UP	sodium bicarbonate cotransporter 3-like [Source:NCBI gene;Acc:115578684]	
LOC115588298	0.957554186	0.000110759	0.000483403	UP	glucose-6-phosphate exchanger SLC37A1-like [Source:NCBI gene;Acc:115588298]	
LOC115594511	2.774355498	0.000126105	0.000483403	UP	carbonic anhydrase 4-like [Source:NCBI gene;Acc:115594511]	
slc26a5	0.758973336	0.001489935	0.003807611	UP	solute carrier family 26 member 5 [Source:NCBI gene;Acc:115595297]	
slc4a11	0.9991742	0.000379268	0.001090395	UP	solute carrier family 4 member 11 [Source:NCBI gene;Acc:115597494]	
No DEGs were observed in this comparison						**F17_PPdiet_D30_F17_FMdiet_D30**
LOC115570670	-2.872544042	6.2105E-39	1.42842E-37	DOWN	hemoglobin subunit alpha-1-like [Source:NCBI gene;Acc:115570670]	**F17_FMdiet_D15_F17_FMdiet_D30**
LOC115570751	-2.521337832	3.52839E-31	4.05765E-30	DOWN	hemoglobin subunit beta-1-like [Source:NCBI gene;Acc:115570751]	
LOC115576212	-2.02490003	1.54562E-18	8.88733E-18	DOWN	hemoglobin subunit beta-2-like [Source:NCBI gene;Acc:115576212]	
LOC115576218	-2.089741109	4.17566E-21	3.20134E-20	DOWN	hemoglobin subunit alpha-1 [Source:NCBI gene;Acc:115576218]	
ca5a	1.222968058	1.89102E-07	4.8326E-07	UP	carbonic anhydrase 5A [Source:NCBI gene;Acc:115586666]	**F20_PPdiet_D15_F20_PPdiet_D30**
ca6	0.650066565	0.003725263	0.006590849	UP	carbonic anhydrase 6 [Source:NCBI gene;Acc:115584806]	
LOC115568977	2.115044496	2.02472E-14	1.16421E-13	UP	sodium bicarbonate transporter-like protein 11 [Source:NCBI gene;Acc:115568977]	
LOC115570135	1.505312855	5.74551E-13	2.64293E-12	UP	carbonic anhydrase 1-like [Source:NCBI gene;Acc:115570135]	
LOC115570670	-3.926133897	2.97849E-65	6.85053E-64	DOWN	hemoglobin subunit alpha-1-like [Source:NCBI gene;Acc:115570670]	
LOC115570751	-3.551099575	1.71533E-55	1.97263E-54	DOWN	hemoglobin subunit beta-1-like [Source:NCBI gene;Acc:115570751]	
LOC115576210	-2.499020347	0.00023063	0.000482227	DOWN	hemoglobin subunit beta-like [Source:NCBI gene;Acc:115575823]	
LOC115576212	-1.326173093	3.41586E-09	1.30941E-08	DOWN	hemoglobin subunit beta-2-like [Source:NCBI gene;Acc:115576212]	
LOC115576218	-1.855784698	6.96252E-18	5.33793E-17	DOWN	hemoglobin subunit alpha-1 [Source:NCBI gene;Acc:115576218]	
LOC115576321	1.120585972	6.0733E-08	1.74607E-07	UP	band 3 anion exchange protein-like [Source:NCBI gene;Acc:115576321]	
LOC115578684	1.4908525	7.56587E-06	1.74015E-05	UP	sodium bicarbonate cotransporter 3-like [Source:NCBI gene;Acc:115578684]	
LOC115588298	1.37624253	4.75547E-08	1.56251E-07	UP	glucose-6-phosphate exchanger SLC37A1-like [Source:NCBI gene;Acc:115588298]	
slc26a5	0.687075839	0.004522891	0.007430464	UP	solute carrier family 26 member 5 [Source:NCBI gene;Acc:115595297]	
slc37a2	1.51583496	0.013197567	0.018971503	UP	solute carrier family 37 member 2 [Source:NCBI gene;Acc:115594661]	
slc4a11	1.254166993	0.000362155	0.00069413	UP	solute carrier family 4 member 11 [Source:NCBI gene;Acc:115597494]	
LOC115570135	0.752669002	0.000251675	0.001157707	UP	carbonic anhydrase 1-like [Source:NCBI gene;Acc:115570135]	**F20_PPdiet_D15_F20_FMdiet_D15**
LOC115576212	0.858785013	4.99563E-05	0.000287249	UP	hemoglobin subunit beta-2-like [Source:NCBI gene;Acc:115576212]	
LOC115576321	1.071211731	2.17766E-07	5.00861E-06	UP	band 3 anion exchange protein-like [Source:NCBI gene;Acc:115576321]	
LOC115594511	2.200120338	1.35258E-06	1.55547E-05	UP	carbonic anhydrase 4-like [Source:NCBI gene;Acc:115594511]	

slc4a11	1.424114147	1.41963E-05	0.000108839	UP	solute carrier family 4 member 11 [Source:NCBI gene;Acc:115597494]	
LOC115568977	-0.934813855	0.000360331	0.008287618	DOWN	sodium bicarbonate transporter-like protein 11 [Source:NCBI gene;Acc:115568977]	**F20_PPdiet_D30_F20_FMdiet_D30**
LOC115570670	-3.519732631	8.98851E-55	2.06736E-53	DOWN	hemoglobin subunit alpha-1-like [Source:NCBI gene;Acc:115570670]	
LOC115570751	-3.146185342	1.5987E-45	1.83851E-44	DOWN	hemoglobin subunit beta-1-like [Source:NCBI gene;Acc:115570751]	
LOC115576212	-2.370754376	1.23833E-25	7.1204E-25	DOWN	hemoglobin subunit beta-2-like [Source:NCBI gene;Acc:115576212]	**F20_FMdiet_D15_F20_FMdiet_D30**
LOC115576218	-2.492488445	5.14722E-30	3.94621E-29	DOWN	hemoglobin subunit alpha-1 [Source:NCBI gene;Acc:115576218]	
LOC115594511	-1.863681116	5.92257E-05	0.000272438	DOWN	carbonic anhydrase 4-like [Source:NCBI gene;Acc:115594511]	
